# Effects of Supporting Electrolyte and Solvent on the Thermodynamics and Kinetics of Reduction Reactions in a Keggin-Type Polyoxotungstate

**DOI:** 10.1021/acs.inorgchem.6c01354

**Published:** 2026-06-04

**Authors:** Hania A. Guirguis, Mamta Dagar, Sophia M. Anderson, Ellen M. Matson, Agnes E. Thorarinsdottir

**Affiliations:** Department of Chemistry, 6927 University of Rochester, Rochester, New York 14627, United States

## Abstract

We report the thermodynamic and kinetic behavior of reduction reactions of the Keggin-type polyoxometalate (POM) [PW_12_O_40_]^3–^ ((PW_12_)^3–^) in the presence of TBAPF_6_, KPF_6_, and LiPF_6_ supporting electrolytes. Room-temperature electrochemical analyses of (TBA)_3_(PW_12_) (TBA^+^ = tetrabutylammonium) reveal anodic shifts of the second and third reduction potentials in the presence of KPF_6_ and LiPF_6_ in acetonitrile (MeCN) and *N*,*N*-dimethylformamide (DMF), arising from ion-pairing interactions between the reduced charge states of the POM and alkali cations. Variable-temperature cyclic voltammetry analyses reveal ion-pairing behavior between alkali cations and the most reduced charge states of the POM, and associated disruption of POM solvation shell, to shift redox entropies (Δ*S*_redox_) positively as compared to the cation-uncoupled (PW_12_)^3–^/(PW_12_)^4–^ reduction. Collectively, these results highlight that tuning the identity of supporting electrolyte cation and solvent can influence both the kinetics and thermodynamics of electron transfer in POMs, providing experimental handles for controlling electrochemical properties for targeted applications in electrochemistry.

## Graphical abstract

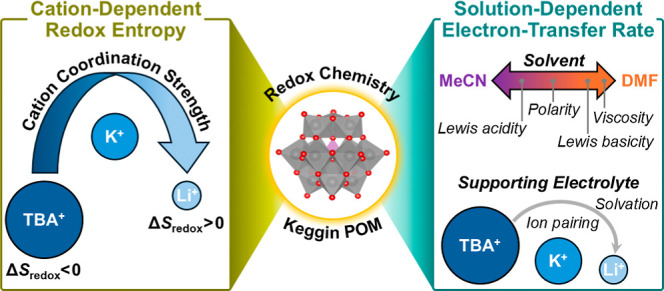

## Introduction

The outcome of electrochemical reactions is sensitive to the composition of the electrolyte solution, including solvent and supporting electrolyte salt, as well as physical properties such as temperature and pressure.^1–5^ For example, the type of solvent and/or supporting electrolyte has been shown to influence the rate and selectivity of various electrochemical small molecule activation reactions of energy relevance^1–10^ and electroorganic syntheses.^11,12^ The primary reasons for observed modulations in reaction rates, selectivity, and efficiency as a result of changes in solution environments arise from variations in local electric field strengths, stabilization of select reaction intermediates, and minimization of detrimental side reactions.^13–16^ Despite the potential of electrolyte solution engineering in improving the performance of electrochemical systems toward targeted reactions, the community lacks a detailed understanding of how solution conditions influence the electrochemical behavior of electrocatalysts and redox mediators.

Polyoxometalates (POMs) represent a class of redox-active inorganic compounds that have been demonstrated to function as electrocatalysts and redox mediators in electrochemical transformations.^17–24^ The utility of these molecular metal oxide assemblies is rooted in their rich redox properties and stability.^20,25–28^ For example, the phosphorus-centered Keggin-type polyoxotungstate [PW_12_O_40_]^3–^ ((PW_12_)^3–^) exhibits four quasi-reversible, one-electron redox events in MeCN, with potentials spanning from 0 V to −2.5 V vs Ag/AgNO_3_ (Figure S1).^24,29^ The ability of POMs to undergo multiple electron-transfer processes, coupled to their anionic charge, renders these compounds attractive candidates for studying the influence of solution environment on electrochemical properties. Indeed, the electrochemical behavior of Keggin-type POMs has been studied in both aqueous and organic solutions, as well as in the presence of coordinating and noncoordinating cations through experiments and computations.^22,27,30–35^ Collectively, these studies have demonstrated that the extent of cation–POM interactions is heavily influenced by solvent, as well as the size and identity of countercation, underscoring that modulating the nature of the electrolyte solution is an approach for tuning the redox properties of POMs.

An alternative parameter that can tune the redox properties of POMs is the temperature of the electrolyte solution. The solution temperature may influence the redox behavior of molecular species through altering both kinetic and thermodynamic properties, including charge-transfer rates, diffusion rates, reduction potentials, and solvation structures, in addition to modulating solution resistance, and the solubility and stability of redox-active species.^36–40^ Variable-temperature cyclic voltammetry (VT-CV) is a useful tool for assessing how redox potentials vary with temperature (as quantified by the temperature coefficient of the formal potential, α) and quantifying entropic contributions to the thermodynamics of charge-transfer processes (as quantified by the redox entropy, Δ*S*_redox_).^41–47^ The redox entropy describes the change in (dis)­order during a redox reaction and serves as a critical parameter for evaluating the influence of temperature and electrolyte solution on the electrochemical properties of molecular species. We note that Δ*S*_redox_ originates from changes in both geometric and solution structures of the redox-active species and is thus influenced by interactions with solvent molecules and electrolyte ions.^46,48,49^

Inspired by studies on the impact of cation association and solvation on the electrochemistry of POMs,^30–32,34,50^ together with our previous work on the effects of charge and heteroatom dopants on the thermodynamics and kinetics of redox reactions in Keggin-type POMs,^29^ we sought to understand how temperature may influence cation–POM interactions and the associated electrochemical properties of POMs (Figure 1). Furthermore, we wanted to assess whether electrolyte solution engineering can be employed as a tool to modulate the redox entropy and electron-transfer kinetics of POMs for targeted electrochemical applications. Herein, we report the thermodynamics and kinetics of reduction reactions of the tetrabutylammonium (TBA^+^) salt of the Keggin-type polyoxotungstate anion [PW_12_O_40_]^3–^ ((PW_12_)^3–^) in different organic solvents (acetonitrile, MeCN; *N*,*N*-dimethylformamide, DMF), and in the presence of various supporting electrolyte cations (TBA^+^, K^+^, Li^+^; Figure 1). Our results demonstrate that the redox entropy for the formally (PW_12_)^4–^/(PW_12_)^5–^ couple is sensitive to the identity of countercation in solution, with values switching from negative to positive as the supporting electrolyte cation is changed from noncoordinating TBA^+^ to the most coordinating Li^+^ in MeCN. Solvent identity influences both exact redox entropy values and the extent of variation offered by different alkali cations, as well as electron-transfer rates. The fundamental understanding of the thermodynamics and kinetics of electron transfer in a Keggin-type polyoxotungstate gained from our study informs the design of electrochemical systems based on POMs for targeted applications in electrochemistry, including as homogeneous electrocatalysts and redox mediators.

**Figure 1:**
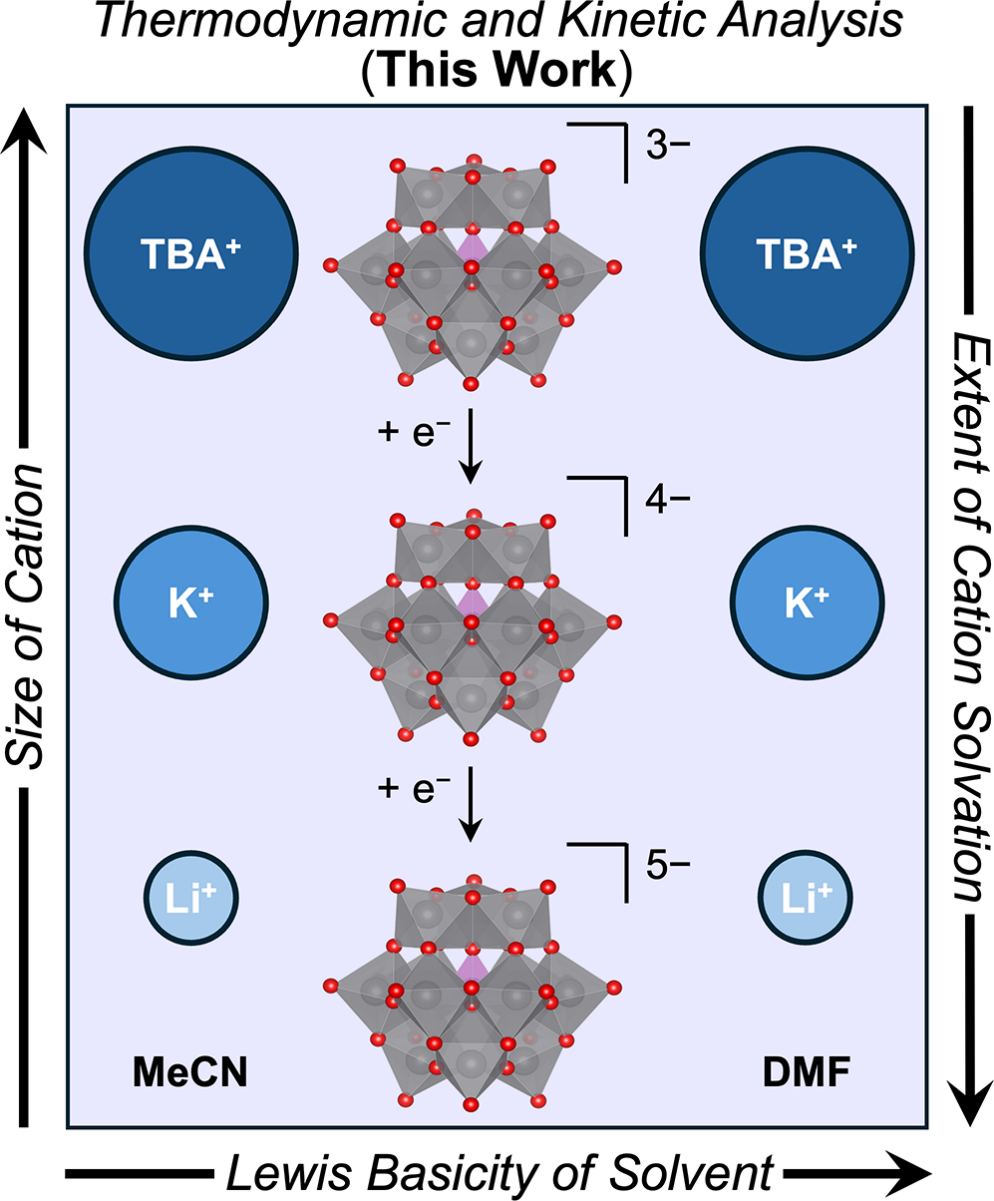
Overview of the cations, solvents, and different charge states of (PW_12_)^*n*−^ (note that *n*– is used instead of 3− when referring to the generic compound in different charge states) used in this study. The trends in the extent of cation solvation were adapted from ref 34.

## Experimental Section

### General Considerations

Unless otherwise indicated, experiments were carried out in an MBraun UNIlab glovebox under an atmosphere of dinitrogen in the absence of water and oxygen. Aside from variable-temperature electrochemical analyses (vide infra), all manipulations were performed at room temperature (∼19–21 °C). Glassware was oven-dried for at least 4 h and cooled under dynamic vacuum in the antechamber prior to use. MeCN and DMF were dried and deoxygenated on a solvent purification system (Pure Process Technology, LLC). Solvents were stored for at least 24 h over activated 3 Å molecular sieves (Fisher Scientific) inside the glovebox prior to use; water content of these solvents was determined to be <5 ppm by Karl Fischer titration. Tetrabutylammonium hexafluorophosphate (TBAPF_6_) was purchased from Millipore Sigma, recrystallized three times using hot ethanol and dried under dynamic vacuum at 120 °C for 2 days prior to use. Anhydrous potassium hexafluorophosphate (KPF_6_) and lithium hexafluorophosphate (LiPF_6_) were purchased from Millipore Sigma and used as received. (TBA)_3_[PW_12_O_40_] ((TBA)_3_(PW_12_)) was synthesized according to a literature procedure.^33^

### General Electrochemical Measurements

All electrochemical measurements were carried out under dinitrogen atmosphere in a MBraun UNIlab glovebox using a BioLogic SP-150 potentiostat. Data analysis was performed using the EC-Lab software (version 11.42). Samples of (TBA)_3_(PW_12_) analyte were prepared using dry MeCN and DMF solvents containing 100 mM of supporting electrolyte (TBAPF_6_, KPF_6_, LiPF_6_) unless otherwise stated. For all experiments, the concentration of the (TBA)_3_(PW_12_) analyte was 1 mM. Unless otherwise specified, experiments were carried out in a single-compartment cell (5 mL) using a glassy carbon working electrode (CH Instruments, Inc.), platinum wire auxiliary electrode (CH Instruments, Inc.), and nonaqueous Ag/AgNO_3_ reference electrode (BASi) filled with a MeCN solution containing 10 mM of AgNO_3_ and 100 mM of TBAPF_6_. Before use, the glassy carbon working electrode was polished with alumina powder (1.0 μm, CH Instruments, Inc.) on a microfiber polishing cloth, and the platinum auxiliary electrode was cleaned by rinsing with 6 M hydrochloric acid solution in ethanol (Koptec's 200 proof pure ethanol; Decon Labs, Inc.) followed by distilled water and acetone, and left to dry in air for 1 h prior to further drying in an evacuated antechamber for at least 1 h. All glassware used for electrochemical analyses was rinsed with 6 M hydrochloric acid solution in ethanol (Koptec's 200 proof pure ethanol; Decon Labs, Inc.) and distilled water, and oven-dried for at least 4 h prior to use.

Cyclic voltammetry (CV) measurements were conducted using a positive scan direction for four cycles and a scan rate of 100 mV s^–1^ unless otherwise stated. The third CV cycle is displayed in all cases. A potential window of −0.3 V to −1.4 V vs Ag/AgNO_3_ was selected for MeCN solutions containing TBAPF_6_ and LiPF_6_ supporting electrolytes. A smaller potential window of −0.3 V to −1.1 V vs Ag/AgNO_3_ was selected for best resolution of the data collected in MeCN containing KPF_6_ supporting electrolyte; additional reduction events that reduce the reversibility of the redox processes of interest in this study are observed between −1.0 V and −1.4 V vs Ag/AgNO_3_ (Figure S2). The potential window was extended cathodically to up to −1.8 V vs Ag/AgNO_3_ for sample solutions in DMF to allow for complete analysis of the same redox processes as observed in MeCN. Solutions were allowed to equilibrate for 5 min with stirring prior to CV data collection in the absence of stirring. For variable-scan-rate CV measurements of specific reduction events (R1, R2, or R3), the potential was initially scanned negatively from the open-circuit potential (*E*_OCP_) to the potential range of interest, and then subsequently scanned using a positive scan direction for four cycles in a narrow potential window around the given redox couple. The third CV cycle is displayed in all cases. All potentials are referenced to the Ag/AgNO_3_ reference electrode potential. Note that drifts in the reference electrode potential were checked routinely using the ferrocenium/ferrocene (Fc^+^/Fc) redox couple and assessed to be <2 mV. Furthermore, CV analysis of Fc was used to assess the upper limit of liquid-junction potential that may form due to difference in the composition of the inner electrolyte solution of the Ag/AgNO_3_ reference electrode (10 mM of AgNO_3_, 100 mM of TBAPF_6_, MeCN) and the outer electrolyte solution. Specifically, cyclic voltammograms were collected following the method outlined above using 1 mM of Fc and 100 mM of supporting electrolyte (TBAPF_6_, KPF_6_, LiPF_6_) in MeCN or DMF. Comparison of *E*_1/2_ values of the Fc^+^/Fc redox couple across the different solutions revealed <9 mV and <20 mV difference as the supporting electrolyte cation and solvent were changed, respectively (Table S1). While the slight variation in *E*_1/2_ values could, at least in part, be due to differences in interactions of the Fc^+^ and Fc redox species with the different supporting electrolyte cations and/or solvent molecules, we establish an upper limit for the liquid-junction potential of 20 mV. Uncompensated solution resistance (*R*_u_) was determined to be ∼150 Ω in MeCN and ∼300 Ω in DMF using the ZIR tool within the EC-Lab software at 100 kHz. The CV data were *iR* compensated at 85%.

Square-wave voltammetry (SWV) measurements were carried out using the same setup as described for standard CV measurements (vide supra). Square-wave voltammograms for MeCN solutions were scanned in the positive direction in a potential window of −0.3 V to −1.5 V vs Ag/AgNO_3_ with pulse height of 25 mV, pulse width of 100 ms, and step height of 10 mV (statistically identical results were obtained using step height of 5 mV). The potential window was extended cathodically to up to −1.8 V vs Ag/AgNO_3_ for sample solutions in DMF to allow for complete analysis of the same redox processes as observed in MeCN. Solutions were allowed to equilibrate for 5 min with stirring prior to SWV data collection in the absence of stirring. All potentials are referenced to the Ag/AgNO_3_ reference electrode potential. In all ion titration experiments, the concentration of the (TBA)_3_(PW_12_) analyte was 1 mM and the concentration of TBAPF_6_ supporting electrolyte was 100 mM. KPF_6_ and LiPF_6_ were added to the sample solution as solid amounts to reach the desired equivalents (0, 1, 5, 10, 25, 50, 75,100) relative to (TBA)_3_(PW_12_) and solutions were stirred for 1 min prior to data collection in the absence of stirring. The difference in *E*_1/2_ values (Δ*E*_1/2_) for the first reduction (R1) of (TBA)_3_(PW_12_) between sample solutions in the presence and absence of MPF_6_ (M = K^+^, Li^+^) is minimal (up to ∼20 mV) in both MeCN and DMF and can be largely ascribed to the change in ionic strength of the sample solutions (up to 100 mM difference), as determined by SWV measurements of 1 mM of (TBA)_3_(PW_12_) in MeCN and DMF containing 25–200 mM of TBAPF_6_ supporting electrolyte (Figures S3 and S4). These data show anodic shifts of the first two reductions of (TBA)_3_(PW_12_) by ∼10 mV and ∼20 mV in MeCN and DMF, respectively. In contrast, values of Δ*E*_1/2_ for the second reduction (R2) of (TBA)_3_(PW_12_) between sample solutions in the presence and absence of MPF_6_ (M = K^+^, Li^+^) show significant anodic shifts in both MeCN and DMF (vide infra). These values were used to estimate effective equilibrium constants for the association of alkali cations (K^+^, Li^+^) with the (PW_12_)^5–^ anion. The effective ion association constants (*K*_a_) were obtained through nonlinear least-squares fitting of Δ*E*_1/2_ vs [M^+^] data using a Langmuir model (eq 1)^51^ and the Solver tool in Microsoft Excel. The Langmuir model is a 1:1 binding model, where each alkali cation interacts with one independent binding site on the surface of the polyoxotungstate anion. According to the model, all binding sites are identical and energetically equivalent.^52,53^ The high symmetry and high degree of charge delocalization of the polyoxotungstate anion^25–28^ render the Langmuir model well-suited for assessing the relative strength of interactions between cations and the negatively charged polyoxotungstate surface in different solvents, particularly in the presence of high concentrations of alkali ions (vide infra), although absolute values of *K*_a_ are only an approximation. We note that the *K*_a_ values extracted from this analysis reflect both the binding strength of the ion and the magnitude of the shift of *E*_1/2_ per bound cation–POM pair.

1 $$\Delta E_{1/2}=\frac{K_{a}\left[M^{+}\right]}{1+K_{a}\left[M^{+}\right]}\cdot {\Delta E}_{1/2,\max}$$

In this equation, [M^+^] denotes the concentration of added alkali metal electrolyte salt (KPF_6_ or LiPF_6_), and Δ*E*_1/2,max_ corresponds to the maximum observed anodic shift of *E*_1/2_ in the presence of MPF_6_ (M = K^+^, Li^+^). Error bars for listed values of *K*_a_ denote the standard deviation of measurements of at least two independently prepared samples.

All electrochemical data are plotted according to the IUPAC convention, where negative currents correspond to cathodic reactions (reduction) and positive currents correspond to anodic reactions (oxidation). Independently prepared samples of each type were measured to ensure reproducibility. The provided data are representative examples.

### Electrochemical Kinetics

Diffusion coefficients associated with the first two reductions of (PW_12_)^3–^ (R1, R2) in solutions containing TBAPF_6_ or KPF_6_ and the first three reductions (R1, R2, R3) in solutions containing LiPF_6_ were determined at room temperature (∼19–21 °C) from variable-scan-rate CV measurements (10–10,000 mV s^–1^) following the same protocol as described above. The CV data were *iR* compensated at 95% using the ZIR tool within the EC-Lab software at 100 kHz. The remaining 5% uncompensated solution resistance was accounted for by manual correction.^54^ Samples of (TBA)_3_(PW_12_) analyte (1 mM) were prepared using dry MeCN and DMF solvents containing 100 mM of supporting electrolyte (TBAPF_6_, KPF_6_, LiPF_6_). The anodic and cathodic diffusion coefficients of each redox couple were separately quantified from the anodic and cathodic peak currents, respectively, using Randles–Ševčík analysis.^55,56^ Specifically, the diffusion coefficients were estimated using the slopes of the linear fits to the data in plots of peak current vs the square root of scan rate. As the redox couples of the POM are not strictly reversible, rather quasi-reversible (peak potential separation is >59 mV per electron transfer at room temperature and increases slightly with scan rate), the diffusion coefficients were separately estimated using the Randles–Ševčík equations for a fully reversible redox couple (eq 2) and an irreversible redox couple (eq 3) at 20 °C:^55–57^

2 $$i_{p}=2.71\times 10^{5}n^{3/2}AcD_{0}^{1/2}\nu^{1/2}$$

3 $$i_{p}=3.01\times 10^{5}n^{3/2}\alpha_{CT}^{1/2}AcD_{0}^{1/2}\nu^{1/2}$$

In these equations, *i*_p_ is the peak current (anodic or cathodic), *n* is the number of electrons transferred in the given reaction, *A* is the geometric surface area of the glassy carbon working electrode (cm^2^), *c* is the concentration of the redox-active species in the bulk solution (mol cm^–3^), *D*_0_ is the diffusion coefficient (cm^2^ s^–1^), *v* is the scan rate (V s^–1^), and α_CT_ is the charge-transfer coefficient. In this study, α_CT_ was assumed to be 0.5 owing to the electrochemical symmetry of the investigated redox couples.^55–57^ For redox couples that show quasi-reversible kinetics, relationships for both reversible and irreversible redox reaction are often employed to determine the diffusion coefficients.^55–58^ The true values of the diffusion coefficients are expected to be within the ranges suggested by eqs 2 and 3.

The standard heterogeneous electron-transfer rate constant (*k*_0_) was estimated using the Nicholson method for quasi-reversible redox reactions (eq 4).^58^ Specifically, the potential difference (Δ*E*_p_) between oxidation and reduction peaks was obtained as a function of scan rate, and values of the transfer parameter (ψ) were extracted directly using the mathematical relationship reported by Sawant and McKone using Δ*E*_p_ values (eq 5).^59^

4 $$\psi =\nu^{-1/2}k_{0}{\left(\pi nFD_{0}/RT\right)}^{-1/2}$$

5 $$\Delta E_{p}\times n=0.054+0.03103\psi^{-0.7078}$$

In these equations, *F* is Faraday’s constant, *R* is the ideal gas constant, *T* is the temperature (taken to be 20 °C for all analyses), and all other parameters are as defined in the text above with *k*_0_ in units of cm s^–1^. Herein, we report *k*_0_ values calculated using cathodic reversible diffusion coefficients (eq 2), cathodic irreversible diffusion coefficients (eq 3), and an average of the reversible and irreversible values.

### Variable-Temperature Electrochemical Measurements of (PW_12_)^3–^

Following reported protocols,^29,44,47^ the temperature coefficient (α) of each redox couple of (PW_12_)^3–^ in the investigated potential window (vide supra) was estimated using variable-temperature CV measurements in an isothermal electrochemical setup. The measurements were carried out in a single-compartment glass cell using the same setup as described for room-temperature CV measurements (vide supra) with the addition of a temperature control. The temperature of the solution near the working electrode was controlled using a hot plate and quantified using a thermocouple (stainless steel) immersed in the analyte solution and kept at the same height as the working and reference electrodes. The thermocouple was calibrated using an external temperature controller (Omega Engineering CS8DPT). The solution temperature was increased in ∼4 °C intervals in the temperature range ∼20–42 °C. At each temperature, the cell was allowed to equilibrate for ∼7 min before four CV cycles were collected at the given temperature. Solutions were stirred at 600 rpm between measurements at different temperatures. In all experiments, the concentration of the (TBA)_3_(PW_12_) analyte was 1 mM, the concentration of supporting electrolyte (TBAPF_6_, KPF_6_, LiPF_6_) was 100 mM, and the solution volume was 5 mL. Half-wave potentials (*E*_1/2_) for each redox couple of (PW_12_)^3–^ were extracted from the variable-temperature CV data and plotted against temperature in °C. As the diffusion coefficients for the oxidized and reduced species of individual redox couples in given solution conditions are similar (Tables S2–S5), we make the estimation that the formal potential and the half-wave potential are approximately equal (*E* ^0′^ ≈ *E*_1/2_),^57^ thus the slopes of the linear fits to the data of *E*_1/2_ vs temperature plots afford the temperature coefficients of the redox couples with respect to the reference electrode potential. Error bars denote the standard deviation of measurements of independently prepared samples. Note, however, that the potential of the Ag/AgNO_3_ reference electrode is also sensitive to temperature.^60^ Accordingly, the true temperature coefficient of a given redox couple is obtained after correcting for the temperature coefficient of the reference electrode potential using the following equations:^60^

6 $$\frac{{\partial E}_{1/2}}{\partial T}=\frac{{\partial E}_{1/2‐meas}}{\partial T}+\frac{{\partial E}_{ref}}{\partial T}$$

7 $$\alpha =\alpha_{meas}+\alpha_{ref}$$

In these equations, *T* is the temperature, *E*_1/2‑meas_ is the measured half-wave potential, *E*_ref_ is the potential of the reference electrode, α is the true temperature coefficient, α_meas_ is the measured temperature coefficient, and α_ref_ is the temperature coefficient of the reference electrode potential (vide infra).

The temperature coefficient values for the redox couples of the POM were used to estimate the associated redox reaction entropies (Δ*S*_redox_) using the following equation:^41,46^

8 $$\Delta S_{redox}=S_{red}-S_{ox}=nF\alpha$$

In this equation, *S*_red_ and *S*_ox_ denote the partial molar entropies of the reduced and oxidized species, respectively, *n* is the number of electrons transferred in the given reaction, *F* is Faraday’s constant, and α is the corrected temperature coefficient.

### Variable-Temperature Electrochemical Measurements of Reference Electrode Potential

The temperature coefficient of the nonaqueous Ag/AgNO_3_ reference electrode potential was estimated using nonisothermal open-circuit potential (*E*_OCP_) measurements in a two-electrode setup following a reported protocol where one Ag/AgNO_3_ electrode served as the working electrode and another served as the reference and auxiliary electrodes.^29,44,47,60^ The inner solution of the Ag/AgNO_3_ reference electrode was kept as 10 mM of AgNO_3_ and 100 mM of TBAPF_6_ in MeCN for all measurements. The measurements were carried out in a three-compartment custom-made glass cell (Adams & Chittenden Scientific) with fine frits separating the two side compartments from the middle compartment. For each measurement, 9 mL and 4 mL of solvent (MeCN, DMF) containing 100 mM of supporting electrolyte (TBAPF_6_, KPF_6_, LiPF_6_) were added to the side and middle compartments, respectively. The cell was arranged such that one of the side compartments was placed on a hot plate, while the other side compartment was held at the temperature of the glovebox. All compartments were covered with a septum to facilitate stable temperature readings. Each of the side compartments was fitted with a thermocouple (stainless steel) that was placed at the same height as the electrodes. Temperature measurements and associated calibration were carried out as previously described. The side compartment containing the reference/auxiliary electrode was held at the glovebox temperature while the solution in the side compartment that contained the Ag/AgNO_3_ electrode that served as the working electrode was heated in the temperature range ∼20–42 °C. The temperature was increased in ∼2–4 °C intervals and the system was left to equilibrate for ∼10 min under stirring at 800 rpm at each temperature before *E*_OCP_ was recorded for 120 s under continuous stirring at 800 rpm at the given temperature. Average *E*_OCP_ values were plotted against the temperature difference between the two side compartments and the slope of the linear fit to the data provided the temperature coefficient of the Ag/AgNO_3_ reference electrode potential (α_ref_) in given solution conditions. Reported values are an average obtained from 3–4 independent measurements unless otherwise noted. Error bars denote the standard deviation of measurements of independently prepared samples. These data are summarized in Table S6 and representative examples are shown in Figures S5–S8. Note that the temperature coefficients of the reference electrode potential (α_ref_) in MeCN with 100 mM of TBAPF_6_ or KPF_6_ supporting electrolytes were adapted from previous studies as 0.43(6) mV °C^–1^ and 0.48(7) mV °C^–1^, respectively,^29,44,47^ and confirmed independently using the method described above (Figures S9 and S10). We note that no correction for the thermal liquid-junction potential was made because the contribution should be minimal using our experimental setup. Room-temperature liquid-junction potentials were estimated to be <20 mV when different supporting electrolytes and/or solvents than TBAPF_6_ and MeCN were used (vide supra).

## Results & Discussion

### Effect of Supporting Electrolyte Cation and Solvent on Room-Temperature Redox Chemistry

We first sought to examine the changes in the electrochemical behavior of the (PW_12_)^3–^ anion and its reduced analogs in response to varying the supporting electrolyte cation from noncoordinating TBA^+^ to smaller, coordinating alkali metal cations (K^+^, Li^+^). We hypothesized that participation of the alkali cations in electrostatic interactions with reduced states of (PW_12_)^3–^ would lead to ion-pairing behavior of redox events, evident by anodic shifts in reduction potentials. We further expected that the variation in size of the alkali cation would lead to different extents of ion-pairing-induced anodic shifts in reduction potentials. We note that related experiments have been performed previously for MeCN solutions of (PW_12_)^3–^,^34^ albeit not for solutions containing exclusively electrolytes of alkali metal cations. Our motivation for reproducing elements of prior studies lies in the development of a control set of room-temperature electrochemical data for subsequent variable-temperature electrochemical experiments (vide infra) using additional supporting electrolytes, concentrations, and solvents than reported previously.

The cyclic voltammogram (CV) of 1 mM of (TBA)_3_(PW_12_) in MeCN containing 100 mM of TBAPF_6_ as the supporting electrolyte at room temperature (∼19–21 °C) exhibits two quasi-reversible reduction processes within the potential window investigated here (−0.3 V to −1.4 V vs Ag/AgNO_3_), with *E*_1/2_ = −0.58 V and −1.10 V vs Ag/AgNO_3_ (Figure 2 and Table 1). Each reduction corresponds to a formal W-based reduction event (W^VI^/W^V^); however, we note that the added electron is delocalized across the POM.^27,28^ After having confirmed the anticipated electrochemical behavior of this Keggin-type polyoxotungstate in the presence of noncoordinating TBA^+^ countercations, we shifted our focus to investigating the influence of alkali cations on its electrochemical behavior.

**Figure 2:**
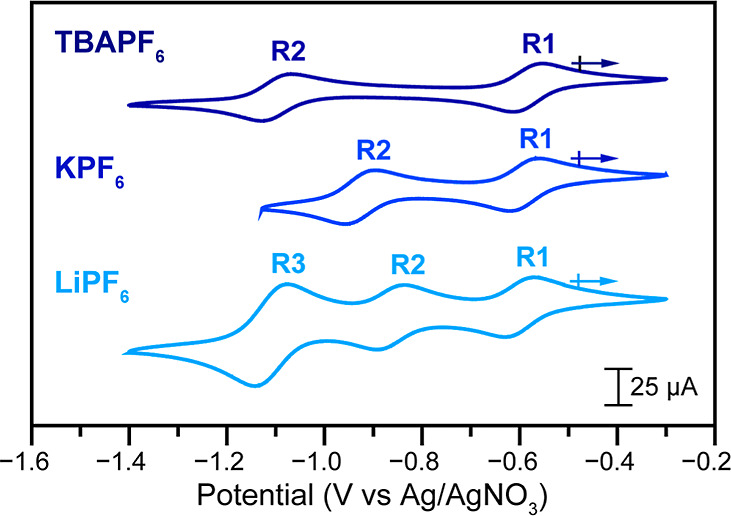
Cyclic voltammograms of 1 mM of (TBA)_3_(PW_12_) in MeCN containing 100 mM of different supporting electrolytes: TBAPF_6_ (top), KPF_6_ (middle), LiPF_6_ (bottom). Measurements were conducted at room temperature (∼19–21 °C) using a scan rate of 100 mV s^–1^. Vertical lines and arrows denote open-circuit potentials and scan directions, respectively.

**Table 1:** Select Electrochemical and Thermodynamic Parameters for POM-Based Redox Couples in MeCN^a^

Supporting Cation	*E*_1/2_ (V vs Ag/AgNO_3_) at 19–21 °C	α^b^ (mV °C^–1^)	Δ*S*_redox_ ^c^ (J K^–1^ mol^–1^)
R1			
TBA^+^	–0.58	–0.59(7)	–57(7)
K^+^	–0.59	–0.56(1)	–54(1)
Li^+^	–0.60	–0.28(1)	–27(1)
R2			
TBA^+^	–1.10	–1.03(9)	–100(9)
K^+^	–0.93	–0.26(1)	–25(1)
Li^+^	–0.86	0.57(1)	55(9)
R3			
Li^+^	–1.11	0.62(6)	120(12)

a The values are obtained from VT-CV analysis of 1 mM solution of (TBA)_3_(PW_12_) in MeCN containing 100 mM of MPF_6_ (M = TBA^+^, K^+^, Li^+^) as the supporting electrolyte using glassy carbon working electrode, Ag/AgNO_3_ reference electrode, and Pt auxiliary electrode at a scan rate of 100 mV s^–1^ in an isothermal single-compartment cell.

b Average values from at least three independent measurements; error bars denote standard deviations of those measurements.

c Calculated using eq 8; errors were estimated using error propagation of the average temperature coefficient values.

Figure 2 displays the CVs of 1 mM of (TBA)_3_(PW_12_) in MeCN in the presence of 100 mM of alkali hexafluorophosphate salts (MPF_6_; M = K^+^, Li^+^) at room temperature (∼19–21 °C). In the presence of KPF_6_, the CV exhibits two quasi-reversible reduction processes with *E*_1/2_ = −0.59 V and −0.93 V vs Ag/AgNO_3_. The (PW_12_)^3–^/(PW_12_)^4–^ (R1) reduction possesses a half-wave potential that is nearly identical to the value observed in the presence of the noncoordinating TBA^+^ cation. However, the second reduction, formally corresponding to the (PW_12_)^4–^/(PW_12_)^5–^ (R2) couple, is anodically shifted by 170 mV as compared to the corresponding value measured in a solution containing 100 mM of TBAPF_6_. These data suggest that the surface of (PW_12_)^5–^ is sufficiently basic to drive alkali ion coordination, translating to a potassium-coupled reduction process. We note that these results are generally consistent with prior observations made by our group, where the basicity of the surface of (PW_12_)^*n*−^ increases as additional electrons are added to the assembly.^29^

The CV of 1 mM of (TBA)_3_(PW_12_) in MeCN with 100 mM of LiPF_6_ as the supporting electrolyte shows significantly different electrochemical behavior. Three quasi-reversible reduction events are observed with *E*_1/2_ = −0.60 V, −0.86 V, and −1.11 V vs Ag/AgNO_3_. The *E*_1/2_ value of the first reduction event ((PW_12_)^3–^/(PW_12_)^4–^; R1) resembles the values observed for (TBA)_3_(PW_12_) in the presence of TBAPF_6_ and KPF_6_ supporting electrolytes, providing additional support for the conclusion that limited cation association occurs with the −3 and −4 charge states of the polyoxotungstate. The second reduction event (formally (PW_12_)^4–^/(PW_12_)^5–^; R2) is shifted anodically by 240 mV as compared to the corresponding value measured in the presence of 100 mM of TBAPF_6_. We postulate that the larger shift observed in the presence of LiPF_6_ than KPF_6_ originates from tighter ion pairing between (PW_12_)^5–^ and Li^+^ as compared to (PW_12_)^5–^ and K^+^.^61^ This observation aligns with previous reports that found ion association constants between (PW_12_)^5–^ and Li^+^ to be 1–2 orders of magnitude larger than between (PW_12_)^5–^ and Na^+^.^34^ Indeed, square-wave voltammetry (SWV) data collected for MeCN solutions containing 1 mM of (TBA)_3_(PW_12_), 100 mM of TBAPF_6_, and 100 mM of MPF_6_ (M = K^+^, Li^+^) show analogous behavior in *E*_1/2_ values as observed in the CV measurements (Figures S11 and S12). Fits of the data obtained in the presence of 0–100 mM of MPF_6_ (M = K^+^, Li^+^) to a Langmuir isotherm afford effective ion association constants of *K*_a_ = 54(1) M^–1^ and *K*_a_ = 212(12) M^–1^ for the formally (PW_12_)^4–^/(PW_12_)^5–^ (R2) couple in the presence of K^+^ and Li^+^, respectively (Figures S13 and S14). Given the small anodic shifts in *E*_1/2_ for the (PW_12_)^3–^/(PW_12_)^4–^ (R1) couple in the presence of K^+^ and Li^+^, these results suggest that ion pairing between (PW_12_)^5–^ and Li^+^ is ∼4× stronger than between (PW_12_)^5–^ and K^+^ in MeCN.

Another key difference between the CV and SWV data collected in the presence of LiPF_6_ and the analogous data with KPF_6_ and/or TBAPF_6_ is the observation of an additional reduction event near the cathodic end of the potential window investigated. The increase in current response as compared to the first two reduction events signifies that this redox process corresponds to a 2-electron reduction event (formally (PW_12_)^5–^/(PW_12_)^7–^; R3), which was confirmed by peak integrations of SWV data (Figure S15). The large anodic shift of this reduction event from the third reduction in the presence of TBAPF_6_ (Figures S1 and 2) suggests that the reduction process is lithium-coupled.^34^ Scheme 1 summarizes the redox reactions of (PW_12_)^*n*−^ redox species in the potential window investigated in the presence of different supporting electrolyte cations.

**Scheme 1:**
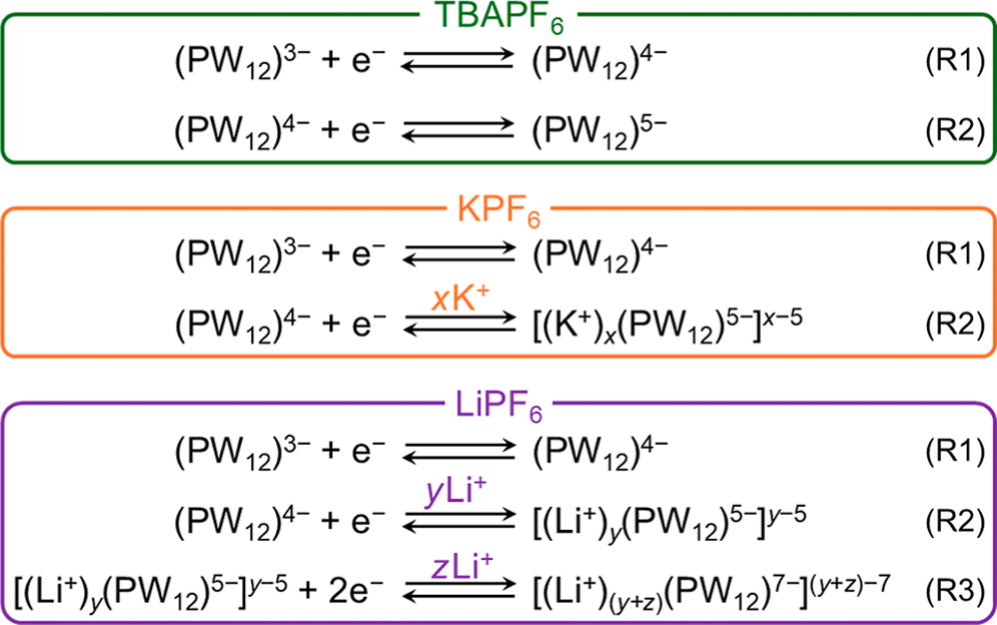
Summary of Redox Reactions of (PW_12_)^*n*−^ Redox Species in the Potential Window Investigated in the Presence of Different Supporting Electrolyte Cations^a^

a The number of associating alkali cations is represented by *x*, *y*, and *z*, where *x*, *y*, and *z* = 1–3.^34^

Having established that the polyoxotungstate exhibits cation-dependent electrochemical behavior in MeCN, we next examined how changing the solvent to DMF alters its redox behavior in the presence of the same supporting electrolyte salts. In contrast to MeCN, DMF introduces a more viscous, basic environment that is expected to modify both the solvation of the alkali cations and their association with the anionic cluster.^42,62–66^ As shown in Figure 3, the change in solvent from MeCN to DMF leads to systematic shifts of the POM-based redox waves to more negative potentials (Tables 1 and 2), and a decrease in current response.

**Figure 3:**
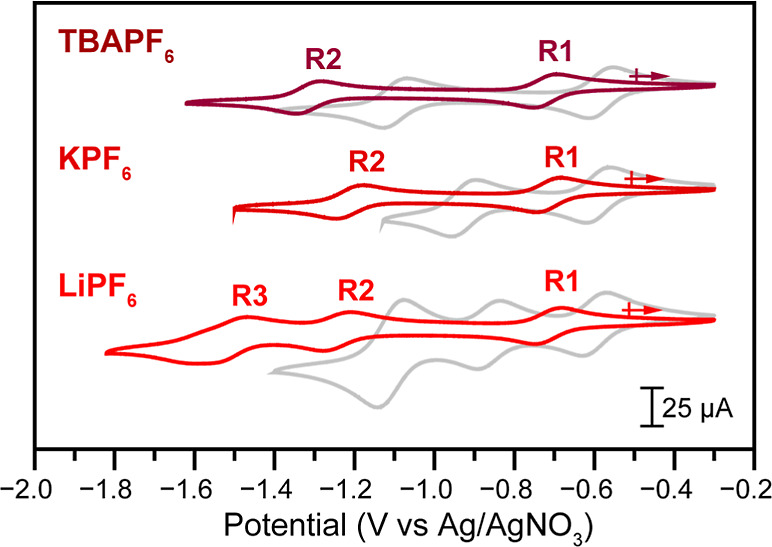
Cyclic voltammograms of 1 mM of (TBA)_3_(PW_12_) in DMF containing 100 mM of different supporting electrolytes (different shades of red): TBAPF_6_ (top), KPF_6_ (middle), LiPF_6_ (bottom), compared with analogous voltammograms collected in MeCN (gray). Measurements were conducted at room temperature (∼19–21 °C) using a scan rate of 100 mV s^–1^. Vertical lines and arrows denote open-circuit potentials and scan directions, respectively.

**Table 2:** Select Electrochemical and Thermodynamic Parameters for POM-Based Redox Couples in DMF^a^

Supporting Cation	*E*_1/2_ (V vs Ag/AgNO_3_) at 19–21 °C	α^b^ (mV °C^–1^)	Δ*S*_redox_ ^c^ (J K^–1^ mol^–1^)
R1			
TBA^+^	–0.72	–0.71(2)	–68(2)
K^+^	–0.71	–0.80(3)	–78(3)
Li^+^	–0.71	–0.47(5)	–46(5)
R2			
TBA^+^	–1.32	–1.00(11)	–96(10)
K^+^	–1.21	–0.11(1)	–11(1)
Li^+^	–1.24	0.21(4)	20(4)
R3			
Li^+^	–1.52	0.58(9)	112(18)

a The values are obtained from VT-CV analysis of 1 mM solution of (TBA)_3_(PW_12_) in DMF containing 100 mM of MPF_6_ (M = TBA^+^, K^+^, Li^+^) as the supporting electrolyte using glassy carbon working electrode, Ag/AgNO_3_ reference electrode, and Pt auxiliary electrode at a scan rate of 100 mV s^–1^ in an isothermal single-compartment cell.

b Average values from at least three independent measurements; error bars denote standard deviations of those measurements.

c Calculated using eq 8; errors were estimated using error propagation of the average temperature coefficient values.

The first reduction event ((PW_12_)^3–^/(PW_12_)^4–^; R1) exhibits *E*_1/2_ = −0.71 V to −0.72 V vs Ag/AgNO_3_ in DMF across all supporting electrolytes, representing a negative shift of 110–140 mV from the values obtained in MeCN. The shift is even more pronounced for the second and third reduction events, with the *E*_1/2_ values for the formally (PW_12_)^4–^/(PW_12_)^5–^ (R2) couple cathodically shifted by 220 mV, 280 mV, and 380 mV in TBAPF_6_, KPF_6_, and LiPF_6_ electrolytes, respectively, and the *E*_1/2_ value for the formally (PW_12_)^5–^/(PW_12_)^7–^ (R3) couple shifted by 410 mV in LiPF_6_ (Figure 3). Similar solvent-dependent shifts in half-wave potentials have been observed previously for the first and second reductions of the α- and β-isomers of (PW_12_)^3–^ in solutions containing 100 mM of TBAPF_6_ supporting electrolyte,^33^ and are attributed to the higher Gutmann acceptor number for MeCN as compared to DMF (18.9 for MeCN; 16.0 for DMF), which is a measure of solvent’s Lewis acidity.^64^ Thus, MeCN accepts more electron density than DMF from POM anions, decreasing the energy required for reduction. Peak integrations of SWV data indicate that the third reduction of (TBA)_3_(PW_12_) is a 2-electron process in DMF as was observed in MeCN (Figure S16).

The fact that DMF has a significantly higher Gutmann donor number than MeCN (26.6 for DMF; 14.1 for MeCN), which is a measure of solvent’s Lewis basicity,^64^ indicates that DMF can more efficiently solvate cations of the supporting electrolyte.^62,67,68^ Stronger cation solvation by DMF results in weaker direct interactions between polyoxotungstate anions and supporting electrolyte cations in this solvent in comparison to MeCN. Accordingly, ion-pairing interactions between the reduced charge states of the POM (formally (PW_12_)^5–^ and (PW_12_)^7–^) and alkali cations are significantly different in MeCN and DMF. In MeCN, cation–POM interactions are likely contact ion pairs while solvent-shared ion pairs are formed in DMF.^69–75^ This is supported by the lower sensitivity of the cation–POM ion-pairing interaction to the charge density of the alkali ion in DMF. To illustrate, in DMF, the half-wave potential for the formally (PW_12_)^4–^/(PW_12_)^5–^ (R2) couple is anodically shifted by 80 mV and 110 mV in the presence of 100 mM of Li^+^ and K^+^, respectively, as compared to the value measured in a solution containing 100 mM of TBAPF_6_ (Figure 3 and Table 2), but analogous anodic shifts of 240 mV and 170 mV were observed in MeCN (Figure 2 and Table 1). This observation is further supported by SWV analysis for DMF solutions containing 1 mM of (TBA)_3_(PW_12_), 100 mM of TBAPF_6_, and 0–100 mM of MPF_6_ (M = K^+^, Li^+^) (Figures S17 and S18). In the presence of 100 mM of MPF_6_ (M = K^+^, Li^+^), similar shifts in *E*_1/2_ are observed as in the CV study. Fits of the SWV data collected in the presence of 0–100 mM of MPF_6_ (M = K^+^, Li^+^) to a Langmuir isotherm afford effective ion association constants of *K*_a_ = 11(2) M^–1^ and *K*_a_ = 20(12) M^–1^ for the formally (PW_12_)^4–^/(PW_12_)^5–^ (R2) couple in the presence of K^+^ and Li^+^, respectively (Figures S19 and S20). The similar values of *K*_a_ for K^+^ and Li^+^ in DMF, as opposed to the ∼4× difference observed in MeCN, indicate that the nature of ion-pairing interactions in the two solvents are different. Moreover, the ∼5× and ∼10× lower values of *K*_a_ for K^+^ and Li^+^ in DMF, respectively, as compared to the corresponding values in MeCN, reflect weaker ion-pairing interactions between (PW_12_)^5–^ and M^+^ (M = K^+^, Li^+^) in DMF than in MeCN. Together, these results indicate that while DMF and MeCN exhibit similar dielectric constants and polarities,^63,65,76,77^ the extent and structure of cation–POM ion-pairing interactions are greatly affected by the Lewis basicity of the solvent.

Figure 3 shows that for the same concentration of the (TBA)_3_(PW_12_) analyte, the peak current in DMF is lower than in MeCN under identical experimental conditions. Moreover, the peak separation (Δ*E*_p_) for the redox couples increases more with increasing scan rate in DMF than in MeCN (Figures S21-S23). These trends point toward slower apparent electron-transfer kinetics in DMF. To quantify the electrokinetic behavior of the polyoxotungstate in MeCN and DMF in the presence of different supporting electrolyte cations, we first evaluated the diffusion coefficients (*D*_0_) of the (PW_12_)^*n*−^ redox species via Randles–Ševčík analysis (Figures S24–S51 and Tables S2–S5). As expected from the room-temperature cyclic voltammetry data, the diffusion coefficients of all (PW_12_)^*n*−^ redox species are smaller in DMF across all supporting electrolytes. We attribute this solvent-dependent difference in diffusion coefficients to the higher viscosity of DMF as compared to MeCN (0.92 cP for DMF at 20 °C; 0.36 cP for MeCN at 20 °C).^65,78–80^ Additionally, the observed *D*_0_ values are statistically similar for the R1 and R2 redox couples in each solvent, regardless of the supporting electrolyte identity. These results suggest that the hydrodynamic radii of cation–POM ion pairs in both solvents are dominated by the larger POM anion and any difference in ion pairing is too small to significantly change the effective size of the diffusing species.^34,81^ We note that the *D*_0_ values for the R3 redox couple in the presence of LiPF_6_ are significantly lower than those observed for the R1 and R2 couples, indicating that differences in ion pairing and/or solvation for the formally –7 charge state of the POM result in a slight increase in the effective hydrodynamic radius.^15^

Using the cathodic diffusion coefficients, we then estimated the heterogeneous electron-transfer rate constants (*k*_0_) of (PW_12_)^3–^ reductions using the Nicholson method (Figures S52–S58), which necessitate the evaluation of peak potential separation as a function of scan rate. In MeCN, both R1 and R2 redox couples show small peak separations up to 10 V s^–1^, indicating that fast, nearly Nernstian, outer-sphere electron transfer that is not sensitive to the identity of the supporting electrolyte cation is operative (Figures S21–S23).^74,82–84^ In contrast, the rate constants reveal the impact of supporting electrolyte identity in DMF (Table S7). For the first reduction (R1), *k*_0_ is independent of cation identity, consistent with a largely outer-sphere process in which cation–POM interactions play an insignificant role.^83,84^ However, *k*_0_ for the second reduction (R2) is over 2-fold lower in the presence of K^+^ and Li^+^ than with TBA^+^, indicating that the formally (PW_12_)^4–^/(PW_12_)^5–^ reduction is kinetically inhibited as a result of ion-pairing interactions in DMF.

The above observations suggest that half-wave potentials and electron-transfer kinetics are affected by the presence and nature of ion-pairing interactions, which are in turn influenced by the choice of solvent. To probe how (dis)­order within the cation–POM–solvent ensemble evolves upon reduction and to extract the underlying entropic contributions in electron-transfer reactions, we directed our focus toward temperature-dependent electrochemical measurements.

### Effect of Supporting Electrolyte Cation and Solvent on Entropy of Reduction Reactions

In an effort to probe the influence of ion-pairing interactions and solvation on the change in entropy associated with electron-transfer reactions of POMs, we conducted variable-temperature cyclic voltammetry (VT-CV) experiments of (PW_12_)^3–^ in the presence of TBAPF_6_, KPF_6_, and LiPF_6_ supporting electrolytes in both MeCN and DMF. The data collected in MeCN in the temperature range 20–39 °C under isothermal conditions are displayed in Figure 4. For each redox event, a plot of *E*_1/2_ against temperature was created to obtain the temperature coefficient (α) of a given reduction event as the slope of the linear fit to the data, after correcting for the temperature dependence of the Ag/AgNO_3_ reference electrode potential (Figures S59–S65).^29,41,46,47^ The temperature coefficient values are linearly correlated with the redox entropy through eq 8. Importantly, we emphasize that these quantities of Δ*S*_redox_ represent changes in entropy between charge states of the polyoxotungstate, not the absolute entropy of a given solution state.

**Figure 4:**
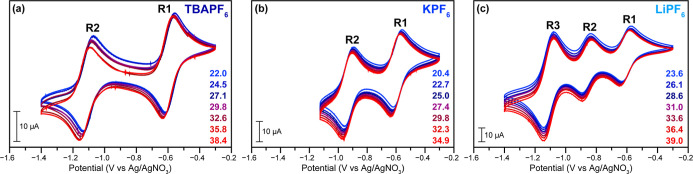
Cyclic voltammograms of 1 mM of (TBA)_3_(PW_12_) in MeCN containing 100 mM of different supporting electrolytes: (a) TBAPF_6_, (b) KPF_6_, (c) LiPF_6_; collected at variable temperatures (∼20–39 °C) using 100 mV s^–1^ scan rate in an isothermal single-compartment cell.

Prior reports from our group gave values of Δ*S*_redox_ = −53(11) J K^–1^ mol^–1^ and −88(4) J K^–1^ mol^–1^ for the first (R1) and second (R2) reduction of (PW_12_)^3–^ in the presence of TBAPF_6_ supporting electrolyte (Scheme 1) in MeCN.^29^ Here, these data were reproduced to give statistically identical values of Δ*S*_redox_ = −57(7) J K^–1^ mol^–1^ and −100(9) J K^–1^ mol^–1^ for R1 and R2, respectively (Figure 4a and Table 1).

While a similar value of Δ*S*_redox_ = −54(1) J K^–1^ mol^–1^ is observed for the (PW_12_)^3–^/(PW_12_)^4–^ (R1) reduction in the presence of KPF_6_ electrolyte, the value for the second reduction (R2) is significantly less negative (Δ*S*_redox_ = −25(1) J K^–1^ mol^–1^) than that measured in TBAPF_6_ electrolyte solution (Figure 4b and Table 1). These results agree with the room-temperature CV and SWV data that suggest lack of alkali cation interaction with (PW_12_)^3–^ and (PW_12_)^4–^, but significant ion pairing with the −5 charge state, (PW_12_)^5–^. Previous work from us and others has shown that the redox entropy of polyoxotungstate anions in the presence of noncoordinating countercations becomes increasingly negative as the charges and charge state changes upon reduction become more negative,^29,85^ following the electrostatic Born model of ion solvation.^41,86,87^ In electrolyte solutions containing coordinating cations, such as alkali metal ions, solvation of cation–POM ion pairs must also be considered. We attribute the increase (shift to a less negative value) in Δ*S*_redox_ for the second reduction of (PW_12_)^3–^ as compared to the first reduction in KPF_6_ electrolyte solution to charge compensation of the reduced charge state due to K^+^-coupled electron transfer (Scheme 1) and disruption of solvation shell around the cluster anion upon K^+^ binding. To illustrate, if the formally (PW_12_)^4–^/(PW_12_)^5–^ (R2) reduction is coupled with the transfer of one K^+^ ion, the ion-paired assembly [(K^+^)­(PW_12_)^5–^]^4–^ is generated and the net change in overall charge is 0. In comparison, the change in overall cluster charge is −1 for the cation-uncoupled (PW_12_)^3–^/(PW_12_)^4–^ (R1) reduction (Scheme 1). Thus, from an electrostatic perspective, the difference in solvent ordering around [(K^+^)­(PW_12_)^5–^]^4–^ compared to (PW_12_)^4–^ is less than between (PW_12_)^4–^ and (PW_12_)^3–^, with the reduced charge states exhibiting larger degree of solvent ordering for both redox couples.

In the presence of LiPF_6_ supporting electrolyte, the redox entropy for the (PW_12_)^3–^/(PW_12_)^4–^ (R1) reduction of Δ*S*_redox_ = −27(1) J K^–1^ mol^–1^ is less negative than the values obtained in the TBAPF_6_ and KPF_6_ electrolyte solutions (Table 1). While this result may be surprising considering the lack of significant interactions between Li^+^ and both (PW_12_)^3–^ and (PW_12_)^4–^, we postulate that this observation may arise from interference from Li^+^ solvation that causes less ordered solvent molecules near the surface of the reduced polyoxotungstate and overall decrease in order of the reduction reaction.^88^ On the other hand, the redox entropies for the second and third reductions of (PW_12_)^3–^ (R2 and R3) in the presence of Li^+^ are *positive* (Δ*S*_redox_ = 55(9) J K^–1^ mol^–1^ for R2; Δ*S*_redox_ = 120(12) J K^–1^ mol^–1^ for R3). We ascribe these observations to similar ion-pairing behavior and disruption of cluster solvation shell as observed in the presence of K^+^, albeit involving stronger cation–POM interactions, as indicated by larger *K*_a_ value, and possibly greater number of alkali cations. Previous studies report (PW_12_)^5–^ to interact with 2–3 Li^+^ ions,^34^ resulting in [(Li^+^)_2_(PW_12_)^5–^]^3–^ and/or [(Li^+^)_3_(PW_12_)^5–^]^2–^ ion-paired assemblies. This translates to an overall *decrease* in anionic charge of the assembly following the Li^+^-coupled reduction reaction. As such, a positive Δ*S*_redox_ would be anticipated because of less ordered solvent around the resultant ion pairs i.e., [(Li^+^)_2_(PW_12_)^5–^]^3–^ and/or [(Li^+^)_3_(PW_12_)^5–^]^2–^ in comparison to (PW_12_)^4–^.^37,41,60,89^ Comparison of the VT-CV data for the R2 redox couple of the POM across MeCN solutions containing different supporting electrolyte cations shows that one can move from a redox system with large negative redox entropy (net order) to a system with positive redox entropy (net disorder) by simply changing the countercation in solution (Table 1).

Upon switching solvents from MeCN to DMF (Figures S66–S75), similar trends in changes of redox entropy in the presence of different supporting electrolyte cations are observed (Table 2). The redox entropy for the (PW_12_)^3–^/(PW_12_)^4–^ (R1) reduction is similar in the presence of TBA^+^ and K^+^ (Δ*S*_redox_ = −68(2) J K^–1^ mol^–1^ for TBA^+^; Δ*S*_redox_ = −78(3) J K^–1^ mol^–1^ for K^+^), but significantly less negative with Li^+^ (Δ*S*_redox_ = −46(5) J K^–1^ mol^–1^), attributed to strong solvation of Li^+^ in DMF as in MeCN.^66,68,75,90,91^ The redox entropy for the second reduction of (PW_12_)^3–^ (R2) increased from Δ*S*_redox_ = −96(10) J K^–1^ mol^–1^ in the presence of TBA^+^ to Δ*S*_redox_ = −11(1) J K^–1^ mol^–1^ and 20(4) J K^–1^ mol^–1^ in the presence of K^+^ and Li^+^, respectively, ascribed to ion-pairing interactions and associated compensation of anionic charge and disruption of solvation shell around the polyoxotungstate anion upon alkali ion binding as observed in MeCN. The smaller difference in values of Δ*S*_redox_ for the R2 redox couple between DMF solutions containing K^+^ and Li^+^ than corresponding MeCN solutions aligns well with the smaller difference in effective ion association constants in DMF. Similarly, the over 2-fold lower redox entropy value for the R2 redox couple in the presence of LiPF_6_ in DMF than in MeCN may be ascribed to an order of magnitude smaller effective ion association constant. The increase in disorder is more pronounced for the third reduction of (PW_12_)^3–^ (R3) in LiPF_6_ electrolyte solution, providing a statistically identical value to that observed in MeCN.

Taken together, VT-CV analysis of (PW_12_)^3–^ provides valuable insights into how ion pairing and solvation affect the thermodynamics of electron transfer under different solution conditions. While values of Δ*S*_redox_ follow similar qualitative patterns for a given redox couple across MeCN and DMF, their signs and magnitude depend strongly on the supporting electrolyte cation, highlighting the impact of cation–POM interactions. The ability to switch Δ*S*_redox_ from negative to positive values by simply changing the identity of the supporting electrolyte cation (i.e., TBA^+^ to Li^+^) suggests a practical handle for tuning the entropic component of redox behavior in POMs, with implications for designing systems where controlled redox-induced ordering or disordering is beneficial, such as for redox mediators, electrocatalysts, and redox electrolytes for thermoelectrochemical devices.

## Conclusions

The results presented in this work demonstrate that varying the supporting electrolyte cation from noncoordinating tetrabutylammonium to coordinating alkali metal ions and employing solvents with different Lewis basicity affects the kinetic and thermodynamic properties of the Keggin-type polyoxometalate anion (PW_12_)^3–^. Our analyses show that solvent and supporting electrolyte cation provide independent but coupled handles for tuning the electrochemical behavior of the POM. In MeCN, electrochemical reductions of (PW_12_)^3–^ remain relatively fast with minimal scan-rate dependence of Δ*E*_p_, indicating that electron transfer is largely outer-sphere and not significantly perturbed by cation identity on the CV time scale. However, in DMF, heterogeneous electron-transfer rate constants span several orders of magnitude, with the second and third reduction of (PW_12_)^3–^ becoming markedly slower in the presence of Li^+^ and K^+^ relative to TBA^+^. Variable-temperature CV analyses reveal that reductions of (PW_12_)^3–^ in the presence of TBA^+^ yield negative Δ*S*_redox_ values, corroborating an increase in order upon electron uptake. In contrast, the second reduction of (PW_12_)^3–^ in the presence of K^+^ or Li^+^ is accompanied by a decrease in order as compared to the first reduction or net disorder (i.e., positive Δ*S*_redox_ values), respectively. The variation in Δ*S*_redox_ in the presence of different alkali cations is greater in MeCN than in DMF owing to stronger ion-pairing interactions between alkali cations and (PW_12_)^5–^ in MeCN. Overall, these results underscore the importance of considering ion-pairing and solvation interactions when tailoring the kinetics and thermodynamics of electron transfer of redox-active molecules for targeted applications in electrochemistry. Future work will extend this platform to other POM families and redox-active coordination compounds to investigate how broadly ion-pairing and solvation interactions may modulate electrochemical kinetics and redox entropies for the design of redox mediators, electrocatalysts, redox electrolytes, and electrochemical sensors.

## Supplementary Material



## Notes

Additional electrochemical data, including diffusion coefficients, reference electrode calibration data, SWV data, variable-scan-rate CV data, Randles–Ševčík plots, Nicholson plots, and variable-temperature CV data (PDF)
