# Supplementary material for: Effects of Supporting Electrolyte and Solvent on the Thermodynamics and Kinetics of Reduction Reactions in a Keggin-Type Polyoxotungstate
Source: Inorg Chem. 2026 Jun 4;65(24):13441–52. doi: 10.1021/acs.inorgchem.6c01354 (PMC13292200; doi:10.1021/acs.inorgchem.6c01354)
Supplement: Supplementary file 1 [file ic6c01354_si_001.pdf]

Supporting Information for:

Effects of Supporting Electrolyte and Solvent on the Thermodynamics and Kinetics of Reduction Reactions in a Keggin-Type Polyoxotungstate

Hania A. Guirguis, Mamta Dagar, Sophia M. Anderson, Ellen M. Matson, and Agnes E. Thorarinsdottir**

Department of Chemistry, University of Rochester, Rochester NY 14627, USA

*Correspondence to: matson@chem.rochester.edu (E.M.M.);
agnes.thorarinsdottir@rochester.edu (A.E.T)

A. Supplementary Tables

Table S1. $E_{1/2}$ values of ferrocene for liquid-junction potential estimation S4
Table S2. Summary of anodic diffusion coefficients in MeCN S5
Table S3. Summary of cathodic diffusion coefficients in MeCN S6
Table S4. Summary of anodic diffusion coefficients in DMF S7
Table S5. Summary of cathodic diffusion coefficients in DMF.......................................S8
Table S6. Temperature coefficients of reference electrode potential.............................S9
Table S7. Electron-transfer rate constants for reductions of $(TBA)_3(PW_{12})$ in DMF S10

B. Supplementary Figures

Figure S1. CV of $(TBA)_3(PW_{12})$ in MeCN with $TBAPF_6$..S11
Figure S2. CV of $(TBA)_3(PW_{12})$ in MeCN with KPF_6..S12
Figure S3. SWV of $(TBA)_3(PW_{12})$ in MeCN with variable amounts of $TBAPF_6$ S25
Figure S4. SWV of $(TBA)_3(PW_{12})$ in DMF with variable amounts of $TBAPF_6$..............S14
Figure S5. VT-OCP analysis of reference electrode in MeCN with $LiPF_6$ S15
Figure S6. VT-OCP analysis of reference electrode in DMF with $TBAPF_6$ S16
Figure S7. VT-OCP analysis of reference electrode in DMF with KPF_6......................S17
Figure S8. VT-OCP analysis of reference electrode in DMF with $LiPF_6$ S18
Figure S9. VT-OCP analysis of reference electrode in MeCN with $TBAPF_6$...............S19
Figure S10. VT-OCP analysis of reference electrode in MeCN with KPF_6 ....S20
Figure S11. SWV of $(TBA)_3(PW_{12})$ in MeCN with variable amounts of KPF_6..............S21
Figure S12. SWV of $(TBA)_3(PW_{12})$ in MeCN with variable amounts of $LiPF_6$ S22
Figure S13. Plot of $\Delta E_{1/2}$ vs $[KPF_6]$ for R2 couple in MeCN.......................................S23
Figure S14. Plot of $\Delta E_{1/2}$ vs $[LiPF_6]$ for R2 couple in MeCN S24

Figure S15. SWV of $(TBA)_3(PW_{12})$ and peak integrations in MeCN with $LiPF_6$ S25
Figure S16. SWV of $(TBA)_3(PW_{12})$ and peak integrations in DMF with $LiPF_6$ S26
Figure S17. SWV of $(TBA)_3(PW_{12})$ in DMF with variable amounts of KPF_6 .... S25
Figure S18. SWV of $(TBA)_3(PW_{12})$ in DMF with variable amounts of $LiPF_6$ S28
Figure S19. Plot of $\Delta E_{1/2}$ vs [KPF_6] for R2 couple in DMF S29
Figure S20. Plot of $\Delta E_{1/2}$ vs [$LiPF_6$] for R2 couple in DMF S30
Figure S21. ΔE_p vs scan rate of R1–R2 couples in MeCN and DMF with $TBAPF_6$ S31
Figure S22. ΔE_p vs scan rate of R1–R2 couples in MeCN and DMF with KPF_6 .... S32
Figure S23. ΔE_p vs scan rate of R1–R3 couples in MeCN and DMF with $LiPF_6$ S33
Figure S24. Variable-scan-rate CVs of R1 couple in MeCN with $TBAPF_6$ S34
Figure S25. Variable-scan-rate CVs of R2 couple in MeCN with $TBAPF_6$ S35
Figure S26. Randles–Ševčík plots of R1 couple in MeCN with $TBAPF_6$ S36
Figure S27. Randles–Ševčík plots of R2 couple in MeCN with $TBAPF_6$ S37
Figure S28. Variable-scan-rate CVs of R1 couple in MeCN with KPF_6 .... S38
Figure S29. Variable-scan-rate CVs of R2 couple in MeCN with KPF_6 .... S39
Figure S30. Randles–Ševčík plots of R1 couple in MeCN with KPF_6 .... S40
Figure S31. Randles–Ševčík plots of R2 couple in MeCN with KPF_6 .... S41
Figure S32. Variable-scan-rate CVs of R1 couple in MeCN with $LiPF_6$ S42
Figure S33. Variable-scan-rate CVs of R2 couple in MeCN with $LiPF_6$ S43
Figure S34. Variable-scan-rate CVs of R3 couple in MeCN with $LiPF_6$ S44
Figure S35. Randles–Ševčík plots of R1 couple in MeCN with $LiPF_6$ S45
Figure S36. Randles–Ševčík plots of R2 couple in MeCN with $LiPF_6$ S46
Figure S37. Randles–Ševčík plots of R3 couple in MeCN with $LiPF_6$ S47
Figure S38. Variable-scan-rate CVs of R1 couple in DMF with $TBAPF_6$ S48
Figure S39. Variable-scan-rate CVs of R2 couple in DMF with $TBAPF_6$ S49
Figure S40. Randles–Ševčík plots of R1 couple in DMF with $TBAPF_6$ S50
Figure S41. Randles–Ševčík plots of R2 couple in DMF with $TBAPF_6$ S51
Figure S42. Variable-scan-rate CVs of R1 couple in DMF with KPF_6 .... S52
Figure S43. Variable-scan-rate CVs of R2 couple in DMF with KPF_6 .... S53
Figure S44. Randles–Ševčík plots of R1 couple in DMF with KPF_6 .... S54
Figure S45. Randles–Ševčík plots of R2 couple in DMF with KPF_6 .... S55
Figure S46. Variable-scan-rate CVs of R1 couple in DMF with $LiPF_6$ S56
Figure S47. Variable-scan-rate CVs of R2 couple in DMF with $LiPF_6$ S57
Figure S48. Variable-scan-rate CVs of R3 couple in DMF with $LiPF_6$ S58
Figure S49. Randles–Ševčík plots of R1 couple in DMF with $LiPF_6$ S59
Figure S50. Randles–Ševčík plots of R2 couple in DMF with $LiPF_6$ S60
Figure S51. Randles–Ševčík plots of R3 couple in DMF with $LiPF_6$ S61
Figure S52. Nicholson plot of R1 couple in DMF with $TBAPF_6$ S62
Figure S53. Nicholson plot of R2 couple in DMF with $TBAPF_6$ S63
Figure S54. Nicholson plot of R1 couple in DMF with KPF_6 .... S64

Figure S55. Nicholson plot of R2 couple in DMF with KPF_6 .... S65
Figure S56. Nicholson plot of R1 couple in DMF with $LiPF_6$ S66
Figure S57. Nicholson plot of R2 couple in DMF with $LiPF_6$ S67
Figure S58. Nicholson plot of R3 couple in DMF with $LiPF_6$ S68
Figure S59. VT-CV analysis of R1 couple in MeCN with $TBAPF_6$ S69
Figure S60. VT-CV analysis of R2 couple in MeCN with $TBAPF_6$ S70
Figure S61. VT-CV analysis of R1 couple in MeCN with KPF_6 .... S71
Figure S62. VT-CV analysis of R2 couple in MeCN with KPF_6 .... S72
Figure S63. VT-CV analysis of R1 couple in MeCN with $LiPF_6$ S73
Figure S64. VT-CV analysis of R2 couple in MeCN with $LiPF_6$ S74
Figure S65. VT-CV analysis of R3 couple in MeCN with $LiPF_6$ S75
Figure S66. VT-CVs of $(TBA)_3(PW_{12})$ in DMF with $TBAPF_6$ S76
Figure S67. VT-CVs of $(TBA)_3(PW_{12})$ in DMF with KPF_6 .... S77
Figure S68. VT-CVs of $(TBA)_3(PW_{12})$ in DMF with $LiPF_6$ S78
Figure S69. VT-CV analysis of R1 couple in DMF with $TBAPF_6$ S79
Figure S70. VT-CV analysis of R2 couple in DMF with $TBAPF_6$ S80
Figure S71. VT-CV analysis of R1 couple in DMF with KPF_6 .... S81
Figure S72. VT-CV analysis of R2 couple in DMF with KPF_6 .... S82
Figure S73. VT-CV analysis of R1 couple in DMF with $LiPF_6$ S83
Figure S74. VT-CV analysis of R2 couple in DMF with $LiPF_6$ S84
Figure S75. VT-CV analysis of R3 couple in DMF with $LiPF_6$ S85

C. References...S86

A. Supplementary Tables

Table S1. $E_{1/2}$ values of 1 mM of ferrocene (Fc) in different solvents and in the presence of 100 mM of different supporting electrolytes to establish an upper limit for the liquid-junction potential at the Ag/AgNO$_3$ reference electrode.

Supporting Electrolyte	Solvent	$E_{1/2}$ of $Fc^{+/0}$ (V vs Ag/AgNO$_3$)
$TBAPF_6$	MeCN	0.090
KPF_6	MeCN	0.082
$LiPF_6$	MeCN	0.085
$TBAPF_6$	DMF	0.072
KPF_6	DMF	0.070
$LiPF_6$	DMF	0.071

Table S2. Summary of anodic diffusion coefficients (D_0) for the redox couples of $(TBA)_3(PW_{12})$ in MeCN containing 100 mM of different supporting electrolytes, as calculated from Randles-Ševčík analysis (eqs 2 and 3) at 20 °C via variable-scan-rate CV measurements.[1,2]

Supporting Electrolyte	Redox Couple	Reversible D_0[a] ($cm^2\ s^{-1}$)	Irreversible D_0[b] ($cm^2\ s^{-1}$)
$TBAPF_6$	R1	6.0(6) × 10^{-6}	1.0(1) × 10^{-5}
	R2	5(2) × 10^{-6}	9(3) × 10^{-6}
KPF_6	R1	7(1) × 10^{-6}	1.2(2) × 10^{-5}
	R2	1.1(3) × 10^{-5}	1.7(5) × 10^{-5}
$LiPF_6$	R1	6(1) × 10^{-6}	1.0(2) × 10^{-5}
	R2	3.7(5) × 10^{-6}	6.0(9) × 10^{-6}
	R3[c]	1.5(3) × 10^{-6}	2(1) × 10^{-6}

[a]D_0 values estimated using the reversible form of the Randles–Ševčík equation (eq 2). The errors denote the standard deviation obtained from three independent measurements of variable-scan-rate CVs.

[b]D_0 values estimated using the irreversible form of the Randles–Ševčík equation (eq 3). The errors denote the standard deviation obtained from three independent measurements of variable-scan-rate CVs.

[c]All calculations for the R3 redox couple in the presence of $LiPF_6$ considered the number of electrons, *n*, involved in the redox process as 2 due to it being a two-electron redox process, as confirmed by integrations of areas under the curves of the corresponding SWV data (Figure S15).

Table S3. Summary of cathodic diffusion coefficients (D_0) for the redox couples of $(TBA)_3(PW_{12})$ in MeCN containing 100 mM of different supporting electrolytes, as calculated from Randles-Ševčík analysis (eqs 2 and 3) at 20 °C via variable-scan-rate CV measurements.[1,2]

Supporting Electrolyte	Redox Couple	Reversible D_0[a] ($cm^2\ s^{-1}$)	Irreversible D_0[b] ($cm^2\ s^{-1}$)
$TBAPF_6$	R1	$1.0(2) \times 10^{-5}$	$1.7(4) \times 10^{-5}$
	R2	$1.0(1) \times 10^{-5}$	$1.6(2) \times 10^{-5}$
KPF_6	R1	$1.1(2) \times 10^{-5}$	$1.8(3) \times 10^{-5}$
	R2	$1.6(3) \times 10^{-5}$	$2.7(5) \times 10^{-5}$
$LiPF_6$	R1	$1.0(2) \times 10^{-5}$	$1.6(4) \times 10^{-5}$
	R2	$1.1(2) \times 10^{-5}$	$1.8(3) \times 10^{-5}$
	R3[c]	$3.3(4) \times 10^{-6}$	$2.7(4) \times 10^{-6}$

[a]D_0 values estimated using the reversible form of the Randles–Ševčík equation (eq 2). The errors denote the standard deviation obtained from three independent measurements of variable-scan-rate CVs.

[b]D_0 values estimated using the irreversible form of the Randles–Ševčík equation (eq 3). The errors denote the standard deviation obtained from three independent measurements of variable-scan-rate CVs.

[c]All calculations for the R3 redox couple in the presence of $LiPF_6$ considered the number of electrons, *n*, involved in the redox process as 2 due to it being a two-electron redox process, as confirmed by integrations of areas under the curves of the corresponding SWV data (Figure S15).

Table S4. Summary of anodic diffusion coefficients (D_0) for the redox couples of $(TBA)_3(PW_{12})$ in DMF containing 100 mM of different supporting electrolytes, as calculated from Randles-Ševčík analysis (eqs 2 and 3) at 20 °C via variable-scan-rate CV measurements.[1,2]

Supporting Electrolyte	Redox Couple	Reversible D_0[a] ($cm^2\ s^{-1}$)	Irreversible D_0[b] ($cm^2\ s^{-1}$)
$TBAPF_6$	R1	3.9(8) × 10^{-6}	6(1) × 10^{-6}
	R2	3.5(6) × 10^{-6}	6(1) × 10^{-6}
KPF_6	R1	3.9(4) × 10^{-6}	6.4(7) × 10^{-6}
	R2	3.7(6) × 10^{-6}	6(1) × 10^{-6}
$LiPF_6$	R1	3.7(2) × 10^{-6}	5.9(1) × 10^{-6}
	R2	2.6(3) × 10^{-6}	4.2(6) × 10^{-6}
	R3[c]	4(2) × 10^{-7}	3(1) × 10^{-7}

[a]D_0 values estimated using the reversible form of the Randles–Ševčík equation (eq 2). The errors denote the standard deviation obtained from three independent measurements of variable-scan-rate CVs.

[b]D_0 values estimated using the irreversible form of the Randles–Ševčík equation (eq 3). The errors denote the standard deviation obtained from three independent measurements of variable-scan-rate CVs.

[c]All calculations for the R3 redox couple in the presence of $LiPF_6$ considered the number of electrons, *n*, involved in the redox process as 2 due to it being a two-electron redox process, as confirmed by integrations of areas under the curves of the corresponding SWV data (Figure S16).

Table S5. Summary of cathodic diffusion coefficients (D_0) for the redox couples of $(TBA)_3(PW_{12})$ in DMF containing 100 mM of different supporting electrolytes, as calculated from Randles-Ševčík analysis (eqs 2 and 3) at 20 °C via variable-scan-rate CV measurements.[1,2]

Supporting Electrolyte	Redox Couple	Reversible D_0[a] (cm^2 s^{-1})	Irreversible D_0[b] (cm^2 s^{-1})
$TBAPF_6$	R1	5.2(1) × 10^{-6}	8.4(2) × 10^{-6}
	R2	4.9(6) × 10^{-6}	8(1) × 10^{-6}
KPF_6	R1	4.9(2) × 10^{-6}	7.9(3) × 10^{-6}
	R2	5(2) × 10^{-6}	9(2) × 10^{-6}
$LiPF_6$	R1	4.6(4) × 10^{-6}	7.4(7) × 10^{-6}
	R2	4.9(7) × 10^{-6}	8(1) × 10^{-6}
	R3[c]	8(1) × 10^{-7}	6.1(9) × 10^{-7}

[a]D_0 values estimated using the reversible form of the Randles–Ševčík equation (eq 2). The errors denote the standard deviation obtained from three independent measurements of variable-scan-rate CVs.

[b]D_0 values estimated using the irreversible form of the Randles–Ševčík equation (eq 3). The errors denote the standard deviation obtained from three independent measurements of variable-scan-rate CVs.

[c]All calculations for the R3 redox couple in the presence of $LiPF_6$ considered the number of electrons, *n*, involved in the redox process as 2 due to it being a two-electron redox process, as confirmed by integrations of areas under the curves of the corresponding SWV data (Figure S16).

Table S6. Summary of temperature coefficients of the $Ag/AgNO_3$ reference electrode potential (α_{ref}) in the solution conditions used in this study.

**Supporting Electrolyte**[a]	**α_{ref} (mV °C^{-1})**
**MeCN**	
$TBAPF_6$	0.43(6)[5]
KPF_6	0.48(7)[6]
$LiPF_6$	0.56(7)[b]
**DMF**	
$TBAPF_6$	0.66(8)[b]
KPF_6	0.56(8)[b]
$LiPF_6$	0.78(3)[b]

[a]A concentration of 100 mM of supporting electrolyte was used.

[b]Reported values are averages obtained from three independent measurements of non-isothermal variable-temperature open-circuit potential measurements. The errors denote the standard deviation obtained from these measurements.

Table S7. Summary of electron-transfer rate constants (k_0) for the reductions of $(TBA)_3(PW_{12})$ in DMF containing 100 mM of different supporting electrolytes, as calculated from Nicholson analysis (eqs 4 and 5) at 20 °C via variable-scan-rate CV measurements.[3,4]

Supporting Electrolyte	Redox Couple	Reversible k_0[a] ($cm\ s^{-1}$)	Irreversible k_0[b] ($cm\ s^{-1}$)	Average k_0[c] ($cm\ s^{-1}$)
$TBAPF_6$	R1	0.14(1)	0.18(2)	0.16(2)
	R2	0.18(9)	0.22(11)	0.20(8)
KPF_6	R1	0.17(2)	0.22(3)	0.20(2)
	R2	0.04(1)	0.06(1)	0.05(1)
$LiPF_6$	R1	0.18(3)	0.23(4)	0.21(3)
	R2	0.05(2)	0.07(3)	0.06(2)
	R3[d]	0.00013(8)	0.00012(8)	0.00013(6)

[a]k_0 values estimated using reversible cathodic diffusion coefficients obtained using eq 2 (Table S5) and calculated using the Nicholson method (eqs 4 and 5). The errors denote the standard deviation obtained from three independent measurements of variable-scan-rate CVs.

[b]k_0 values estimated using irreversible cathodic diffusion coefficients obtained using eq 3 (Table S5) and calculated using the Nicholson method (eqs 4 and 5). The errors denote the standard deviation obtained from three independent measurements of variable-scan-rate CVs.

[c]k_0 values estimated by taking the average of the reversible and irreversible cathodic diffusion coefficients (Table S5) and calculated using the Nicholson method (eqs 4 and 5). Error bars were obtained from error propagation.

[d]All calculations for the R3 redox couple in the presence of $LiPF_6$ considered the number of electrons, n, involved in the redox process as 2 due to it being a two-electron redox process, as confirmed by integrations of areas under the curves of the corresponding SWV data (Figure S16).

B. Supplementary Figures

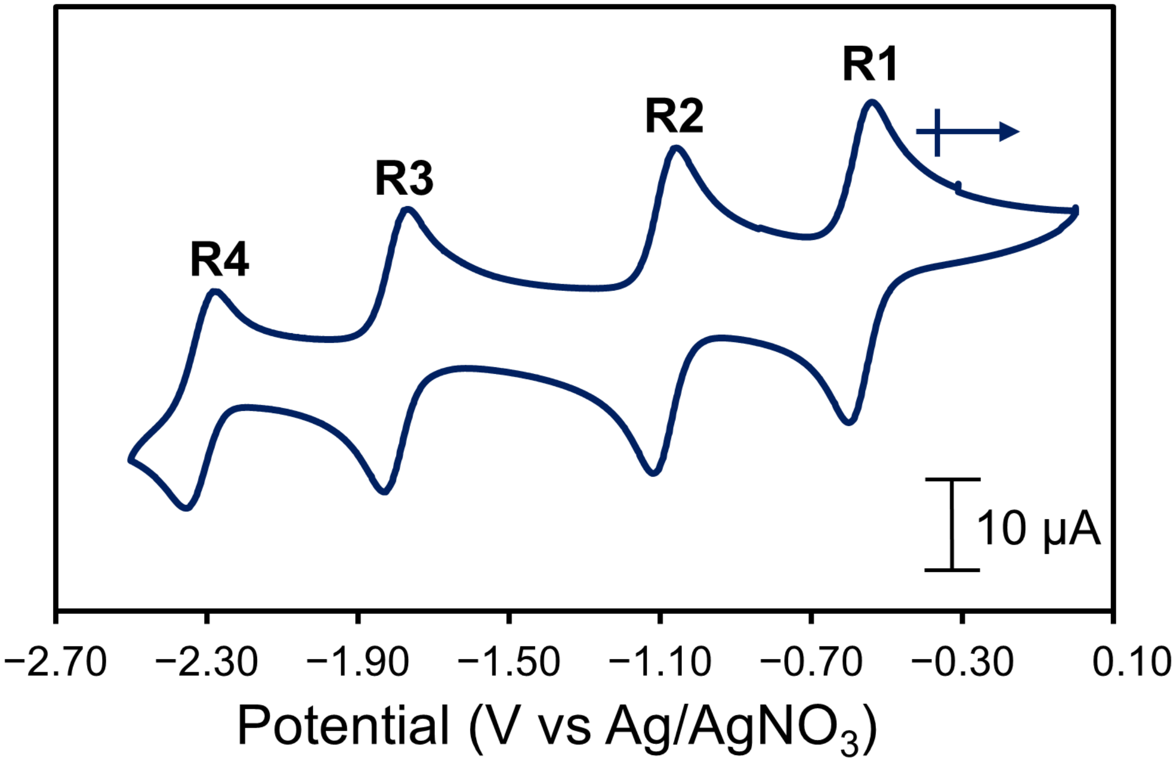

Figure S1. CV of 1 mM of $(TBA)_3(PW_{12})$ in MeCN containing 100 mM of $TBAPF_6$ supporting electrolyte collected at room temperature (~19–21 °C) using 100 mV s^{-1} scan rate in a wider potential window (0 V to −2.5 V vs $Ag/AgNO_3$). Vertical blue line and arrow denote the open-circuit potential and scan direction, respectively.

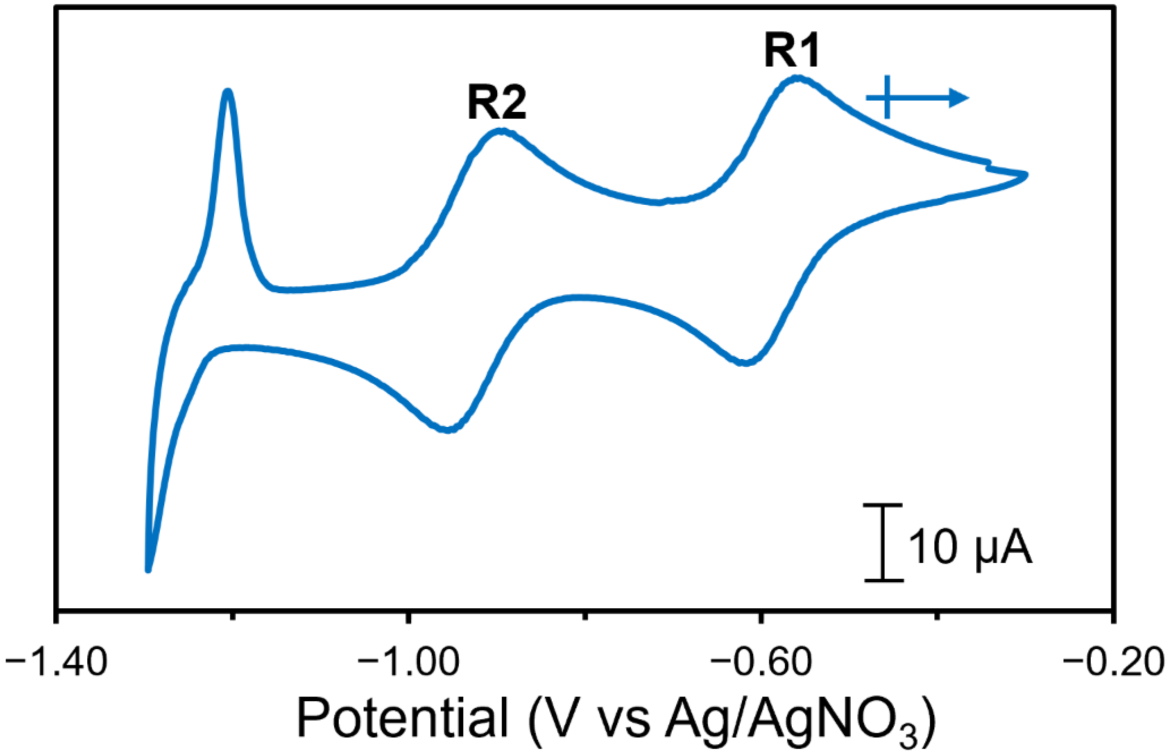

Figure S2. CV of 1 mM of $(TBA)_3(PW_{12})$ in MeCN containing 100 mM of KPF_6 supporting electrolyte collected at room temperature (~19–21 °C) using 100 mV s^{-1} scan rate in a wider potential window (−0.3 V to −1.35 V vs $Ag/AgNO_3$). Vertical blue line and arrow denote the open-circuit potential and scan direction, respectively.

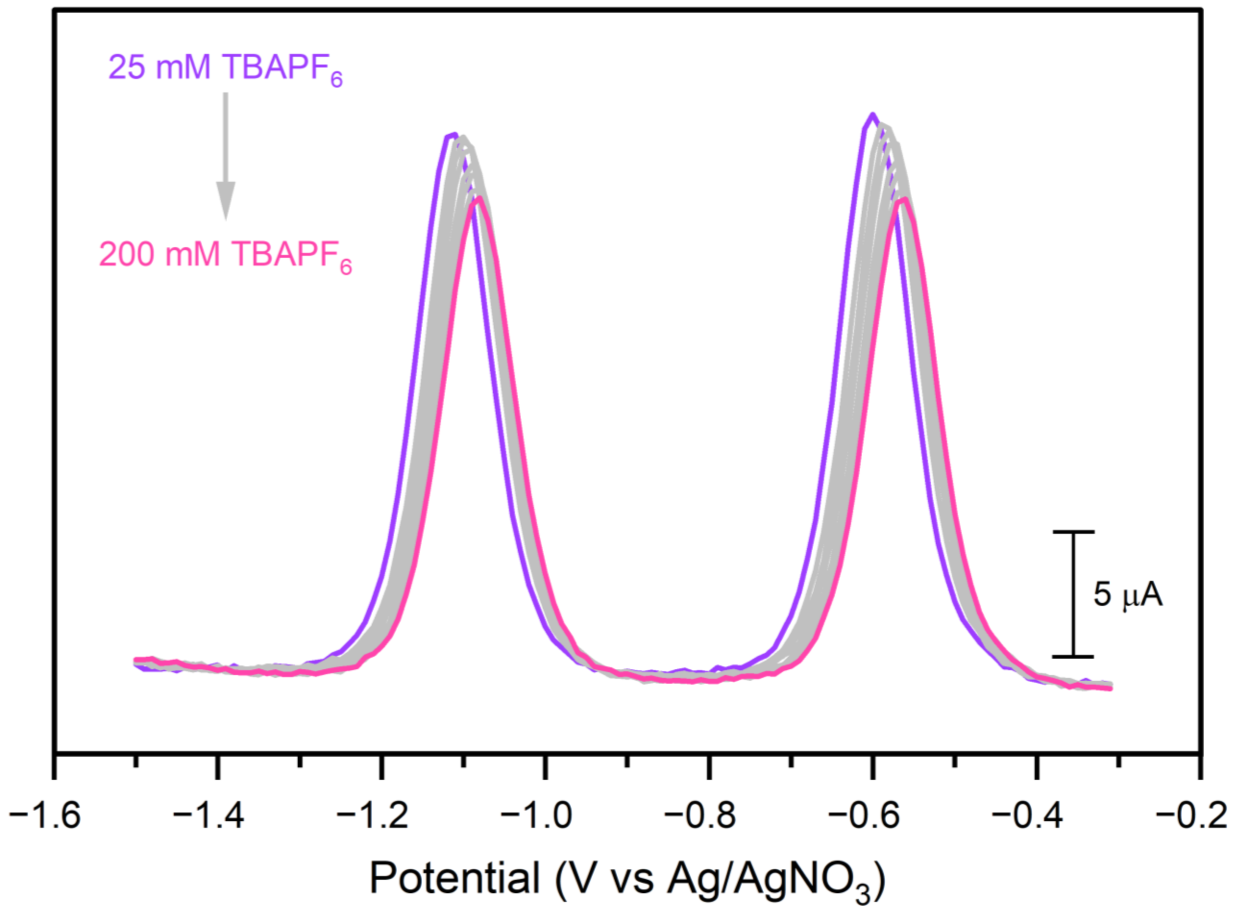

Figure S3. SWV of 1 mM of $(TBA)_3(PW_{12})$ in MeCN containing 25–200 mM of $TBAPF_6$ supporting electrolyte collected at room temperature (~19–21 °C) using a pulse height of 25 mV, pulse width of 100 ms, and step height of 10 mV.

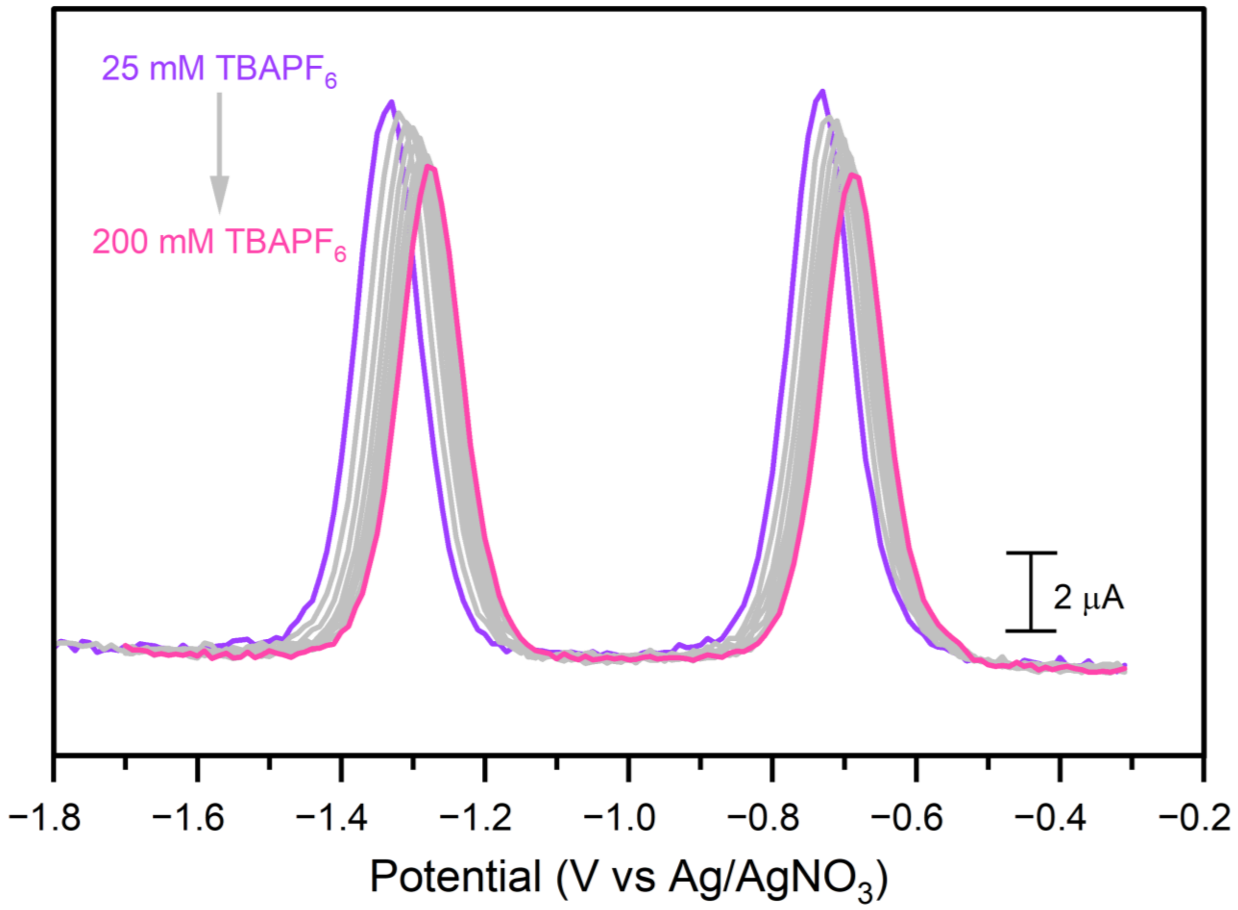

Figure S4. SWV of 1 mM of $(TBA)_3(PW_{12})$ in DMF containing 25–200 mM of $TBAPF_6$ supporting electrolyte collected at room temperature (~19–21 °C) using a pulse height of 25 mV, pulse width of 100 ms, and step height of 10 mV.

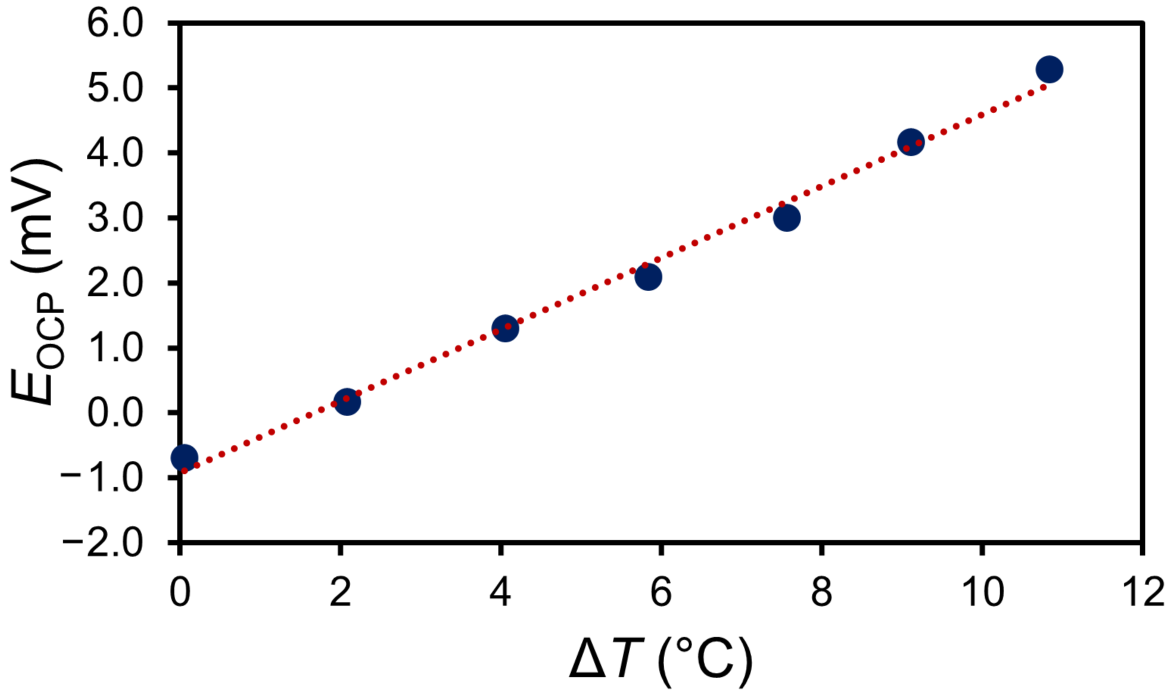

Figure S5. Plot of the potential difference between two Ag/$AgNO_3$ reference electrodes at open-circuit conditions vs temperature difference collected in non-isothermal setup. Blue circles denote experimental data, and the dotted red line corresponds to a linear fit to the data. The slope of the linear fit to the data represents the temperature coefficient, in mV °C^{-1}, of the Ag/$AgNO_3$ reference electrode potential in MeCN containing 100 mM of $LiPF_6$ supporting electrolyte. An average value of 0.56(7) mV °C^{-1} was obtained (Table S6). The error in the average value corresponds to the standard deviation of three independent measurements.

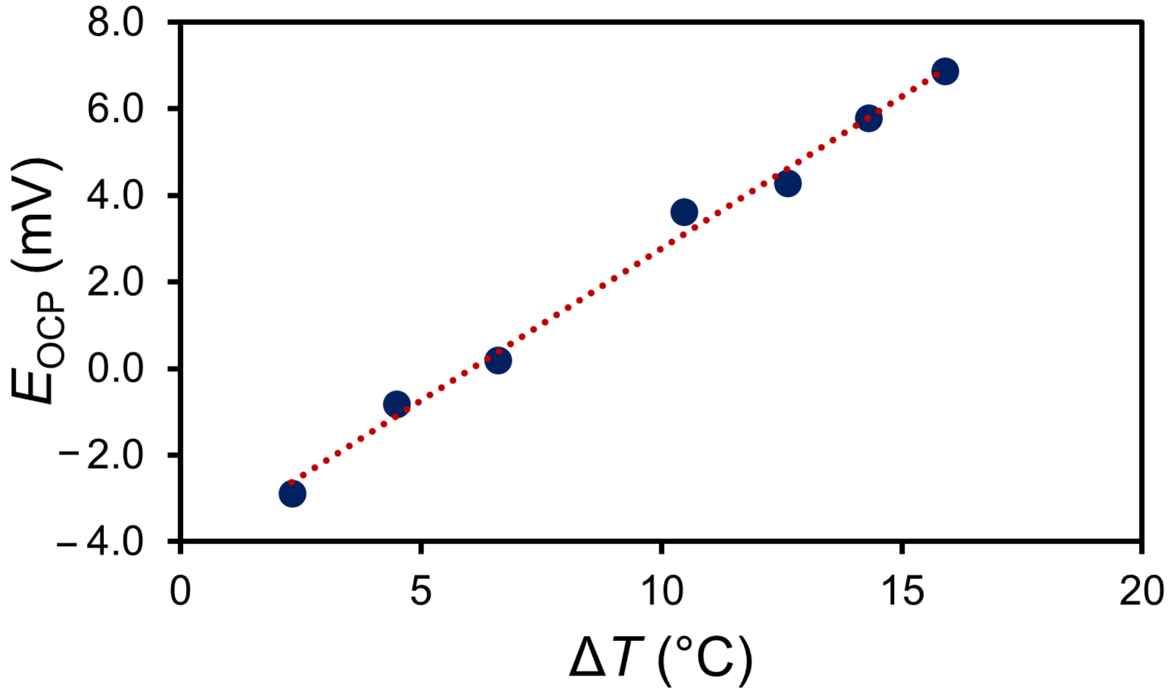

Figure S6. Plot of the potential difference between two Ag/$AgNO_3$ reference electrodes at open-circuit conditions vs temperature difference collected in non-isothermal setup. Blue circles denote experimental data, and the dotted red line corresponds to a linear fit to the data. The slope of the linear fit to the data represents the temperature coefficient, in mV °C^{-1}, of the Ag/$AgNO_3$ reference electrode potential in DMF containing 100 mM of $TBAPF_6$ supporting electrolyte. An average value of 0.66(8) mV °C^{-1} was obtained (Table S6). The error in the average value corresponds to the standard deviation of three independent measurements.

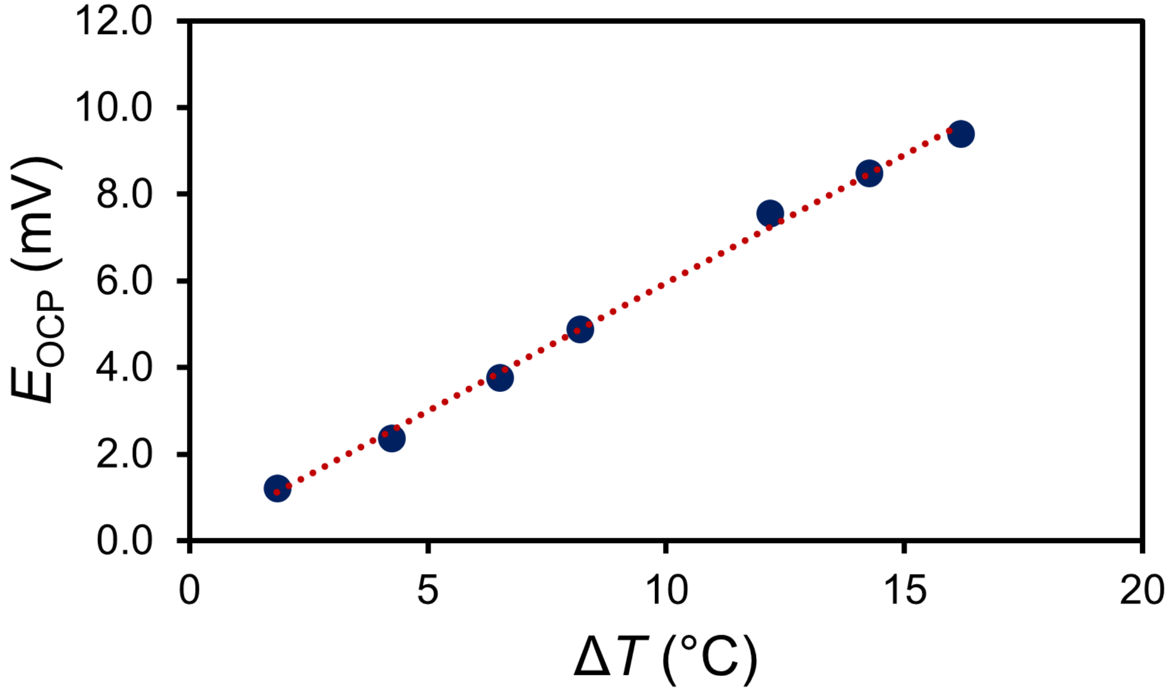

Figure S7. Plot of the potential difference between two Ag/$AgNO_3$ reference electrodes at open-circuit conditions vs temperature difference collected in non-isothermal setup. Blue circles denote experimental data, and the dotted red line corresponds to a linear fit to the data. The slope of the linear fit to the data represents the temperature coefficient, in mV °C^{-1}, of the Ag/$AgNO_3$ reference electrode potential in DMF containing 100 mM of KPF_6 supporting electrolyte. An average value of 0.56(8) mV °C^{-1} was obtained (Table S6). The error in the average value corresponds to the standard deviation of three independent measurements.

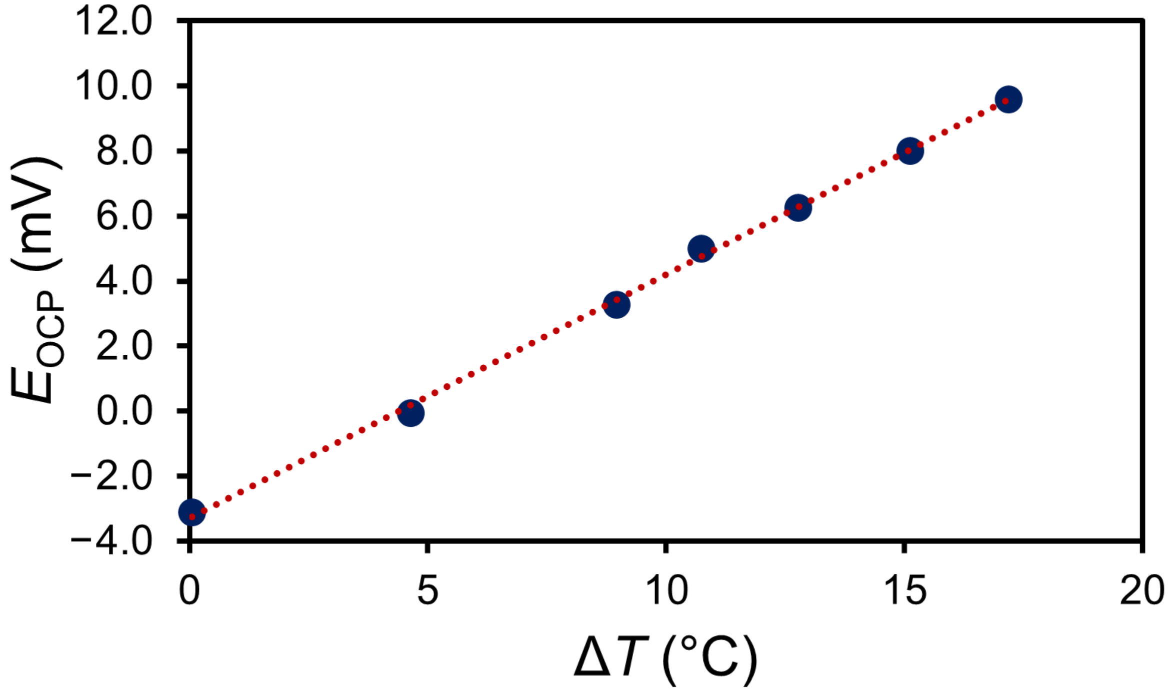

Figure S8. Plot of the potential difference between two $Ag/AgNO_3$ reference electrodes at open-circuit conditions vs temperature difference collected in non-isothermal setup. Blue circles denote experimental data, and the dotted red line corresponds to a linear fit to the data. The slope of the linear fit to the data represents the temperature coefficient, in mV $°C^{-1}$, of the $Ag/AgNO_3$ reference electrode potential in DMF containing 100 mM of $LiPF_6$ supporting electrolyte. An average value of 0.78(3) mV $°C^{-1}$ was obtained (Table S6). The error in the average value corresponds to the standard deviation of three independent measurements.

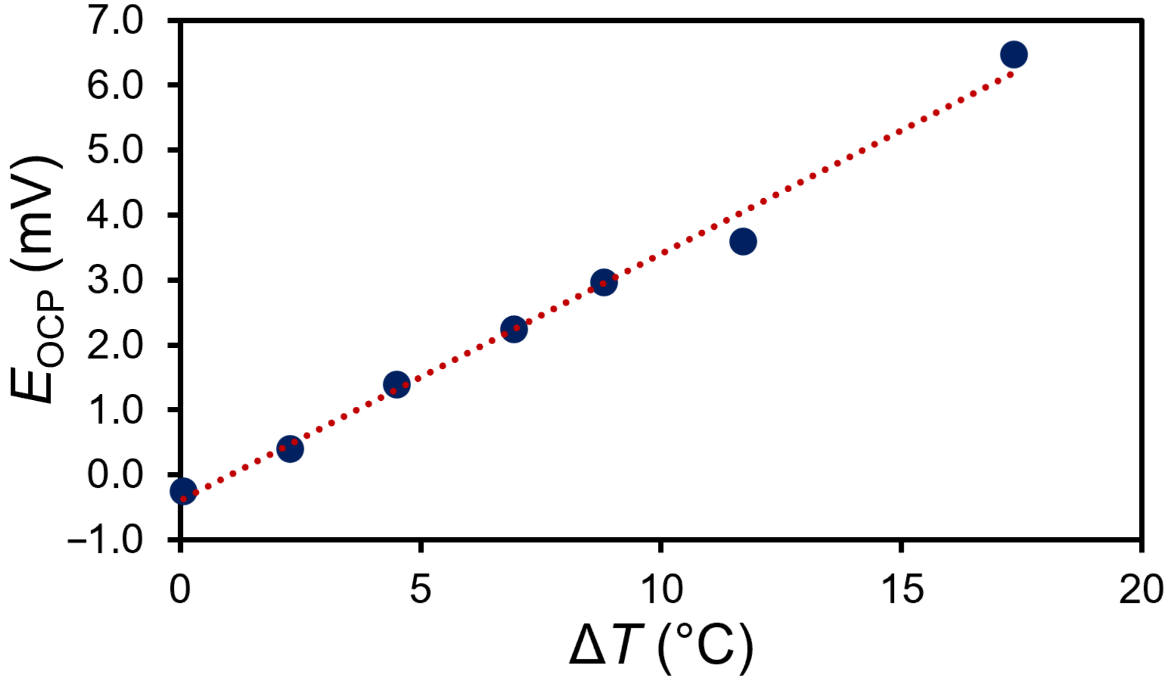

Figure S9. Plot of the potential difference between two Ag/$AgNO_3$ reference electrodes at open-circuit conditions vs temperature difference collected in non-isothermal setup. Blue circles denote experimental data, and the dotted red line corresponds to a linear fit to the data. The slope of the linear fit to the data represents the temperature coefficient, in mV °C^{-1}, of the Ag/$AgNO_3$ reference electrode potential in MeCN containing 100 mM of $TBAPF_6$ supporting electrolyte. A value of 0.38(2) mV °C^{-1} was obtained (Table S6), where the error denotes the standard deviation of the linear fit to the data.

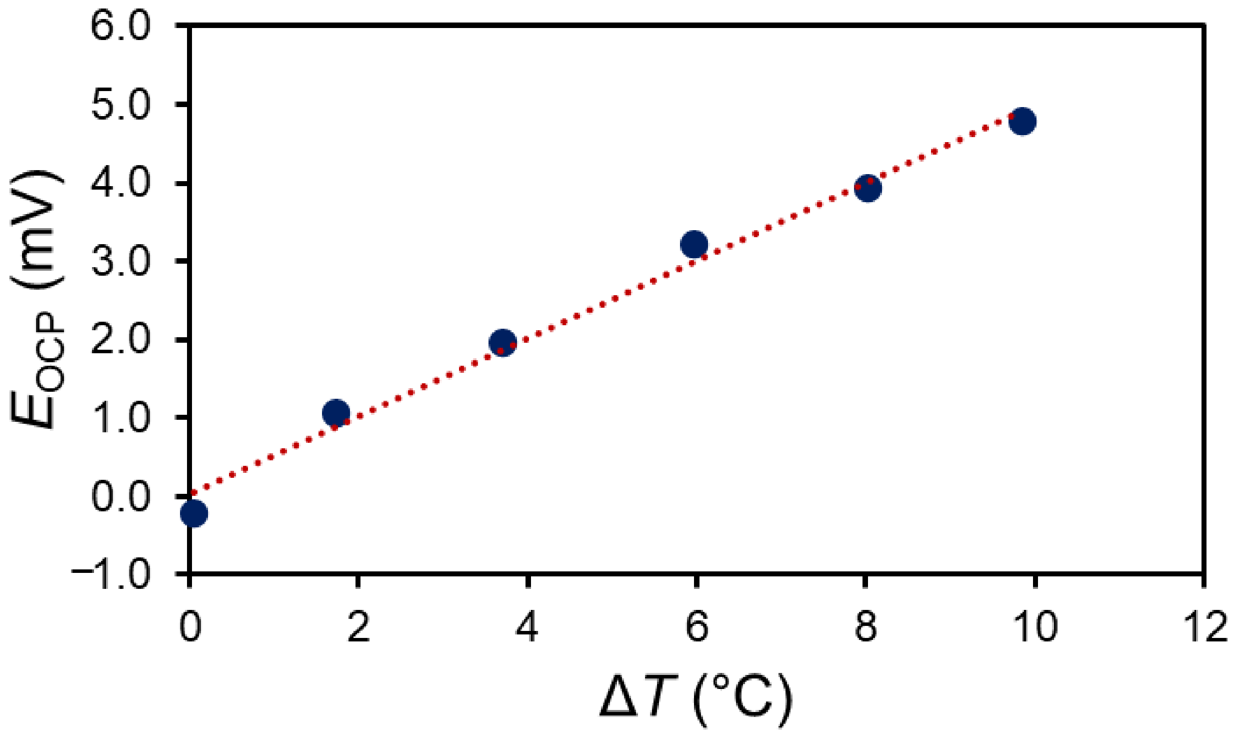

Figure S10. Plot of the potential difference between two Ag/$AgNO_3$ reference electrodes at open-circuit conditions vs temperature difference collected in non-isothermal setup. Blue circles denote experimental data, and the dotted red line corresponds to a linear fit to the data. The slope of the linear fit to the data represents the temperature coefficient, in mV $°C^{-1}$, of the Ag/$AgNO_3$ reference electrode potential in MeCN containing 100 mM of KPF_6 supporting electrolyte. A value of 0.49(2) mV $°C^{-1}$ was obtained (Table S6), where the error denotes the standard deviation of the linear fit to the data.

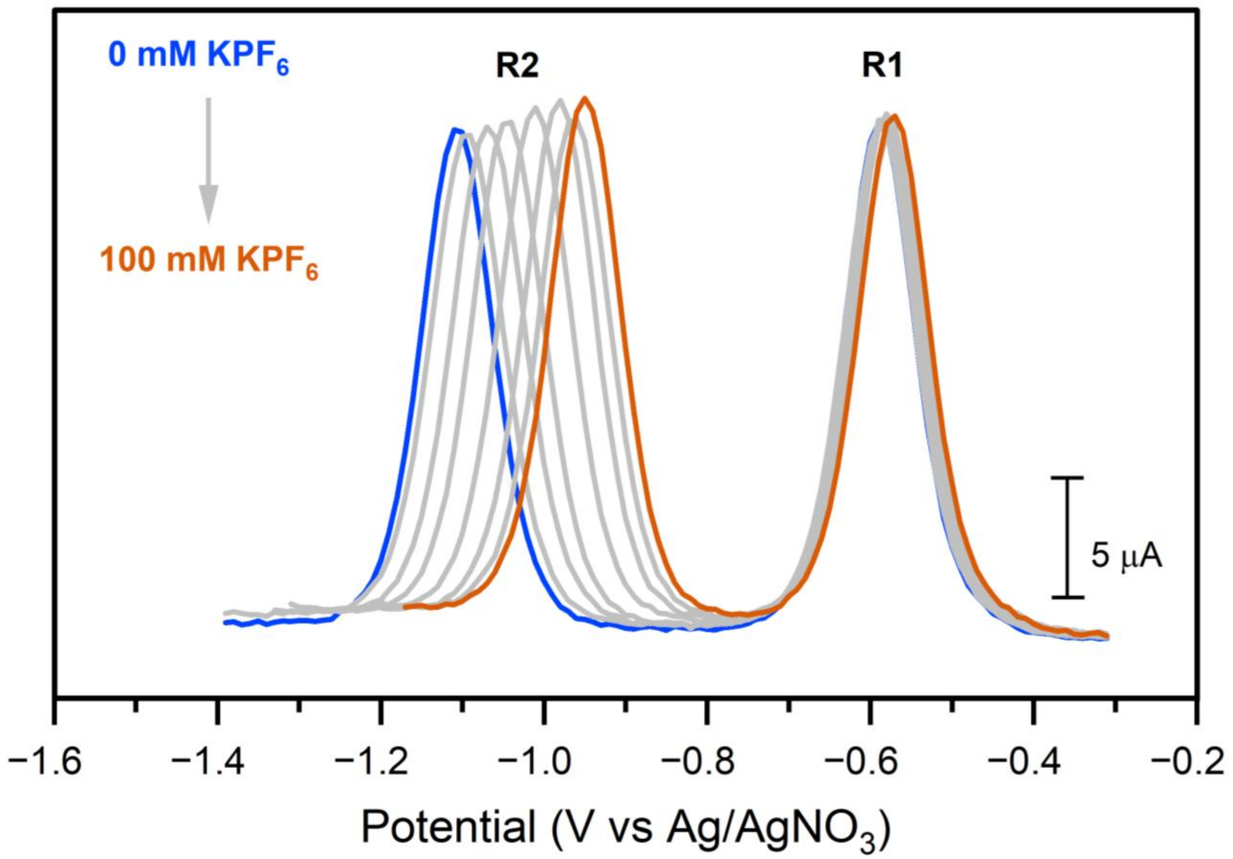

Figure S11. SWV of 1 mM of $(TBA)_3(PW_{12})$ in MeCN containing 100 mM of $TBAPF_6$ and variable amounts of KPF_6 (0–100 mM) supporting electrolytes collected at room temperature (~19–21 °C) using a pulse height of 25 mV, pulse width of 100 ms, and step height of 10 mV.

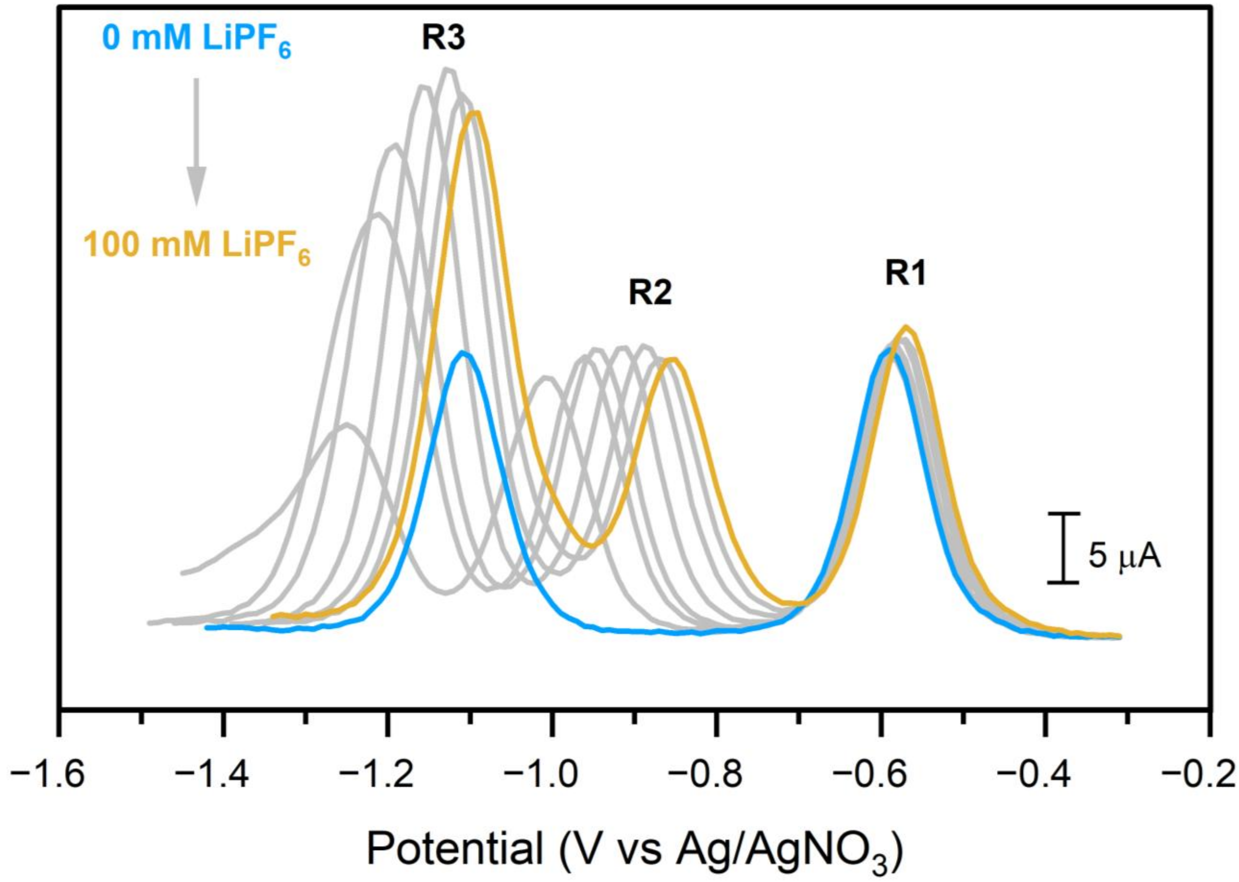

Figure S12. SWV of 1 mM of $(TBA)_3(PW_{12})$ in MeCN containing 100 mM of $TBAPF_6$ and variable amounts of $LiPF_6$ (0–100 mM) supporting electrolytes collected at room temperature (~19–21 °C) using a pulse height of 25 mV, pulse width of 100 ms, and step height of 10 mV.

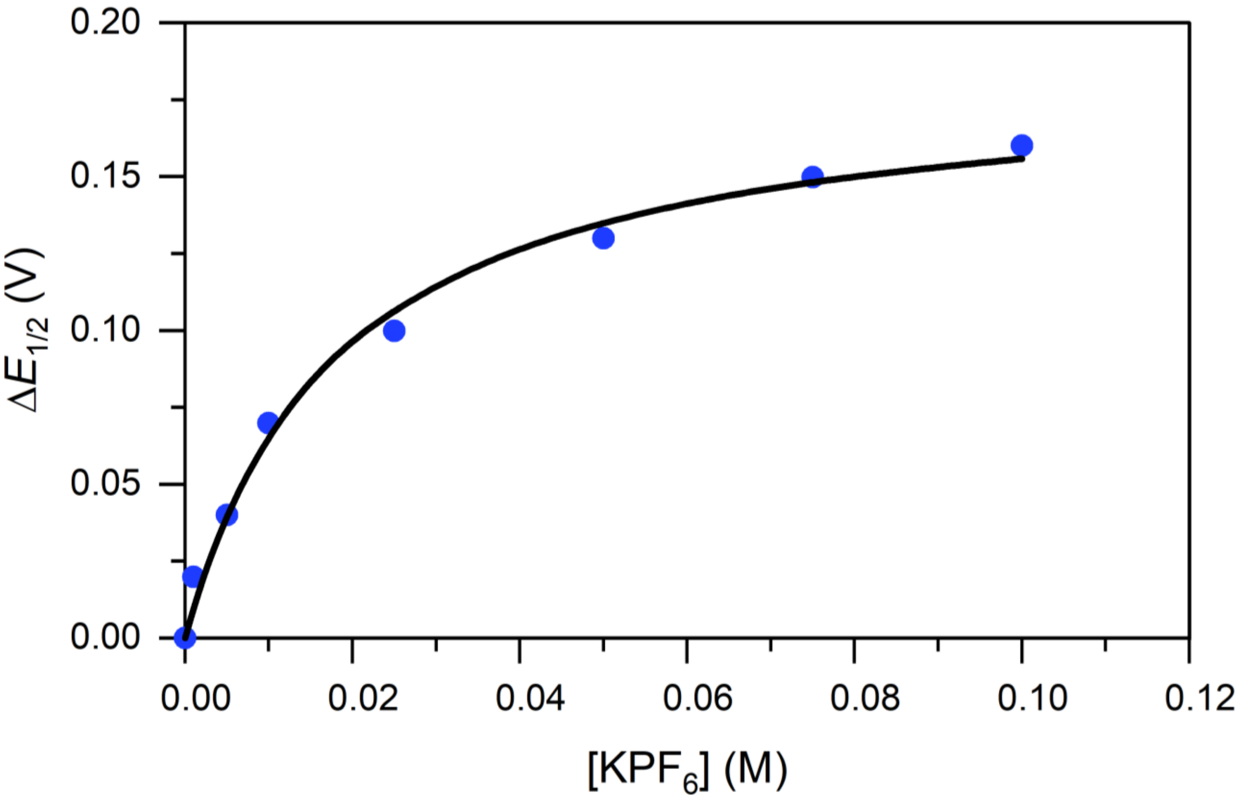

Figure S13. Plot of the difference in $E_{1/2}$ for the R2 couple of $(TBA)_3(PW_{12})$ in the presence and absence of variable amounts of KPF_6 in MeCN extracted from the SWV titration data shown in Figure S11. Blue circles denote experimental data, and the black line corresponds to a Langmuir fit to the data (eq 1). Using eq 1, an average value of K_a = 54(1) M^{-1} with $R^2 > 0.99$ was obtained. The error in the average value of K_a corresponds to the standard deviation of two independent measurements.

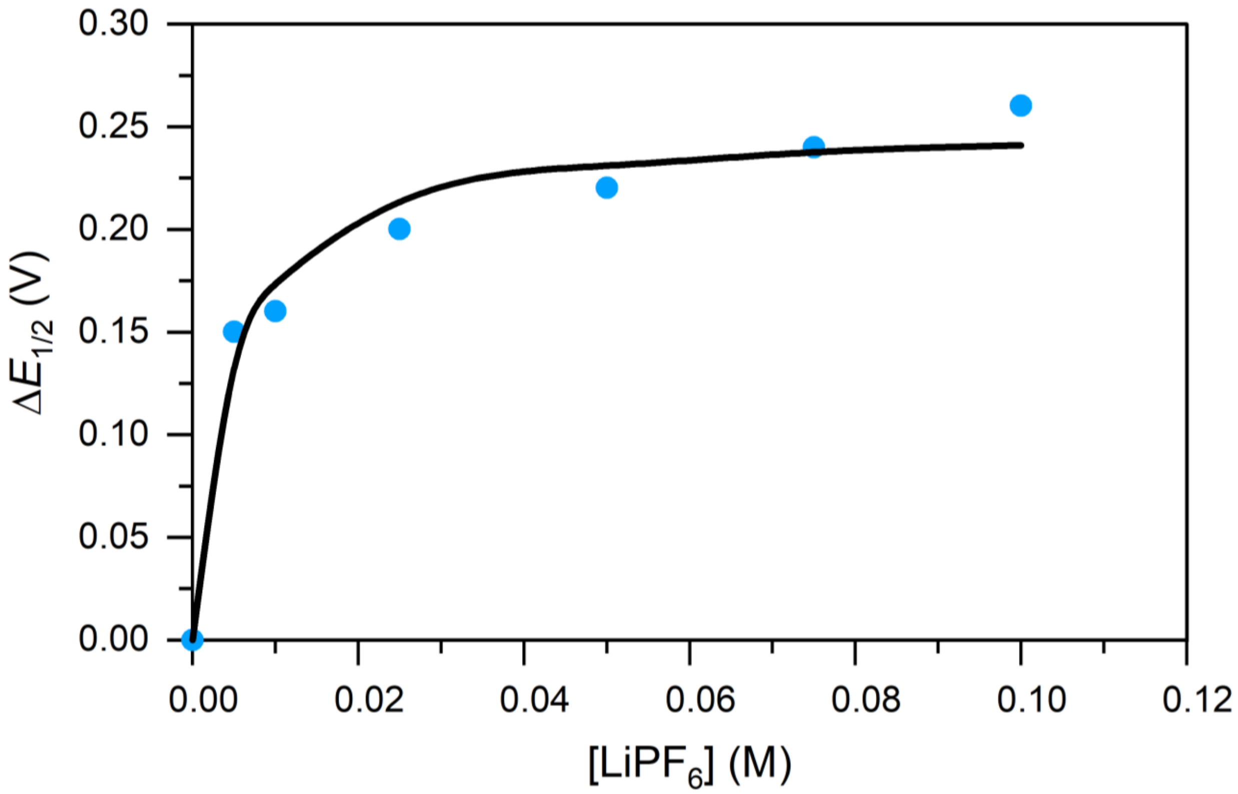

Figure S14. Plot of the difference in $E_{1/2}$ for the R2 couple of $(TBA)_3(PW_{12})$ in the presence and absence of variable amounts of $LiPF_6$ in MeCN extracted from the SWV titration data shown in Figure S12. Blue circles denote experimental data, and the black line corresponds to a Langmuir fit to the data (eq 1). Using eq 1, an average value of K_a = 212(12) M^{-1} with R^2 >0.97 was obtained. The error in the average value of K_a corresponds to the standard deviation of three independent measurements.

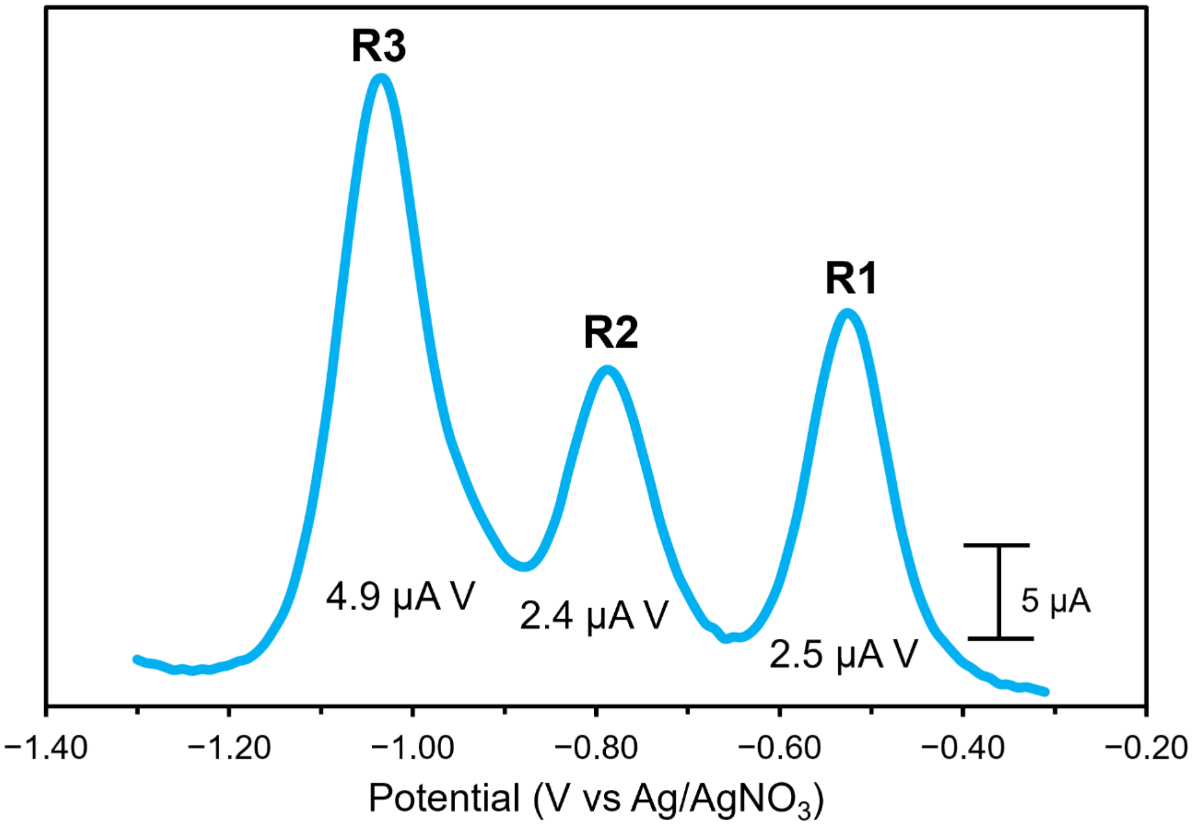

Figure S15. SWV of 1 mM of $(TBA)_3(PW_{12})$ in MeCN containing 100 mM of $LiPF_6$ supporting electrolyte collected at room temperature (~19–21 °C) using a pulse height of 25 mV, pulse width of 100 ms, and step height of 10 mV to probe the number of electrons involved in each reduction process. Values labelled on the graph correspond to integrations of the area under the curve, obtained using a designated integration tool in the EC-Lab software. The relative peak areas correspond to the associated number of electrons, i.e., one for the first two reductions and two for the third reduction.

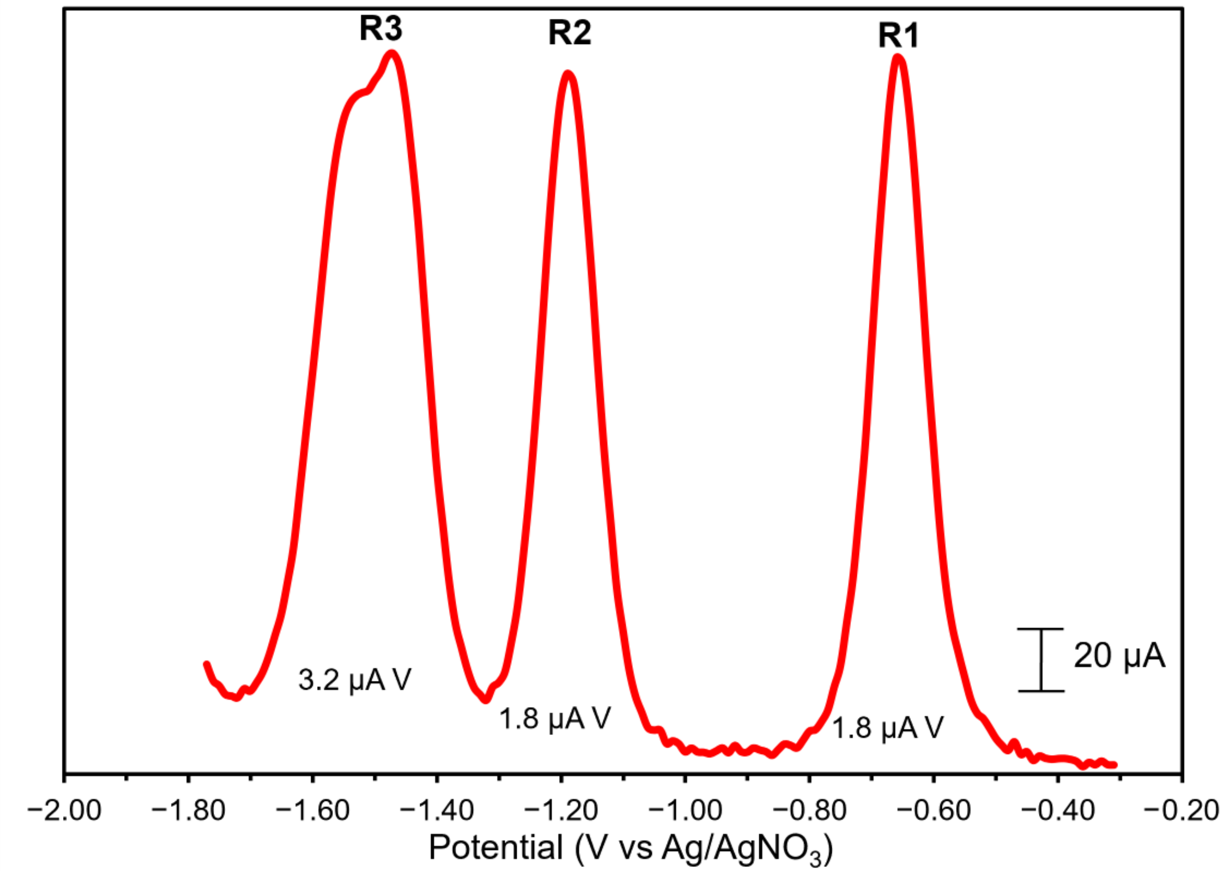

Figure S16. SWV of 1 mM of $(TBA)_3(PW_{12})$ in DMF containing 100 mM of $LiPF_6$ supporting electrolyte collected at room temperature (~19–21 °C) using a pulse height of 25 mV, pulse width of 100 ms, and step height of 10 mV to probe the number of electrons involved in each reduction process. Values labelled on the graph correspond to integrations of the area under the curve, obtained using a designated integration tool in the EC-Lab software. The relative peak areas correspond to the associated number of electrons, i.e., one for the first two reductions and two for the third reduction.

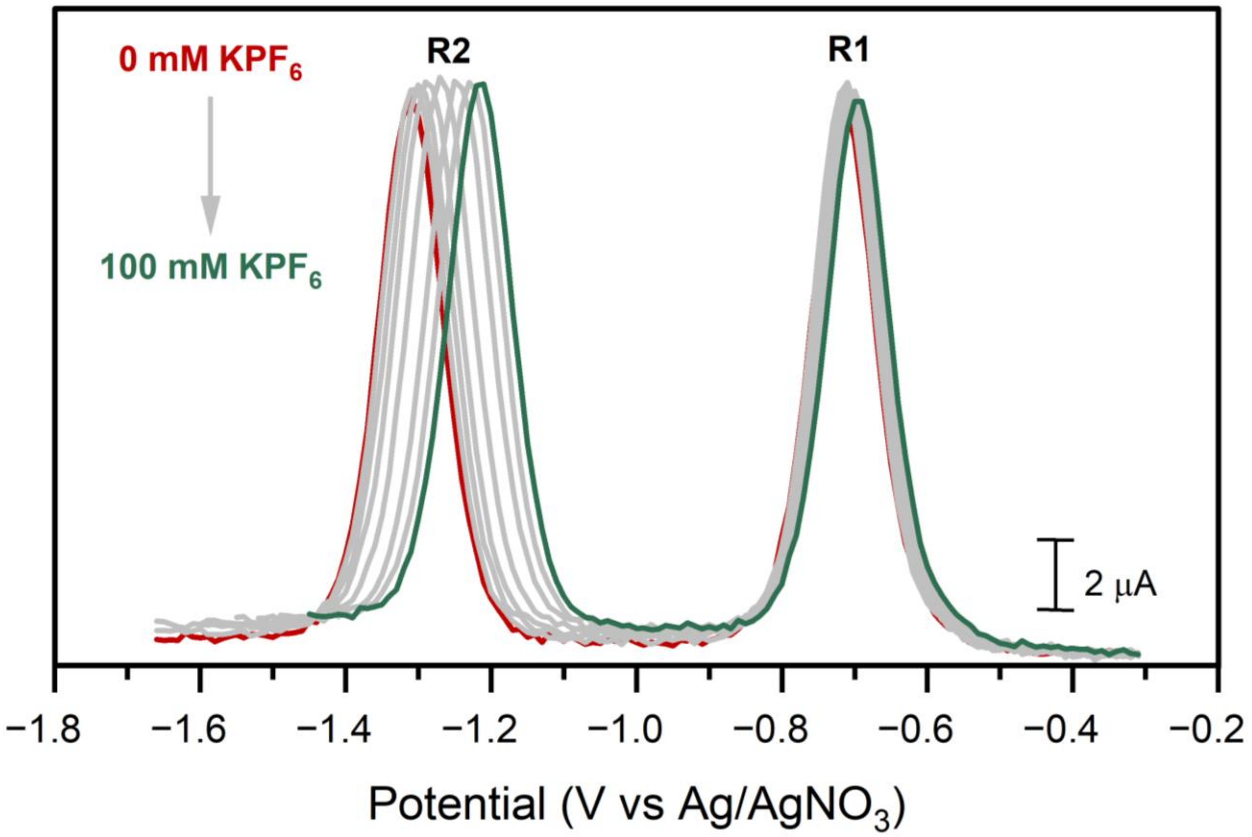

Figure S17. SWV of 1 mM of $(TBA)_3(PW_{12})$ in DMF containing 100 mM of $TBAPF_6$ and variable amounts of KPF_6 (0–100 mM) supporting electrolytes collected at room temperature (~19–21 °C) using a pulse height of 25 mV, pulse width of 100 ms, and step height of 10 mV.

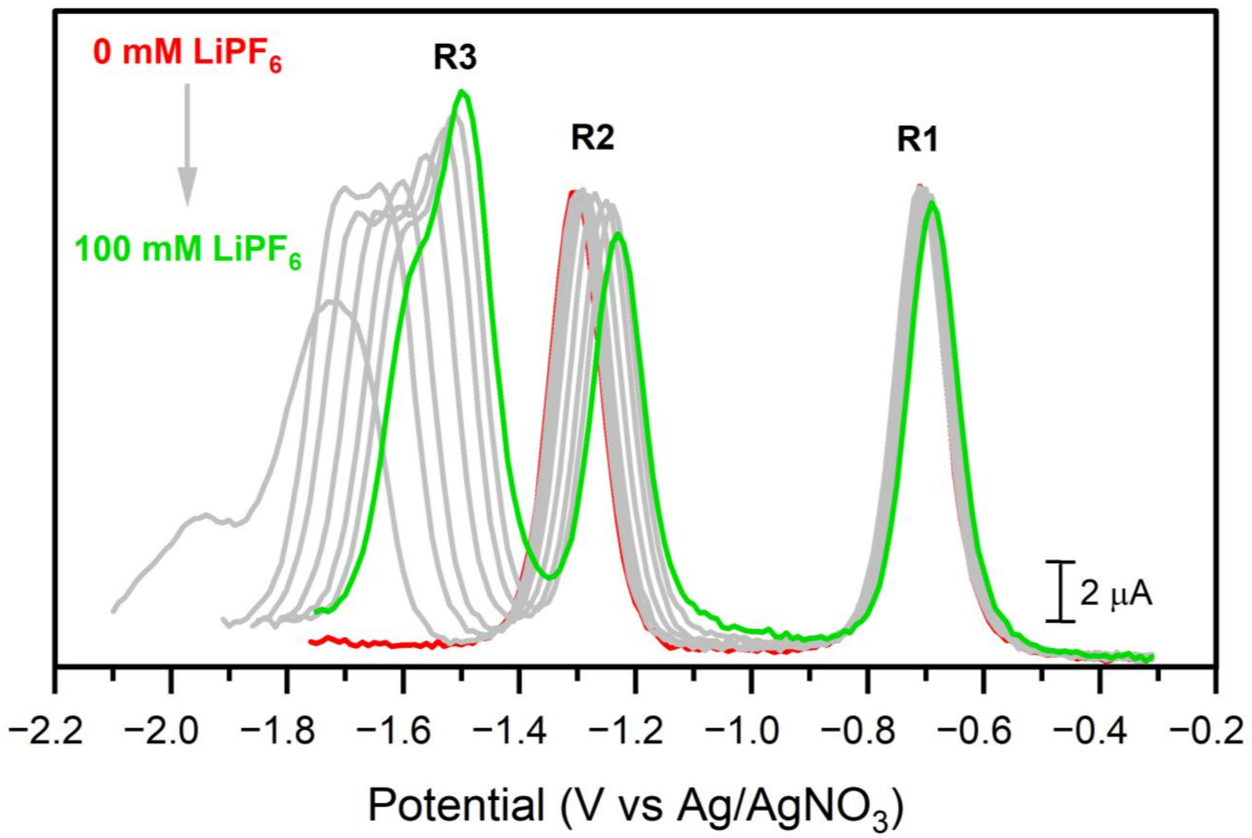

Figure S18. SWV of 1 mM of $(TBA)_3(PW_{12})$ in DMF containing 100 mM of $TBAPF_6$ and variable amounts of $LiPF_6$ (0–100 mM) supporting electrolytes collected at room temperature (~19–21 °C) using a pulse height of 25 mV, pulse width of 100 ms, and step height of 10 mV.

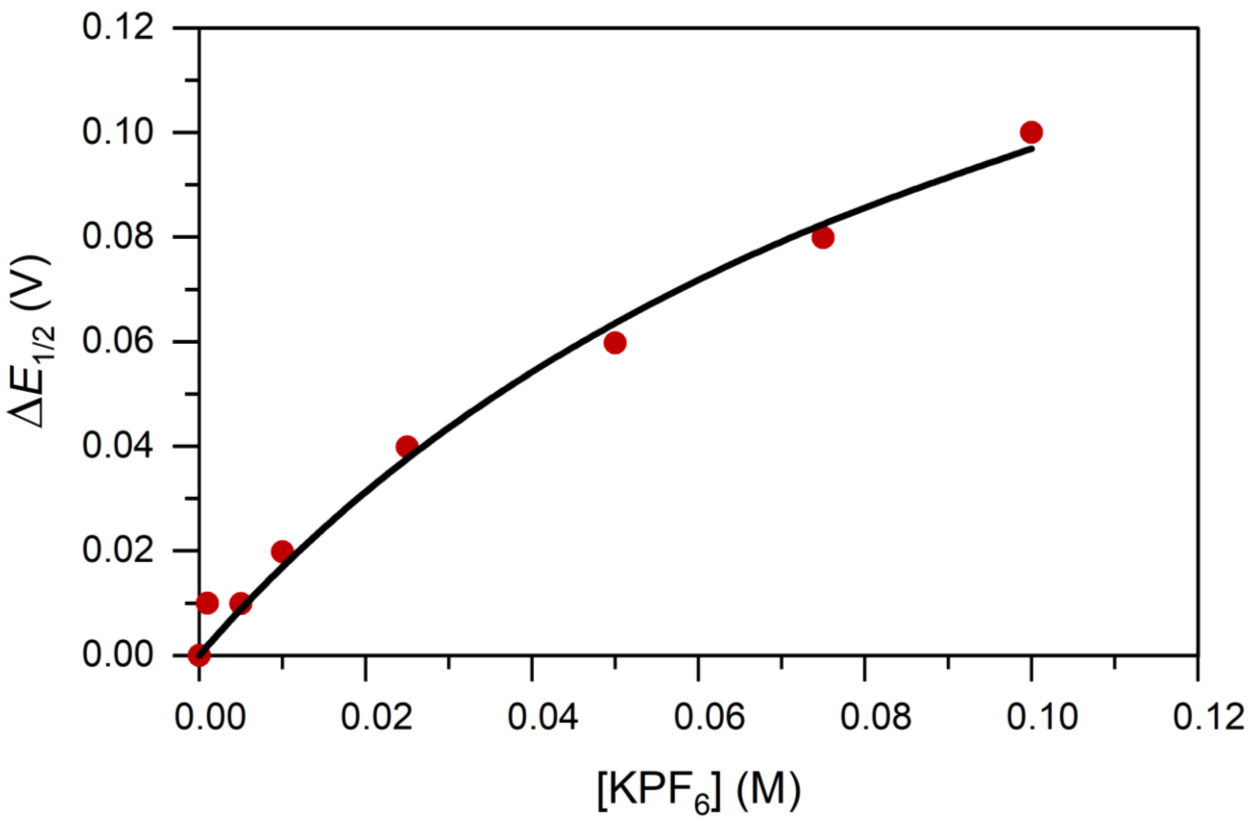

Figure S19. Plot of the difference in $E_{1/2}$ for the R2 couple of $(TBA)_3(PW_{12})$ in the presence and absence of variable amounts of KPF_6 in DMF extracted from the SWV titration data shown in Figure S17. Red circles denote experimental data, and the black line corresponds to a Langmuir fit to the data (eq 1). Using eq 1, an average value of K_a = 11(2) M^{-1} with $R^2 > 0.99$ was obtained. The error in the average value of K_a corresponds to the standard deviation of two independent measurements.

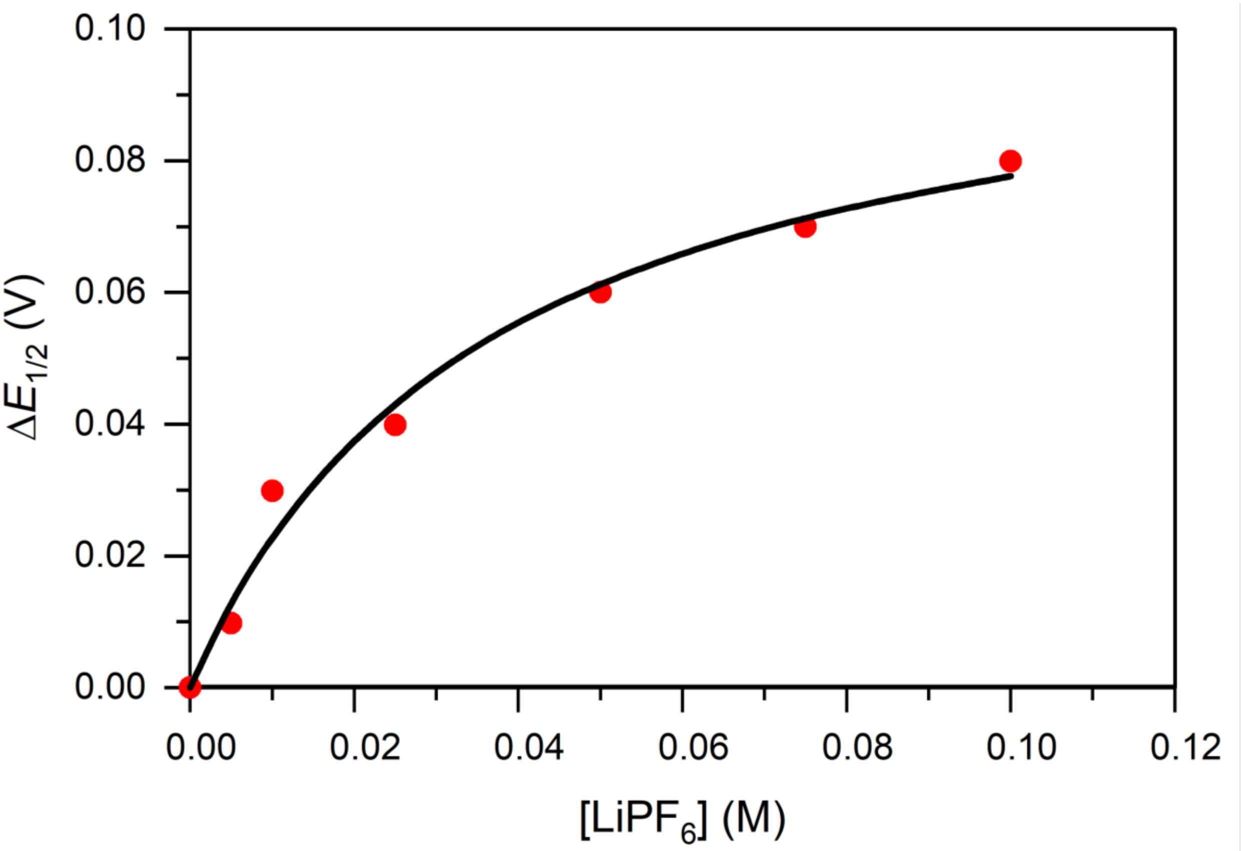

Figure S20. Plot of the difference in $E_{1/2}$ for the R2 couple of $(TBA)_3(PW_{12})$ in the presence and absence of variable amounts of $LiPF_6$ in DMF extracted from the SWV titration data shown in Figure S18. Red circles denote experimental data, and the black line corresponds to a Langmuir fit to the data (eq 1). Using eq 1, an average value of K_a = 20(12) M^{-1} with R^2 > 0.99 was obtained. The error in the average value of K_a corresponds to the standard deviation of two independent measurements.

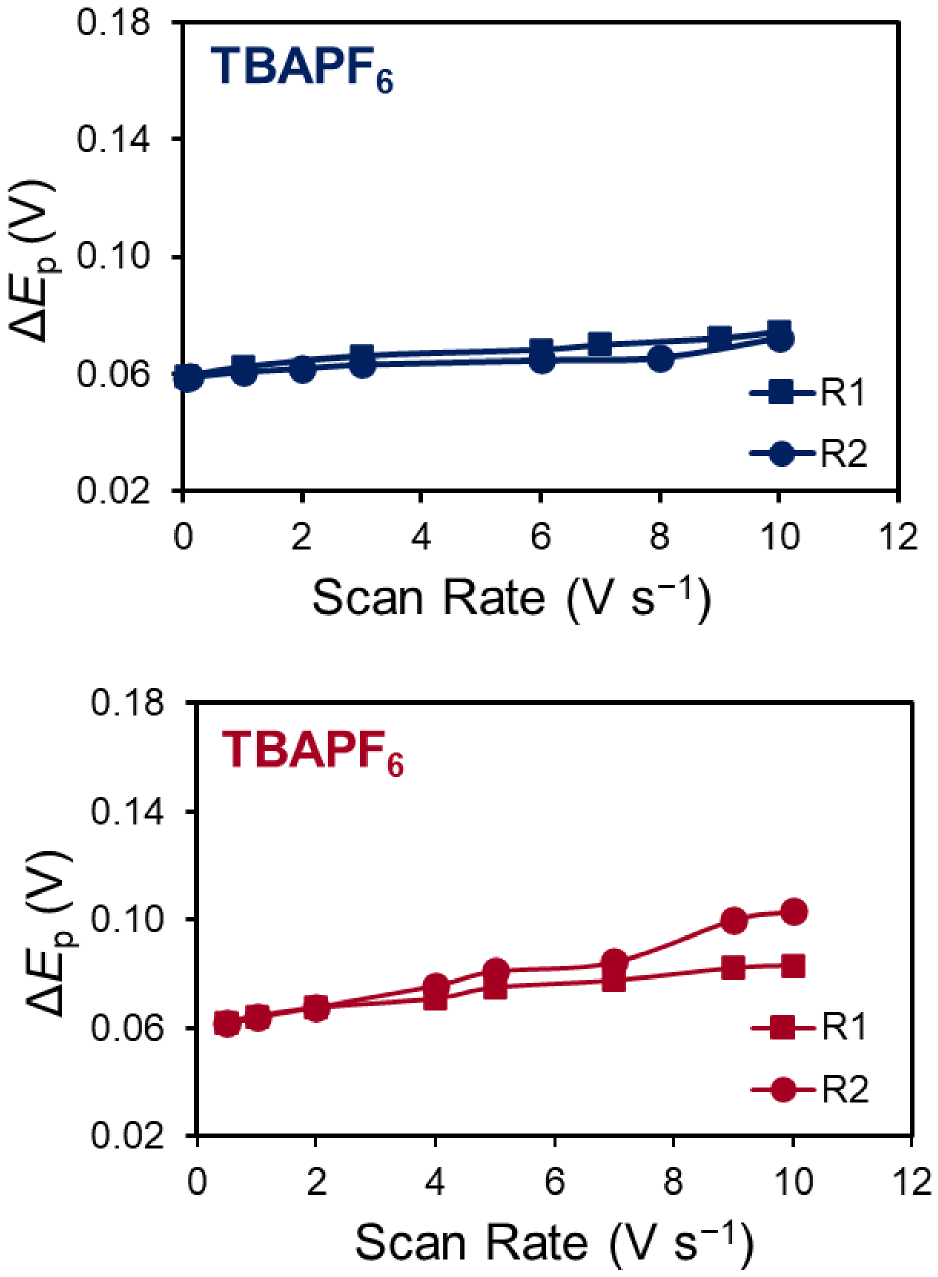

Figure S21. Plots of cathodic and anodic peak-to-peak separation (ΔE_p) as a function of scan rate for 1 mM of $(TBA)_3(PW_{12})$ in MeCN (top) and DMF (bottom) containing 100 mM of $TBAPF_6$ supporting electrolyte, obtained from variable-scan-rate measurements (Figures S24, S25, S38, S39) at room temperature (~19–21 °C). Lines between data points are a guide to the eye.

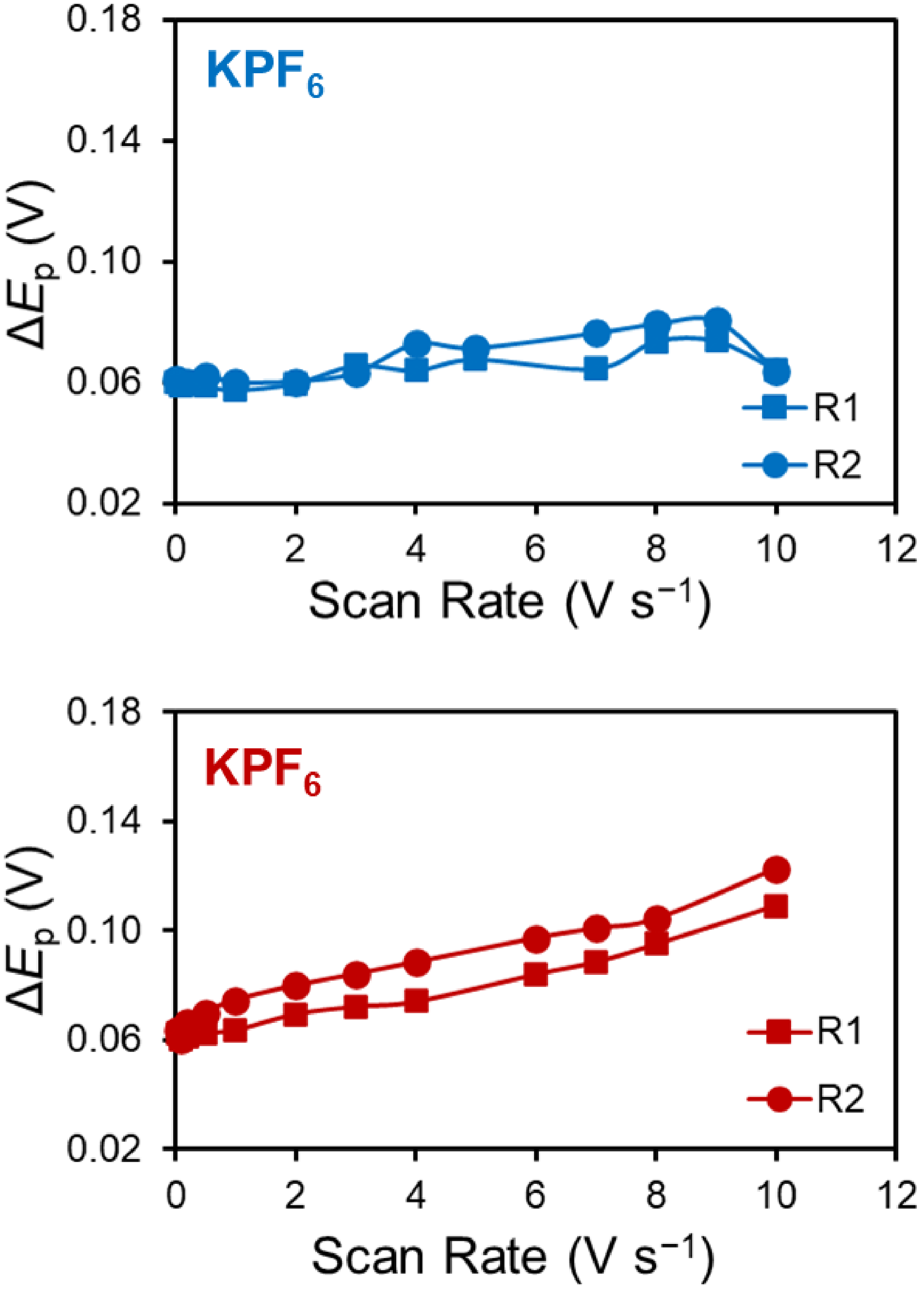

Figure S22. Plots of cathodic and anodic peak-to-peak separation (ΔE_p) as a function of scan rate for 1 mM of $(TBA)_3(PW_{12})$ in MeCN (top) and DMF (bottom) containing 100 mM of KPF_6 supporting electrolyte, obtained from variable-scan-rate measurements (Figures S28, S29, S42, S43) at room temperature (~19–21 °C). Lines between data points are a guide to the eye.

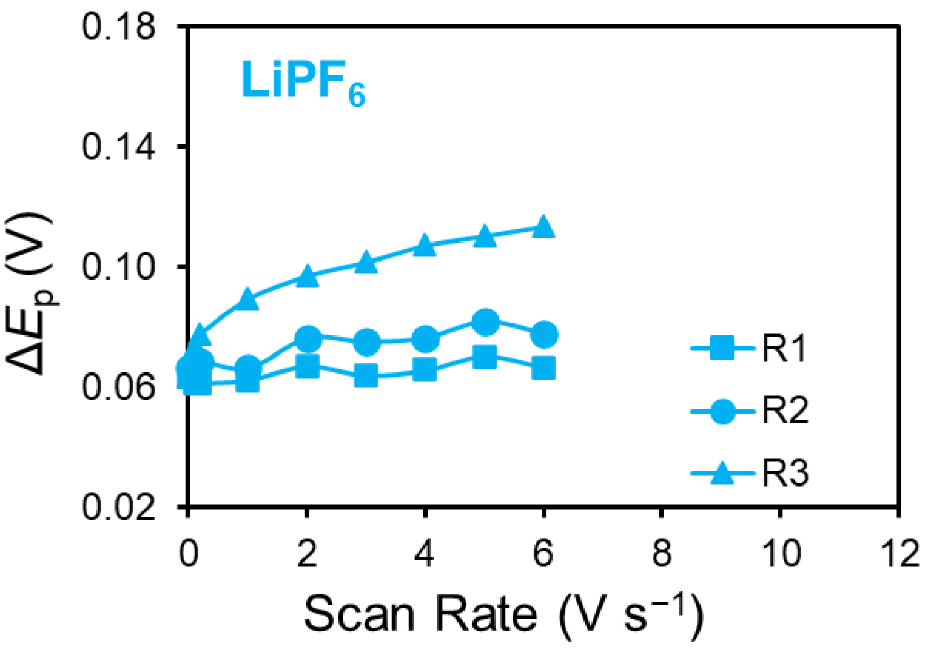

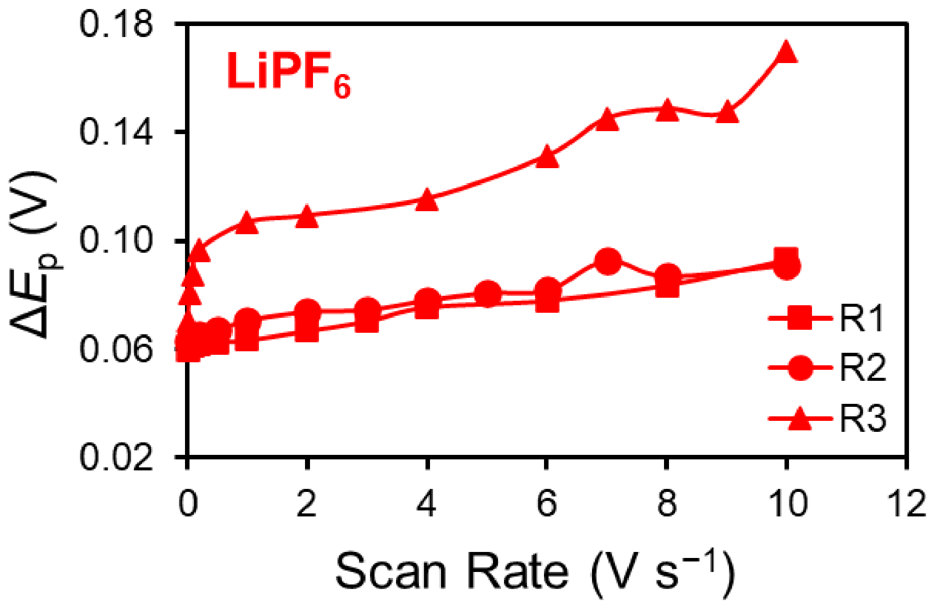

Figure S23. Plots of cathodic and anodic peak-to-peak separation (ΔE_p) as a function of scan rate for 1 mM of $(TBA)_3(PW_{12})$ in MeCN (top) and DMF (bottom) containing 100 mM of $LiPF_6$ supporting electrolyte, obtained from variable-scan-rate measurements (Figures S32–S34, S46–S48) at room temperature (~19–21 °C). Lines between data points are a guide to the eye.

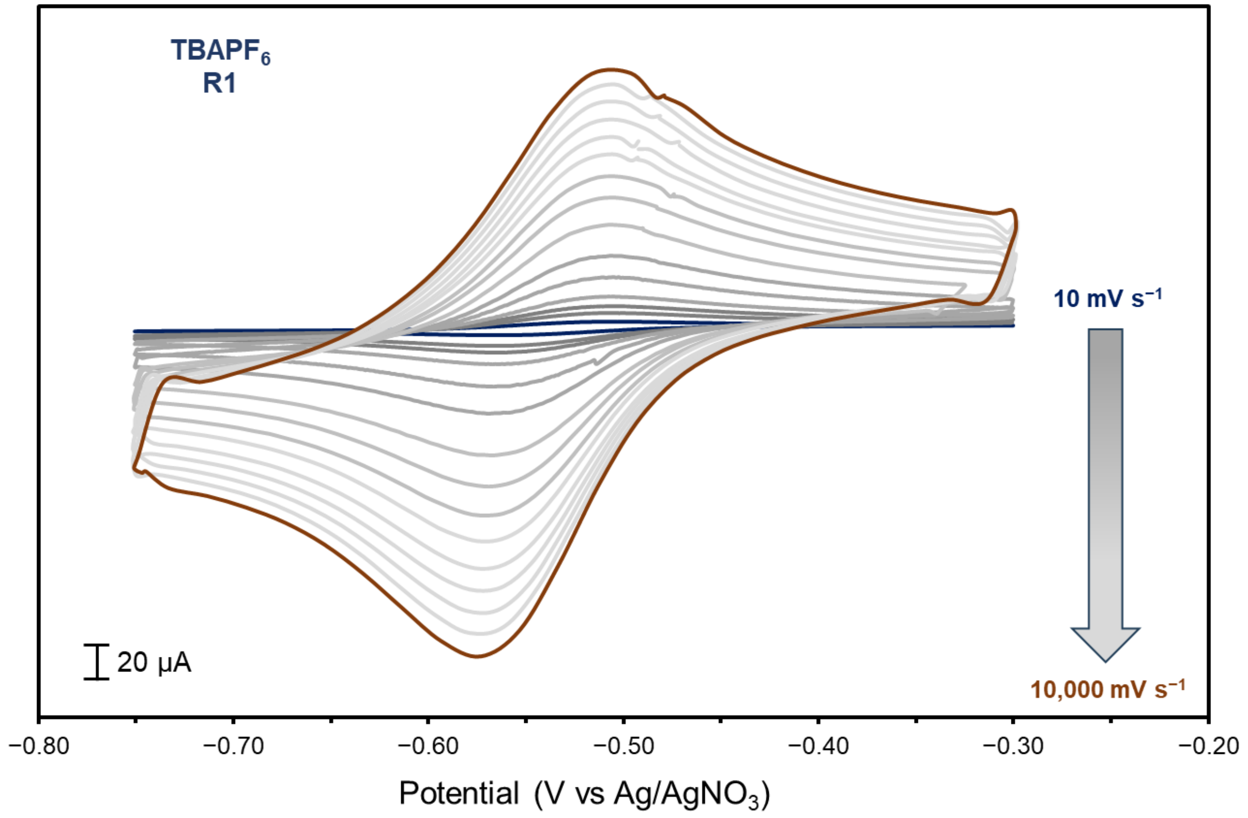

Figure S24. CVs of 1 mM of $(TBA)_3(PW_{12})$ in MeCN containing 100 mM of $TBAPF_6$ supporting electrolyte collected at room temperature (~19–21 °C) at variable scan rates (10–10,000 mV s^{-1}). The R1 redox couple is displayed. Observed spikes in the data are the results of instrumental artifacts at fast scan rates.

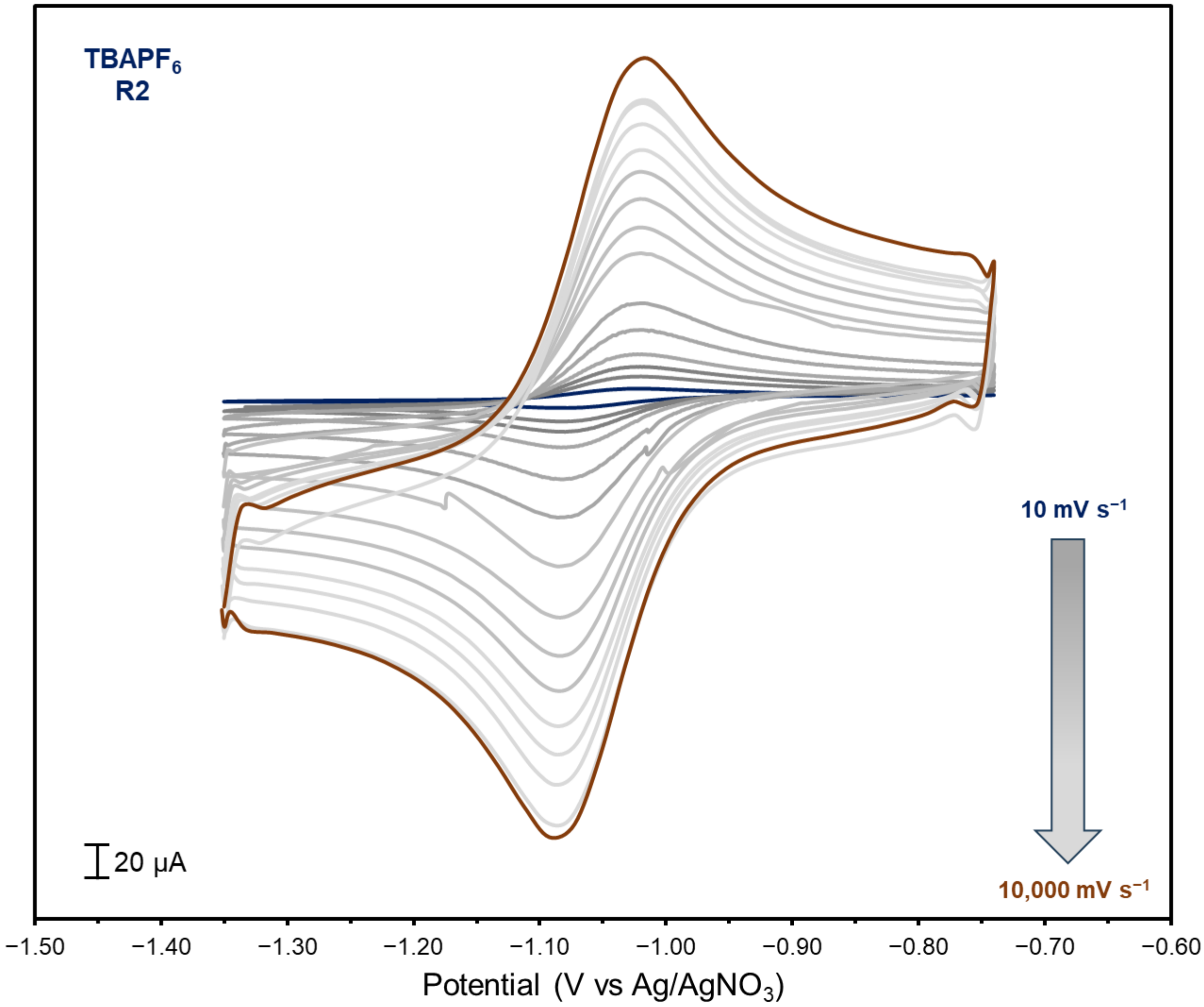

Figure S25. CVs of 1 mM of $(TBA)_3(PW_{12})$ in MeCN containing 100 mM of $TBAPF_6$ supporting electrolyte collected at room temperature (~19–21 °C) at variable scan rates (10–10,000 mV s^{-1}). The R2 redox couple is displayed. Observed spikes in the data are the results of instrumental artifacts at fast scan rates.

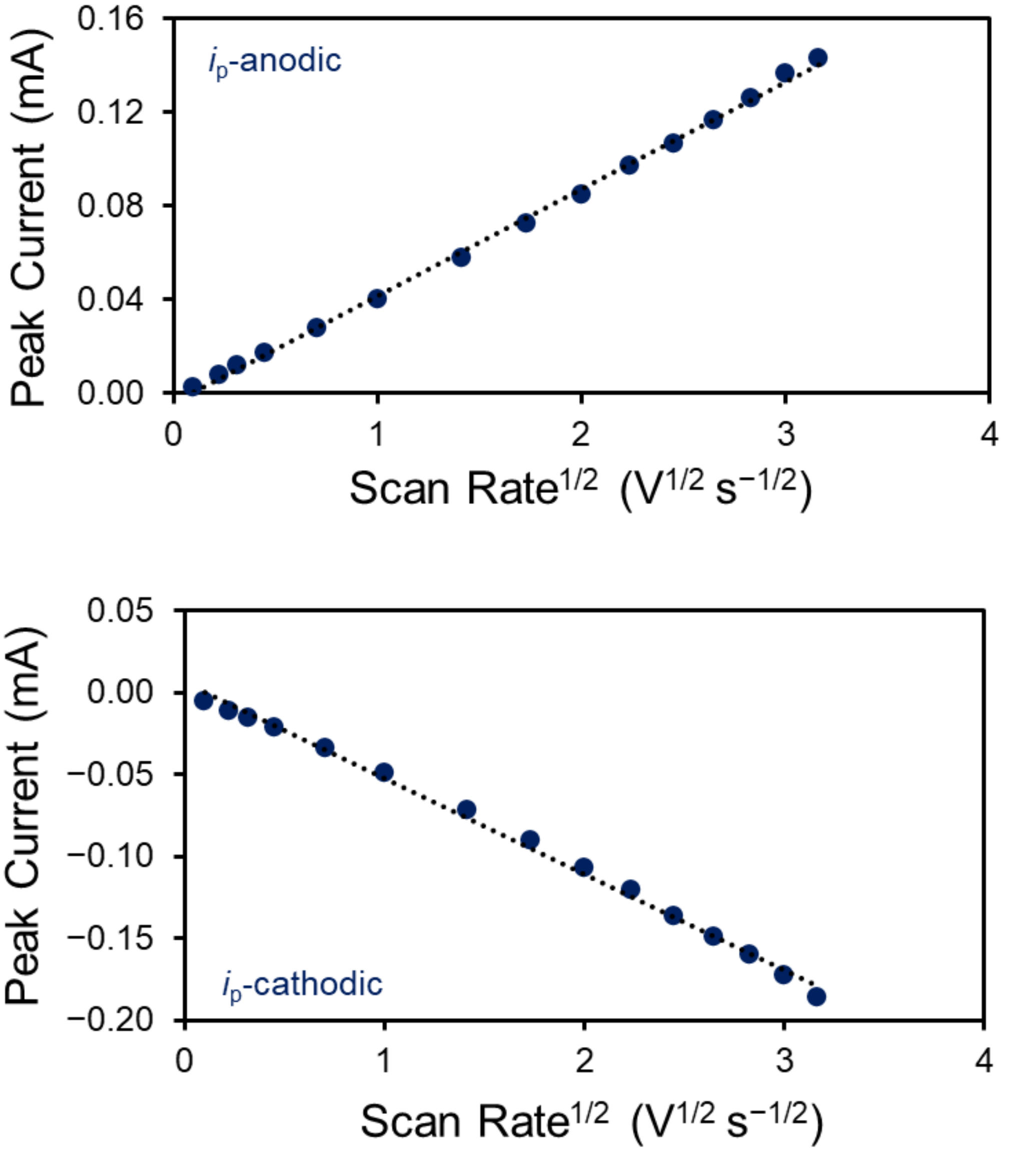

Figure S26. Randles–Ševčík analysis of the R1 redox couple of $(TBA)_3(PW_{12})$ in MeCN containing 100 mM of $TBAPF_6$ supporting electrolyte at room temperature (~19–21 °C), obtained from variable-scan-rate CV data (Figure S24). i_p-anodic and i_p-cathodic denote the anodic and cathodic peak currents, respectively. Blue circles denote experimental data, and the black dotted lines represent linear fits to the data. A temperature of 20 °C was used for analysis.

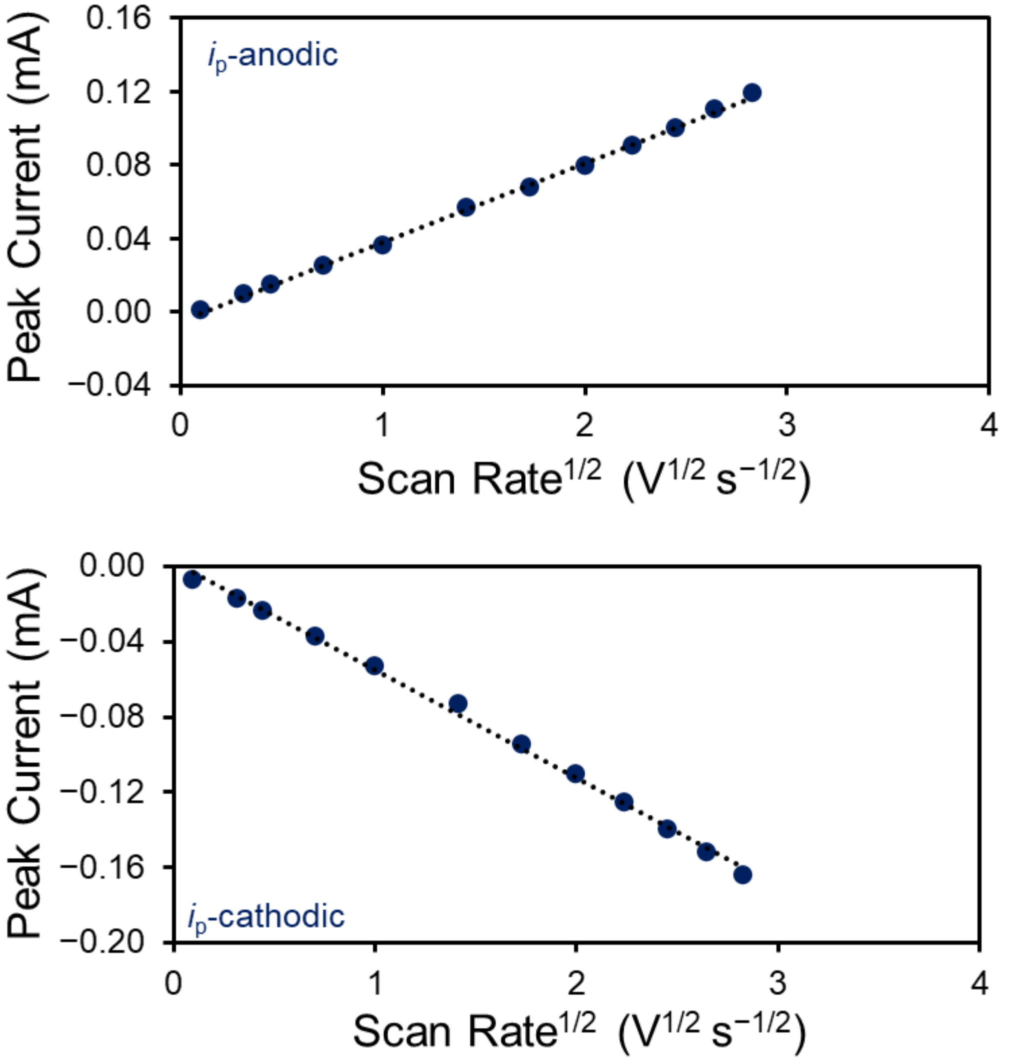

Figure S27. Randles–Ševčík analysis of the R2 redox couple of $(TBA)_3(PW_{12})$ in MeCN containing 100 mM of $TBAPF_6$ supporting electrolyte at room temperature (~19–21 °C), obtained from variable-scan-rate CV data (Figure S25). i_p-anodic and i_p-cathodic denote the anodic and cathodic peak currents, respectively. Blue circles denote experimental data, and the black dotted lines represent linear fits to the data. A temperature of 20 °C was used for analysis.

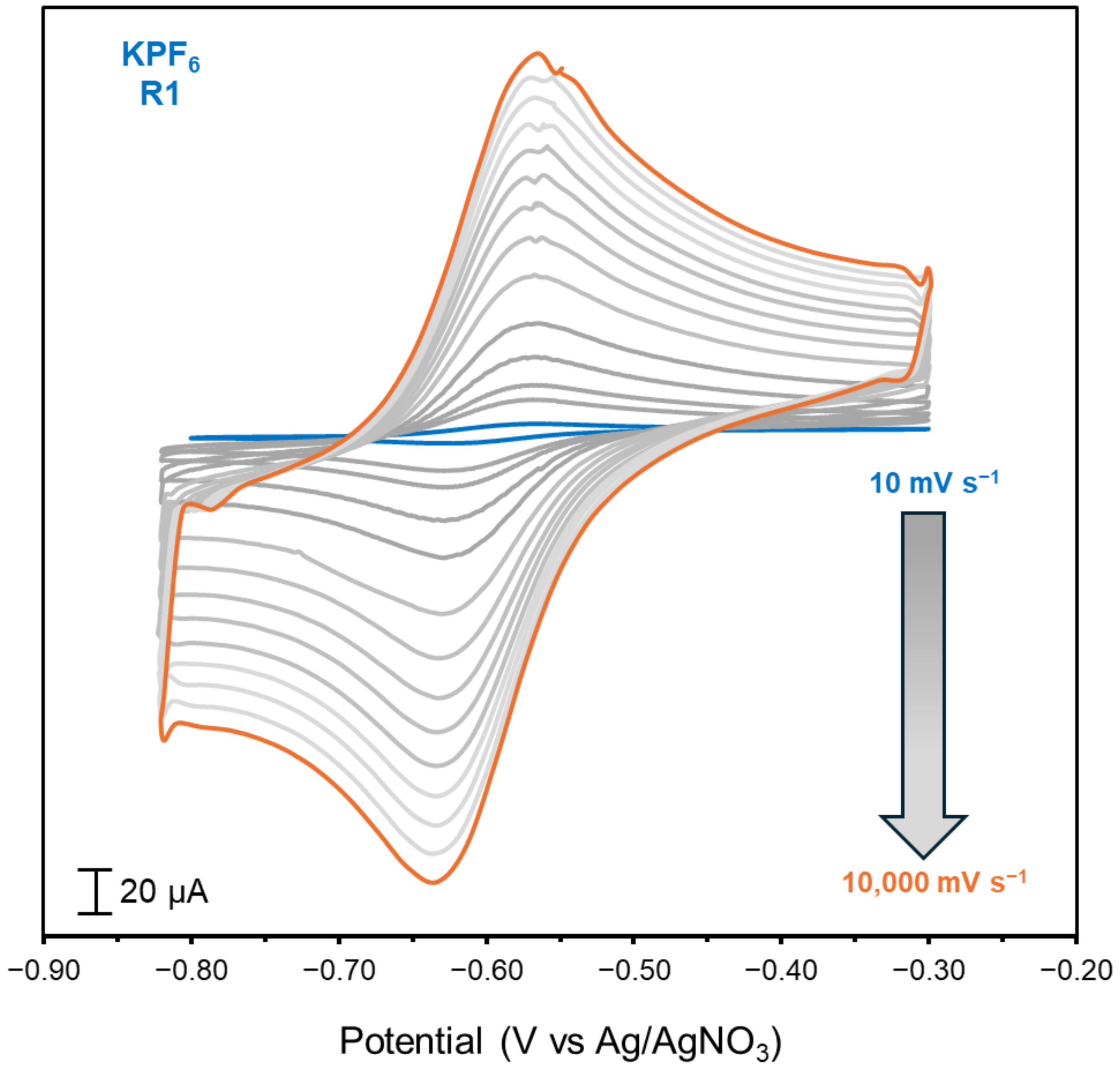

Figure S28. CVs of 1 mM of $(TBA)_3(PW_{12})$ in MeCN containing 100 mM of KPF_6 supporting electrolyte collected at room temperature (~19–21 °C) at variable scan rates (10–10,000 mV s^{-1}). The R1 redox couple is displayed. Observed spikes in the data are the results of instrumental artifacts at fast scan rates.

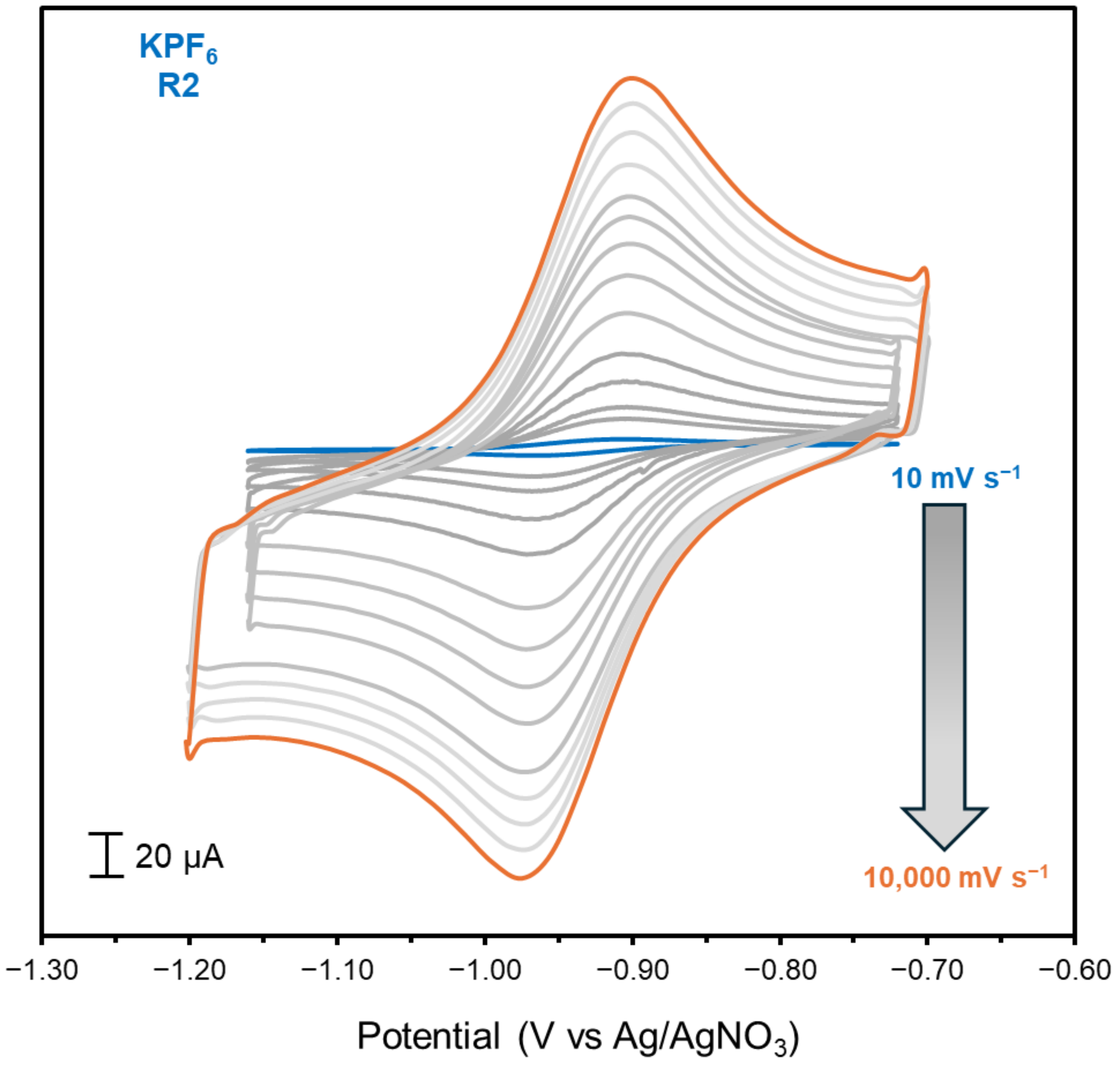

Figure S29. CVs of 1 mM of $(TBA)_3(PW_{12})$ in MeCN containing 100 mM of KPF_6 supporting electrolyte collected at room temperature (~19–21 °C) at variable scan rates (10–10,000 mV s^{-1}). The R2 redox couple is displayed. Observed spikes in the data are the results of instrumental artifacts at fast scan rates.

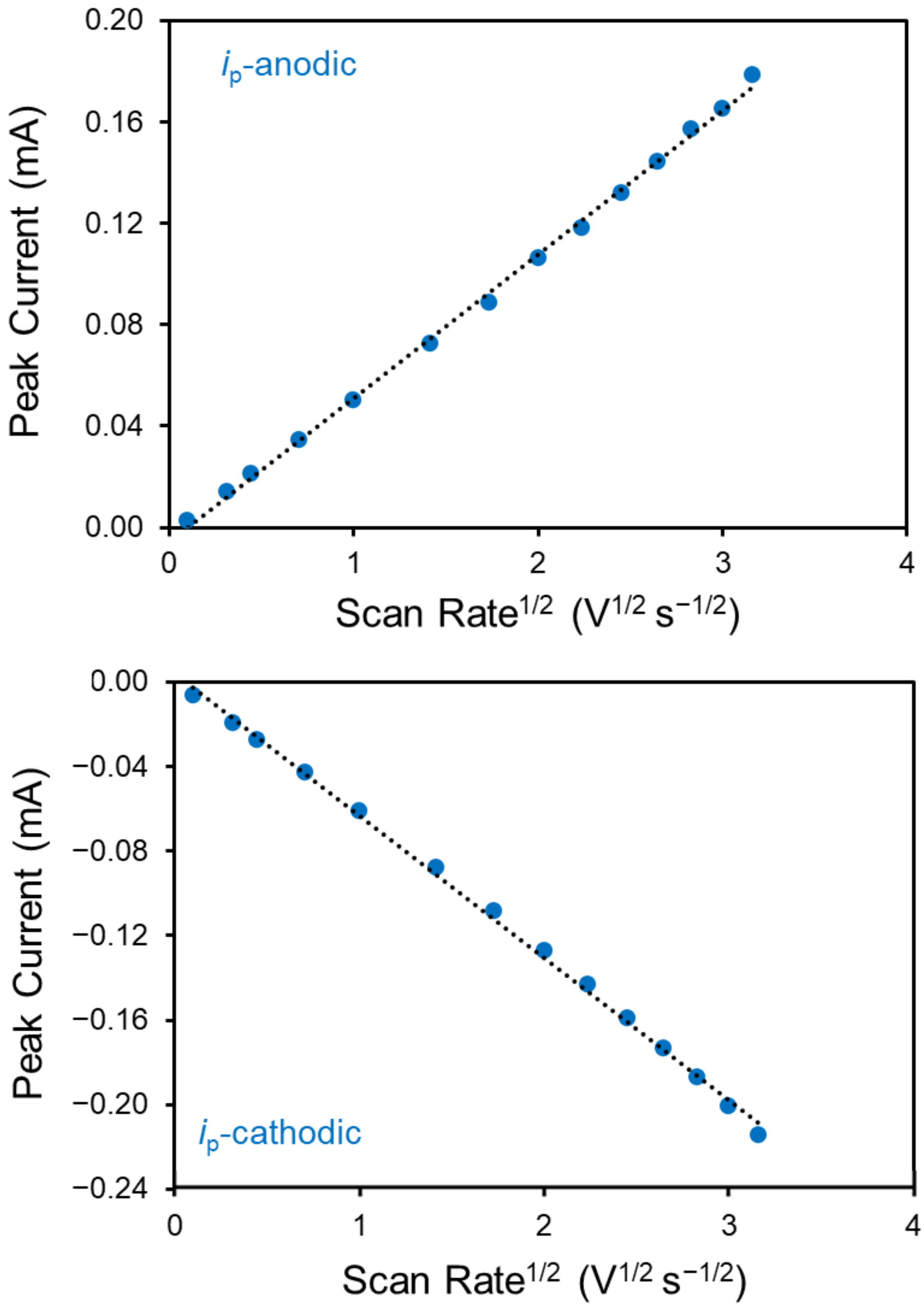

Figure S30. Randles–Ševčík analysis of the R1 redox couple of $(TBA)_3(PW_{12})$ in MeCN containing 100 mM of KPF_6 supporting electrolyte at room temperature (~19–21 °C), obtained from variable-scan-rate CV data (Figure S28). i_p-anodic and i_p-cathodic denote the anodic and cathodic peak currents, respectively. Blue circles denote experimental data, and the black dotted lines represent linear fits to the data. A temperature of 20 °C was used for analysis.

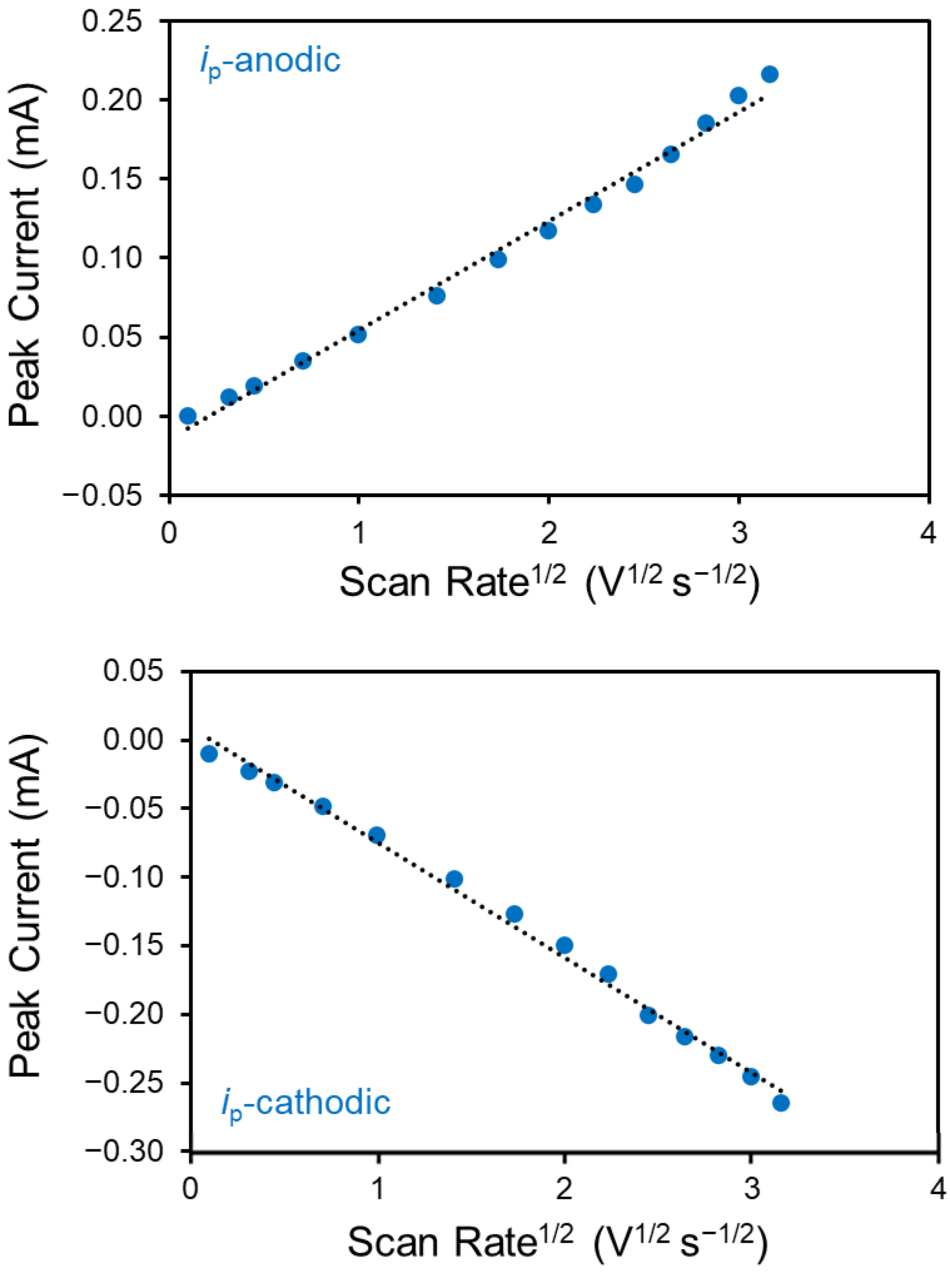

Figure S31. Randles–Ševčík analysis of the R2 redox couple of $(TBA)_3(PW_{12})$ in MeCN containing 100 mM of KPF_6 supporting electrolyte at room temperature (~19–21 °C), obtained from variable-scan-rate CV data (Figure S29). i_p-anodic and i_p-cathodic denote the anodic and cathodic peak currents, respectively. Blue circles denote experimental data, and the black dotted lines represent linear fits to the data. A temperature of 20 °C was used for analysis.

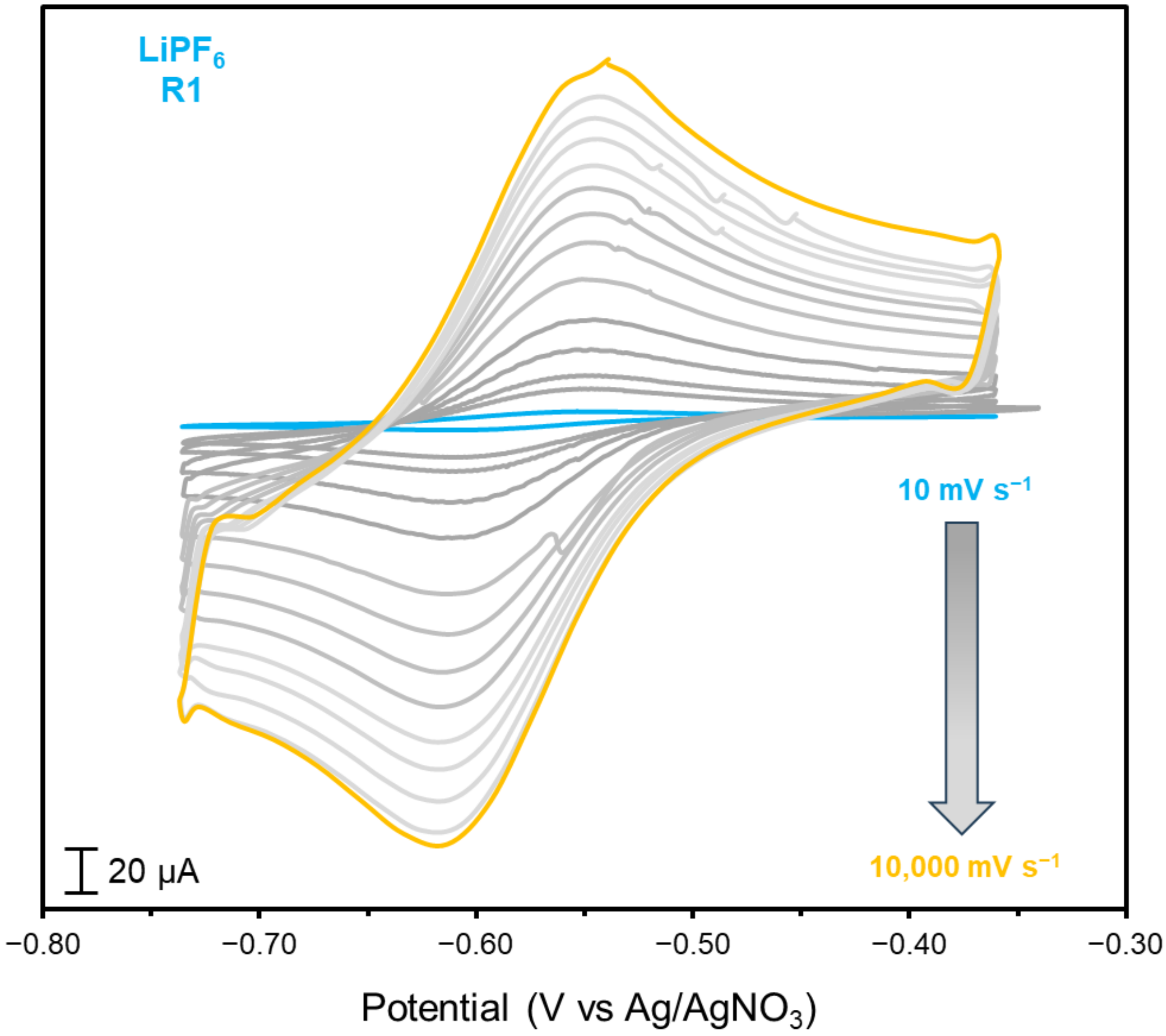

Figure S32. CVs of 1 mM of $(TBA)_3(PW_{12})$ in MeCN containing 100 mM of $LiPF_6$ supporting electrolyte collected at room temperature (~19–21 °C) at variable scan rates (10–10,000 mV s^{-1}). The R1 redox couple is displayed. Observed spikes in the data are the results of instrumental artifacts at fast scan rates.

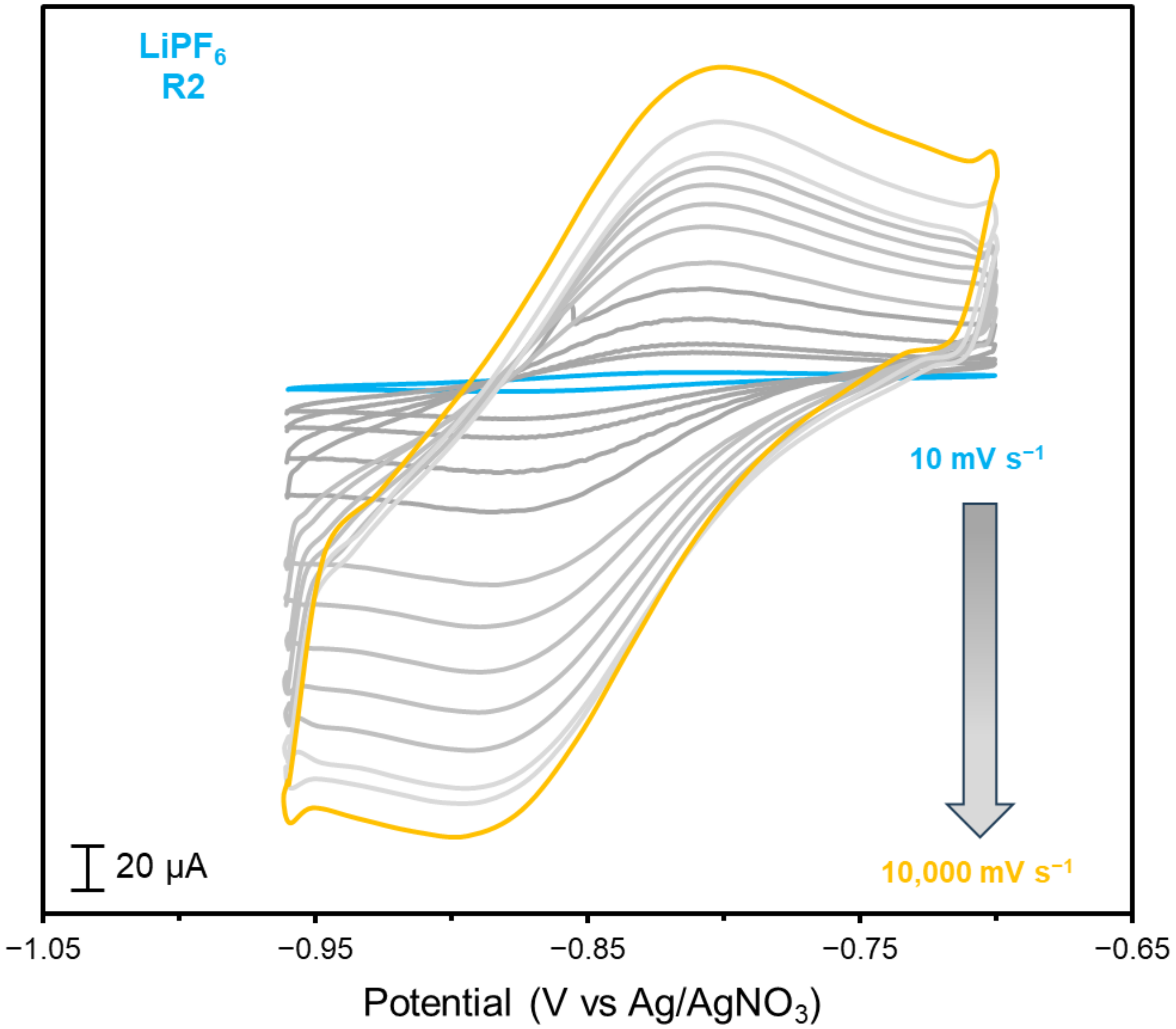

Figure S33. CVs of 1 mM of $(TBA)_3(PW_{12})$ in MeCN containing 100 mM of $LiPF_6$ supporting electrolyte collected at room temperature (~19–21 °C) at variable scan rates (10–10,000 mV s^{-1}). The R2 redox couple is displayed. Observed spikes in the data are the results of instrumental artifacts at fast scan rates.

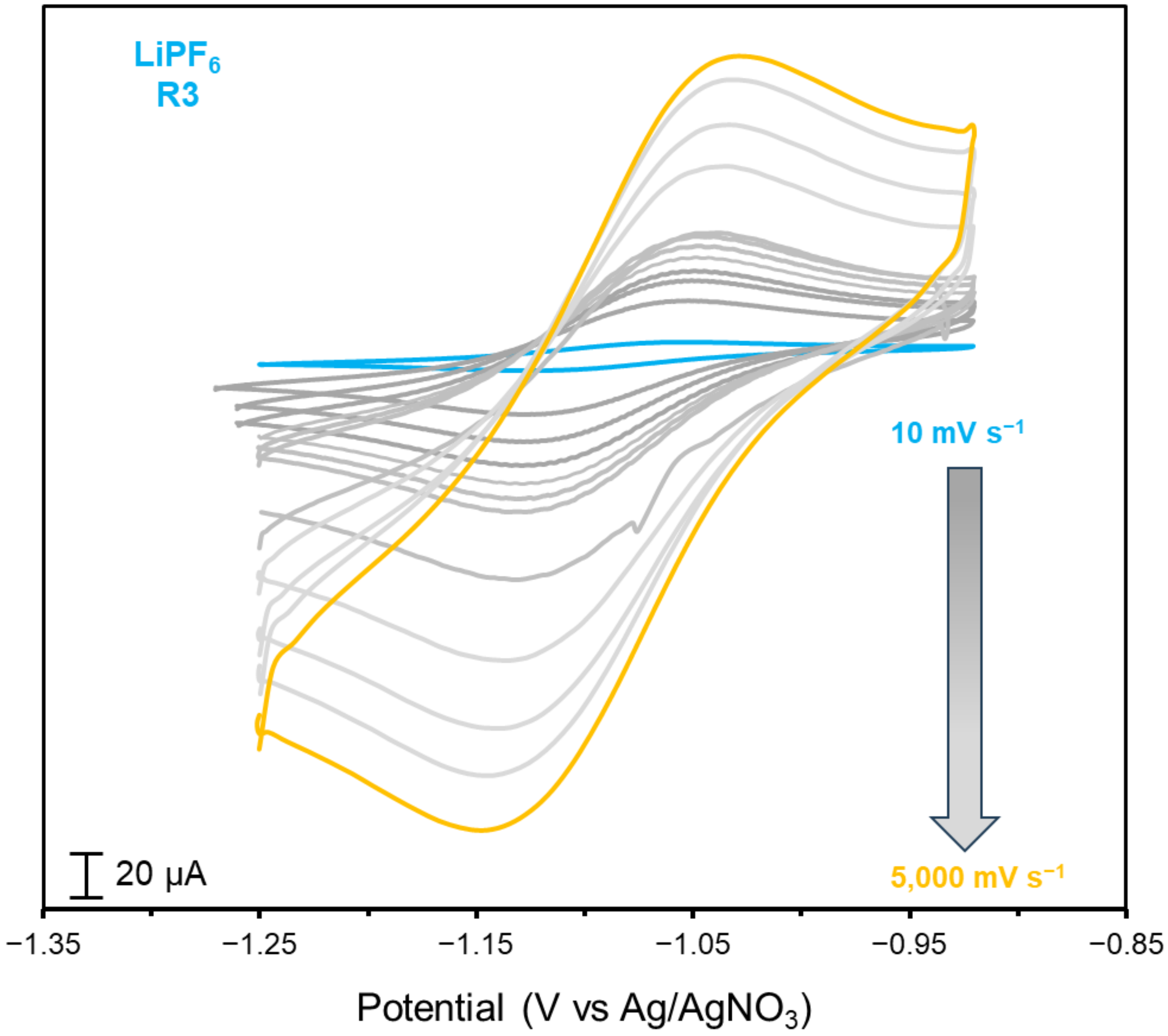

Figure S34. CVs of 1 mM of $(TBA)_3(PW_{12})$ in MeCN containing 100 mM of $LiPF_6$ supporting electrolyte collected at room temperature (~19–21 °C) at variable scan rates (10–5,000 mV s^{-1}). The R3 redox couple is displayed. Observed spikes in the data are the results of instrumental artifacts at fast scan rates.

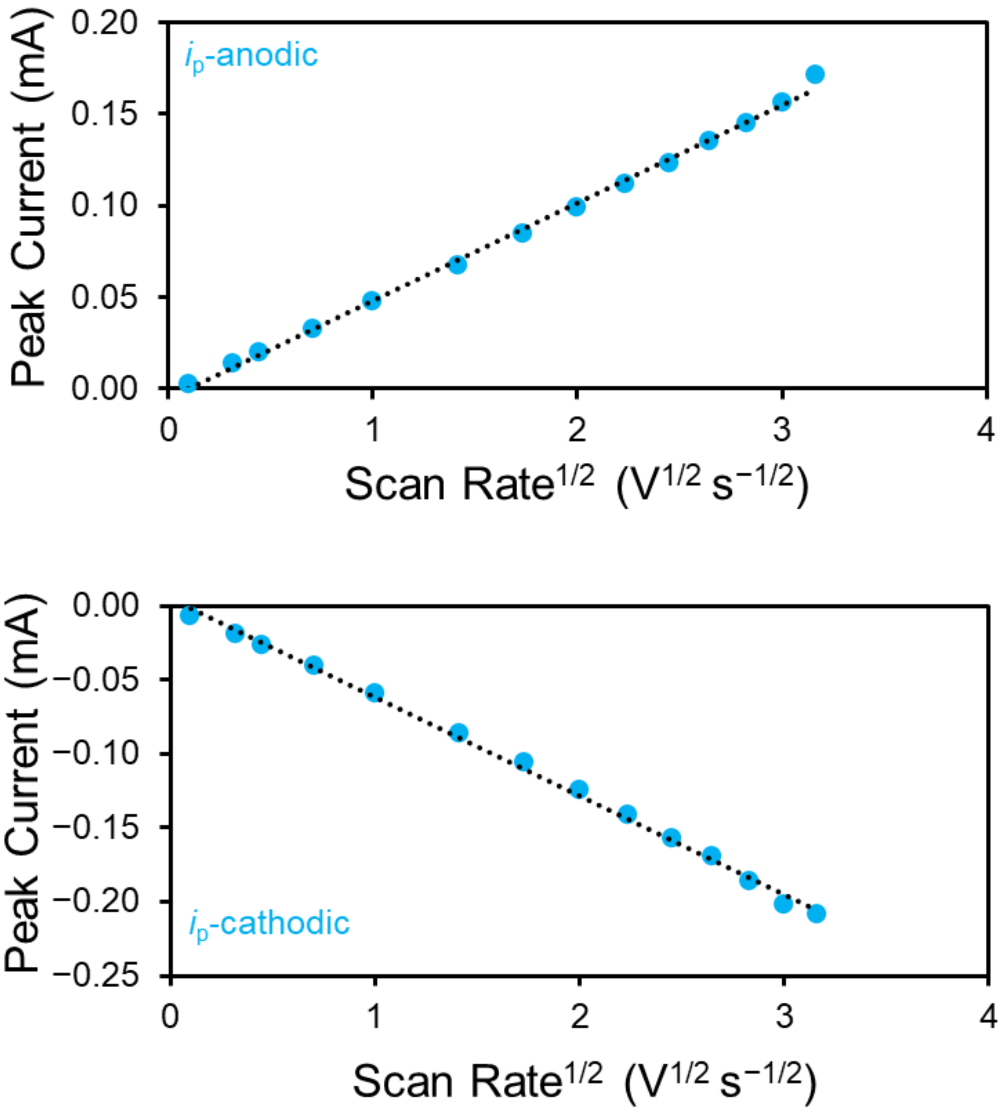

Figure S35. Randles–Ševčík analysis of the R1 redox couple of $(TBA)_3(PW_{12})$ in MeCN containing 100 mM of $LiPF_6$ supporting electrolyte at room temperature (~19–21 °C), obtained from variable-scan-rate CV data (Figure S32). i_p-anodic and i_p-cathodic denote the anodic and cathodic peak currents, respectively. Blue circles denote experimental data, and the black dotted lines represent linear fits to the data. A temperature of 20 °C was used for analysis.

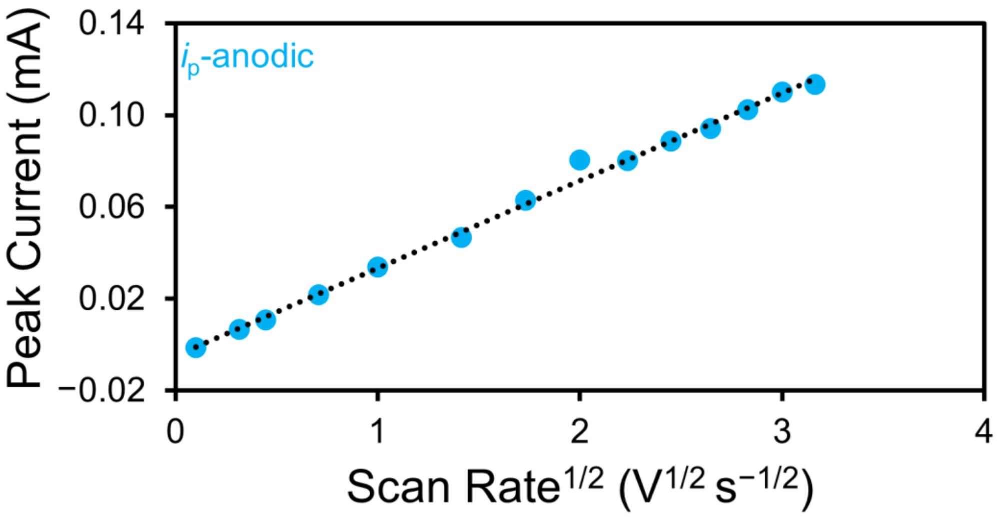

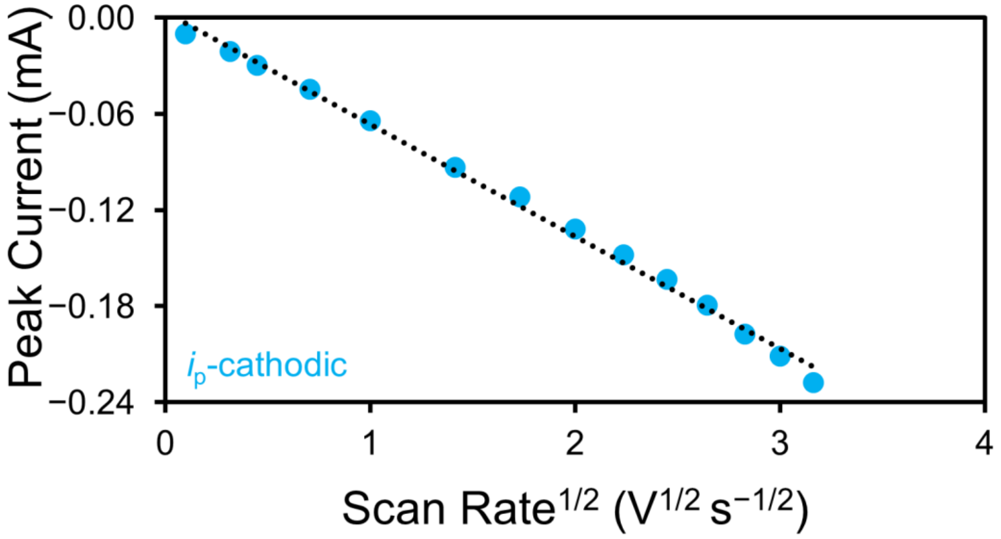

Figure S36. Randles–Ševčík analysis of the R2 redox couple of $(TBA)_3(PW_{12})$ in MeCN containing 100 mM of $LiPF_6$ supporting electrolyte at room temperature (~19–21 °C), obtained from variable-scan-rate CV data (Figure S33). i_p-anodic and i_p-cathodic denote the anodic and cathodic peak currents, respectively. Blue circles denote experimental data, and the black dotted lines represent linear fits to the data. A temperature of 20 °C was used for analysis.

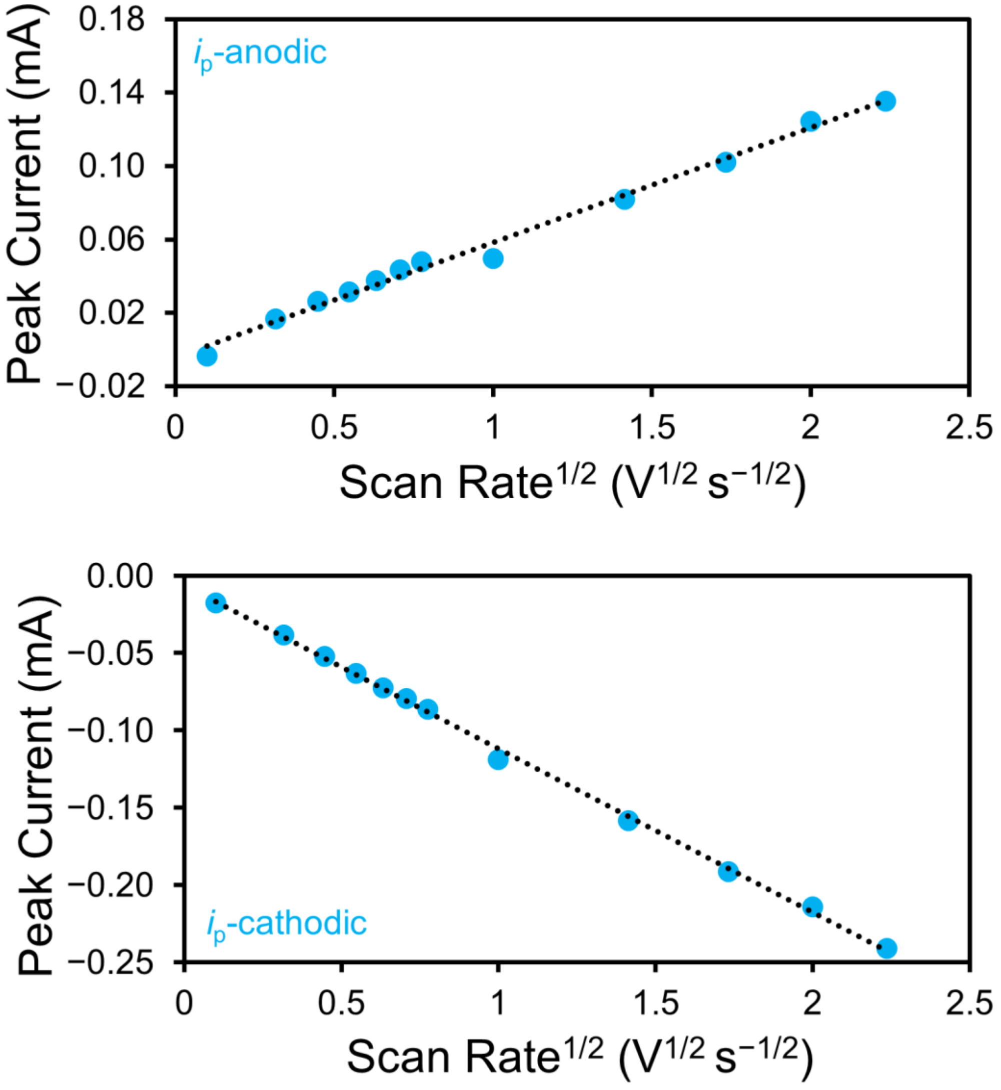

Figure S37. Randles–Ševčík analysis of the R3 redox couple of $(TBA)_3(PW_{12})$ in MeCN containing 100 mM of $LiPF_6$ supporting electrolyte at room temperature (~19–21 °C), obtained from variable-scan-rate CV data (Figure S34). i_p-anodic and i_p-cathodic denote the anodic and cathodic peak currents, respectively. Blue circles denote experimental data, and the black dotted lines represent linear fits to the data. A temperature of 20 °C was used for analysis.

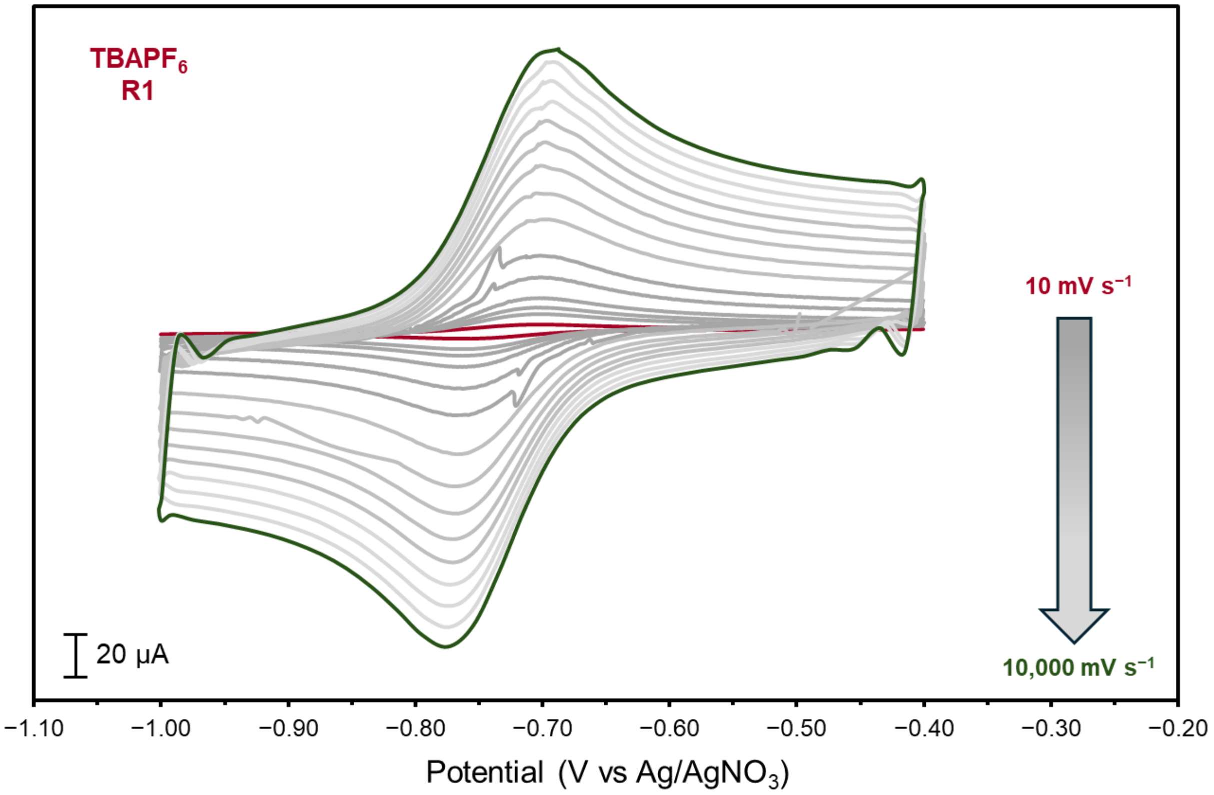

Figure S38. CVs of 1 mM of $(TBA)_3(PW_{12})$ in DMF containing 100 mM of $TBAPF_6$ supporting electrolyte collected at room temperature (~19–21 °C) at variable scan rates (10–10,000 mV s^{-1}). The R1 redox couple is displayed. Observed spikes in the data are the results of instrumental artifacts at fast scan rates.

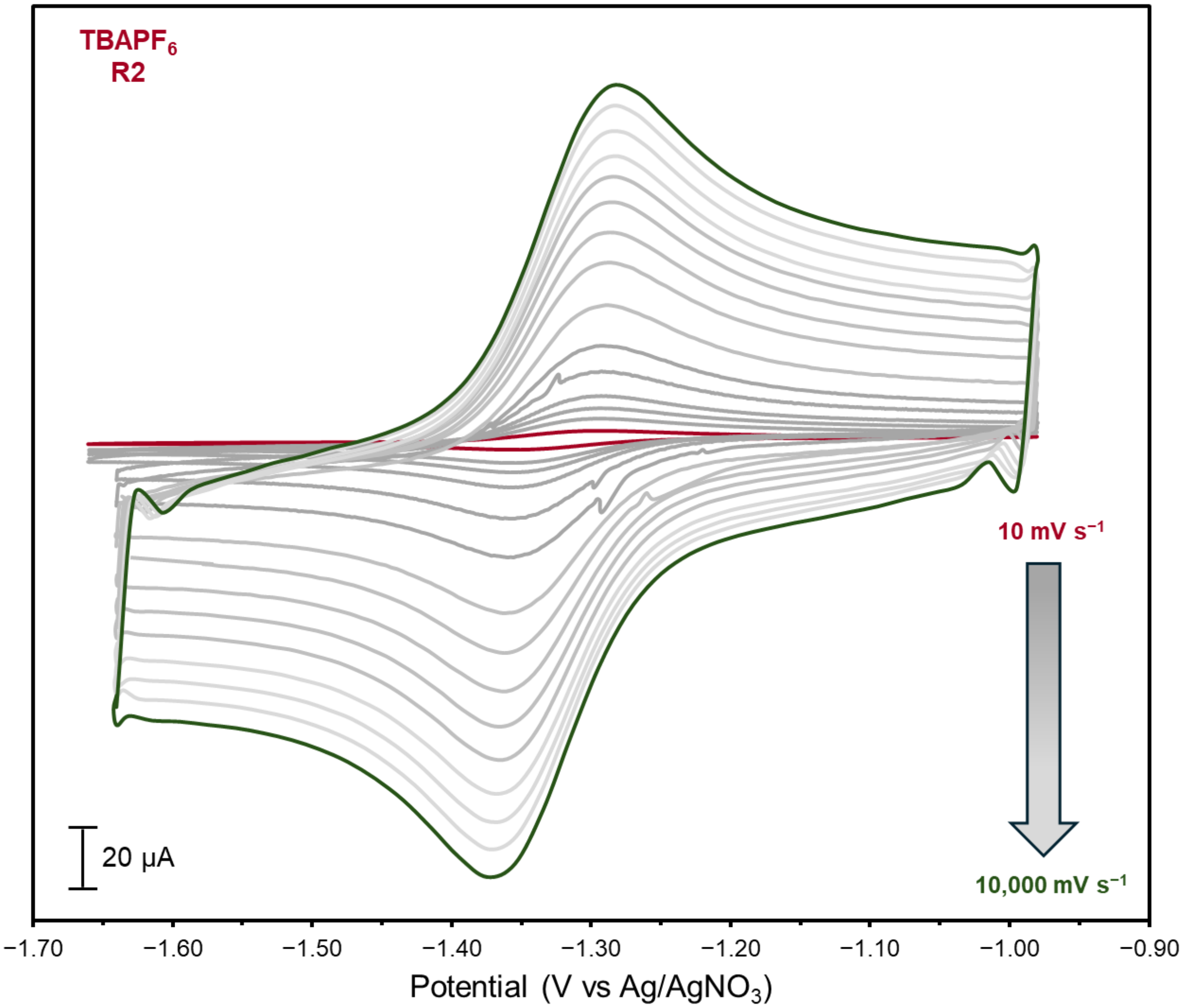

Figure S39. CVs of 1 mM of $(TBA)_3(PW_{12})$ in DMF containing 100 mM of $TBAPF_6$ supporting electrolyte collected at room temperature (~19–21 °C) at variable scan rates (10–10,000 mV s^{-1}). The R2 redox couple is displayed. Observed spikes in the data are the results of instrumental artifacts at fast scan rates.

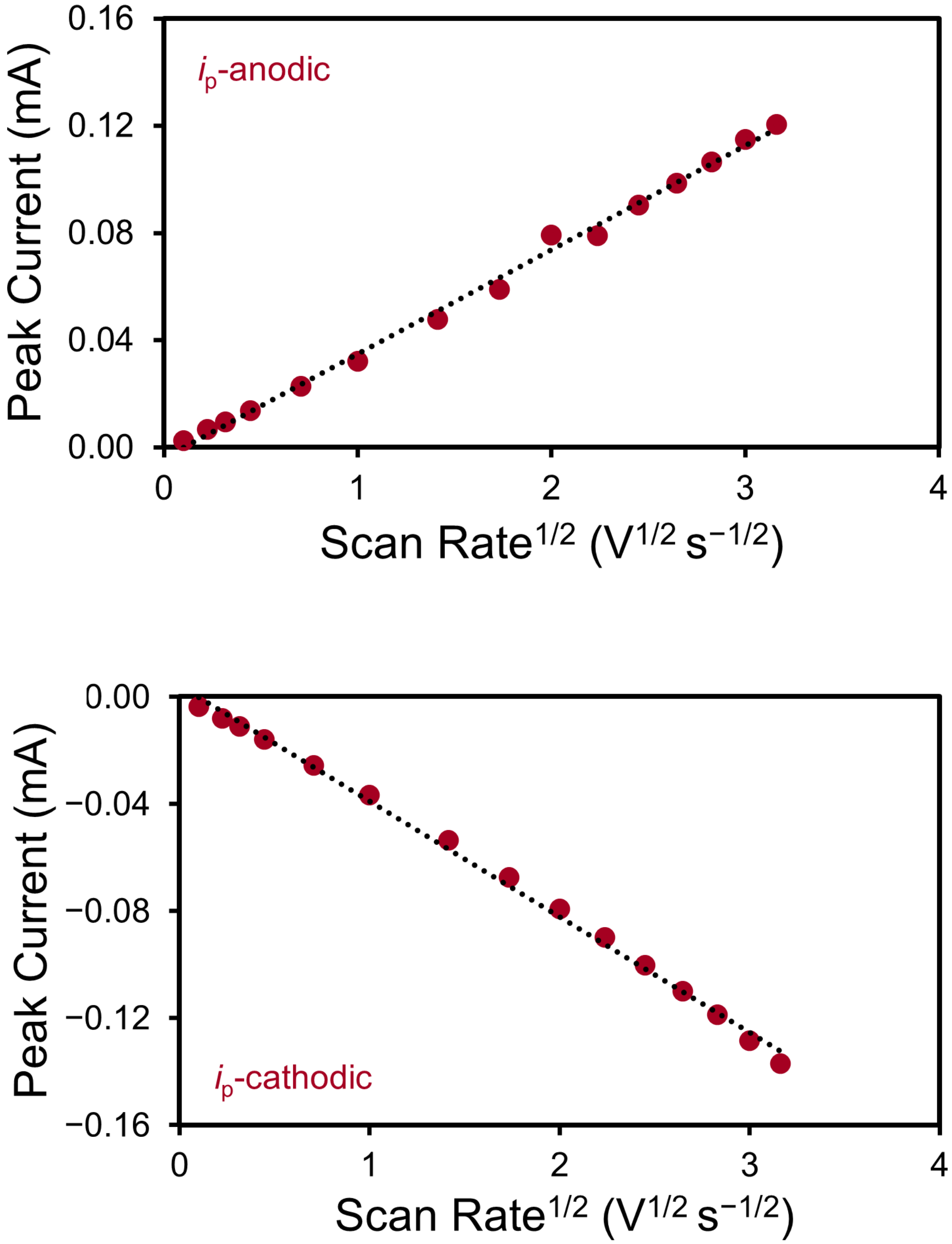

Figure S40. Randles–Ševčík analysis of the R1 redox couple of $(TBA)_3(PW_{12})$ in DMF containing 100 mM of $TBAPF_6$ supporting electrolyte at room temperature (~19–21 °C), obtained from variable-scan-rate CV data (Figure S38). i_p-anodic and i_p-cathodic denote the anodic and cathodic peak currents, respectively. Red circles denote experimental data, and the black dotted lines represent linear fits to the data. A temperature of 20 °C was used for analysis.

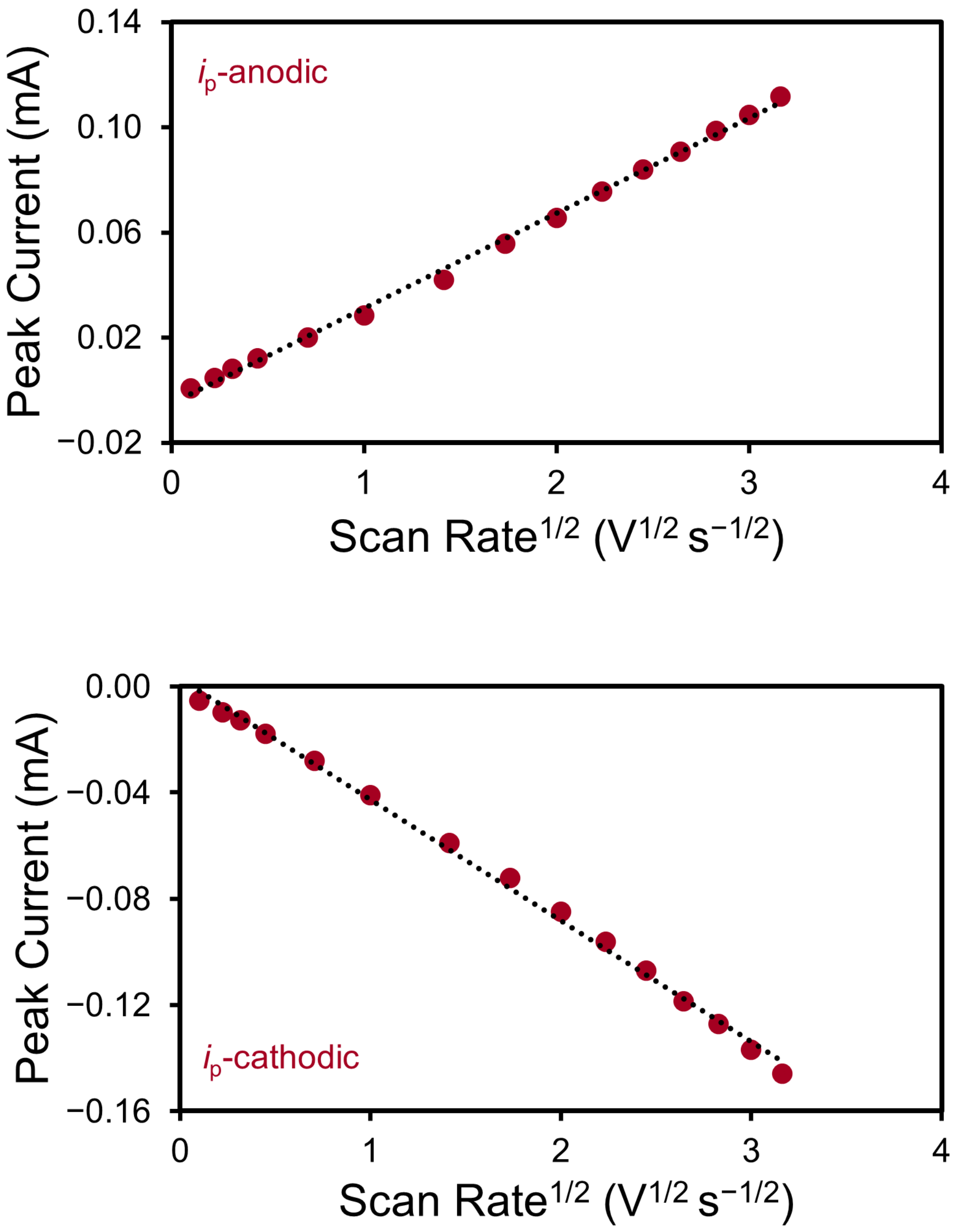

Figure S41. Randles–Ševčík analysis of the R2 redox couple of $(TBA)_3(PW_{12})$ in DMF containing 100 mM of $TBAPF_6$ supporting electrolyte at room temperature (~19–21 °C), obtained from variable-scan-rate CV data (Figure S39). i_p-anodic and i_p-cathodic denote the anodic and cathodic peak currents, respectively. Red circles denote experimental data, and the black dotted lines represent linear fits to the data. A temperature of 20 °C was used for analysis.

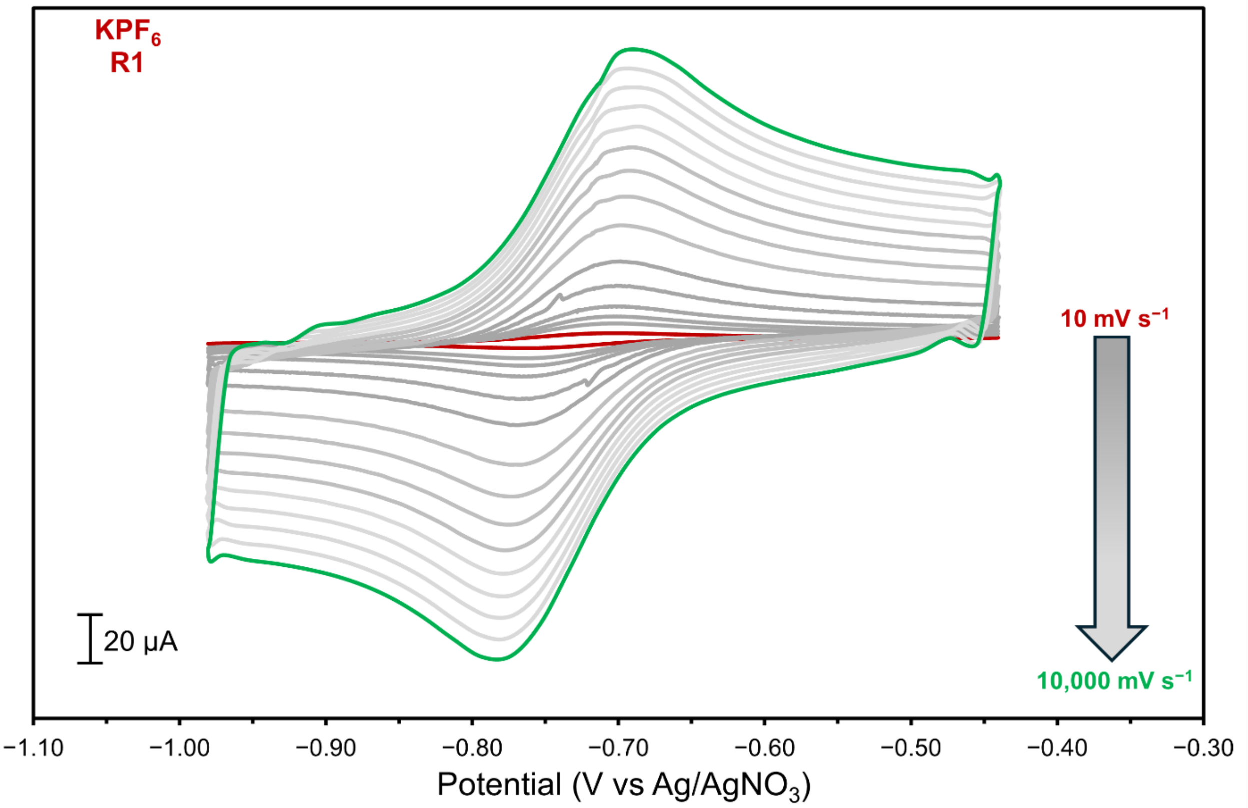

Figure S42. CVs of 1 mM of $(TBA)_3(PW_{12})$ in DMF containing 100 mM of KPF_6 supporting electrolyte collected at room temperature (~19–21 °C) at variable scan rates (10–10,000 mV s^{-1}). The R1 redox couple is displayed. Observed spikes in the data are the results of instrumental artifacts at fast scan rates.

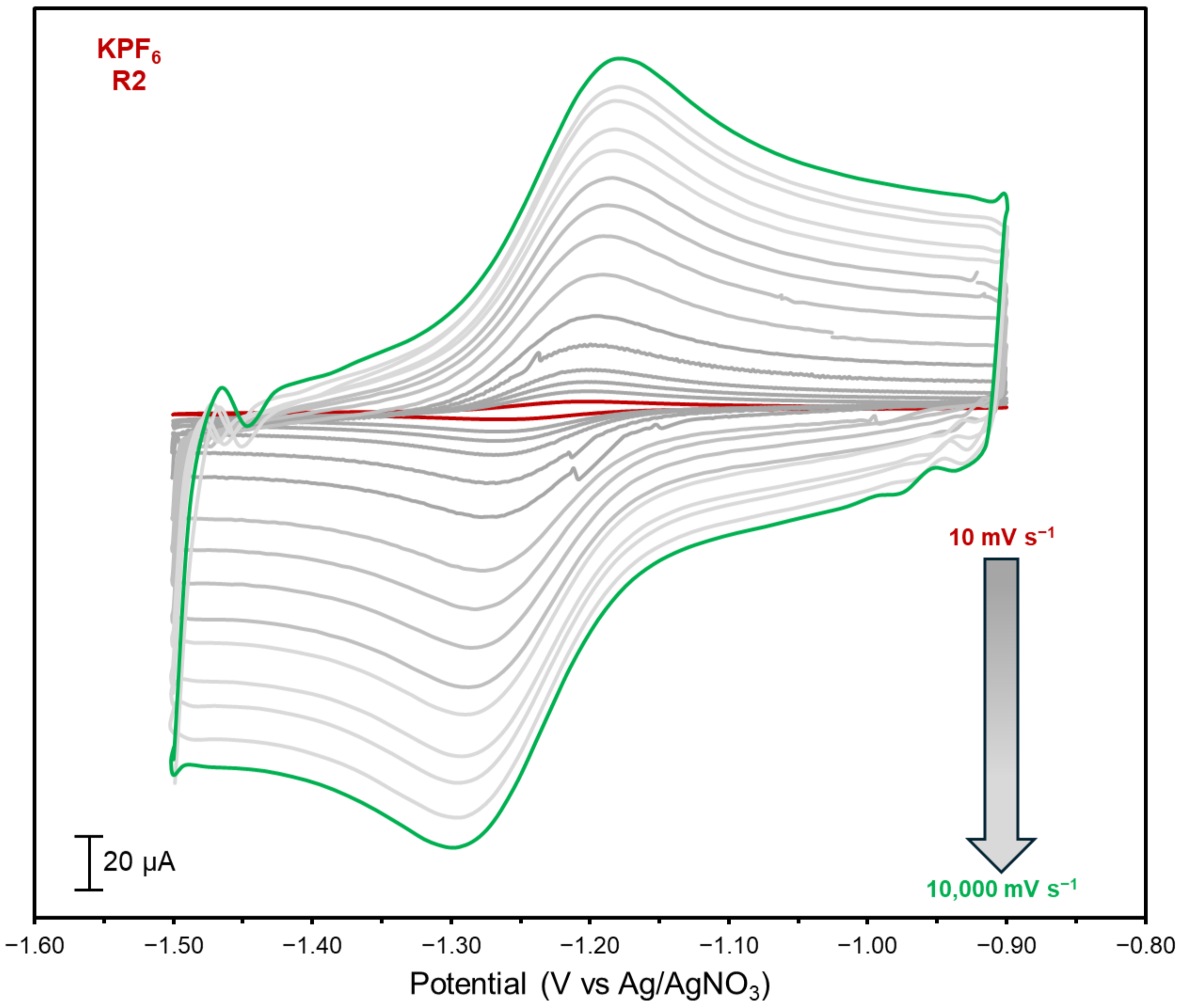

Figure S43. CVs of 1 mM of $(TBA)_3(PW_{12})$ in DMF containing 100 mM of KPF_6 supporting electrolyte collected at room temperature (~19–21 °C) at variable scan rates (10–10,000 mV s^{-1}). The R2 redox couple is displayed. Observed spikes in the data are the results of instrumental artifacts at fast scan rates.

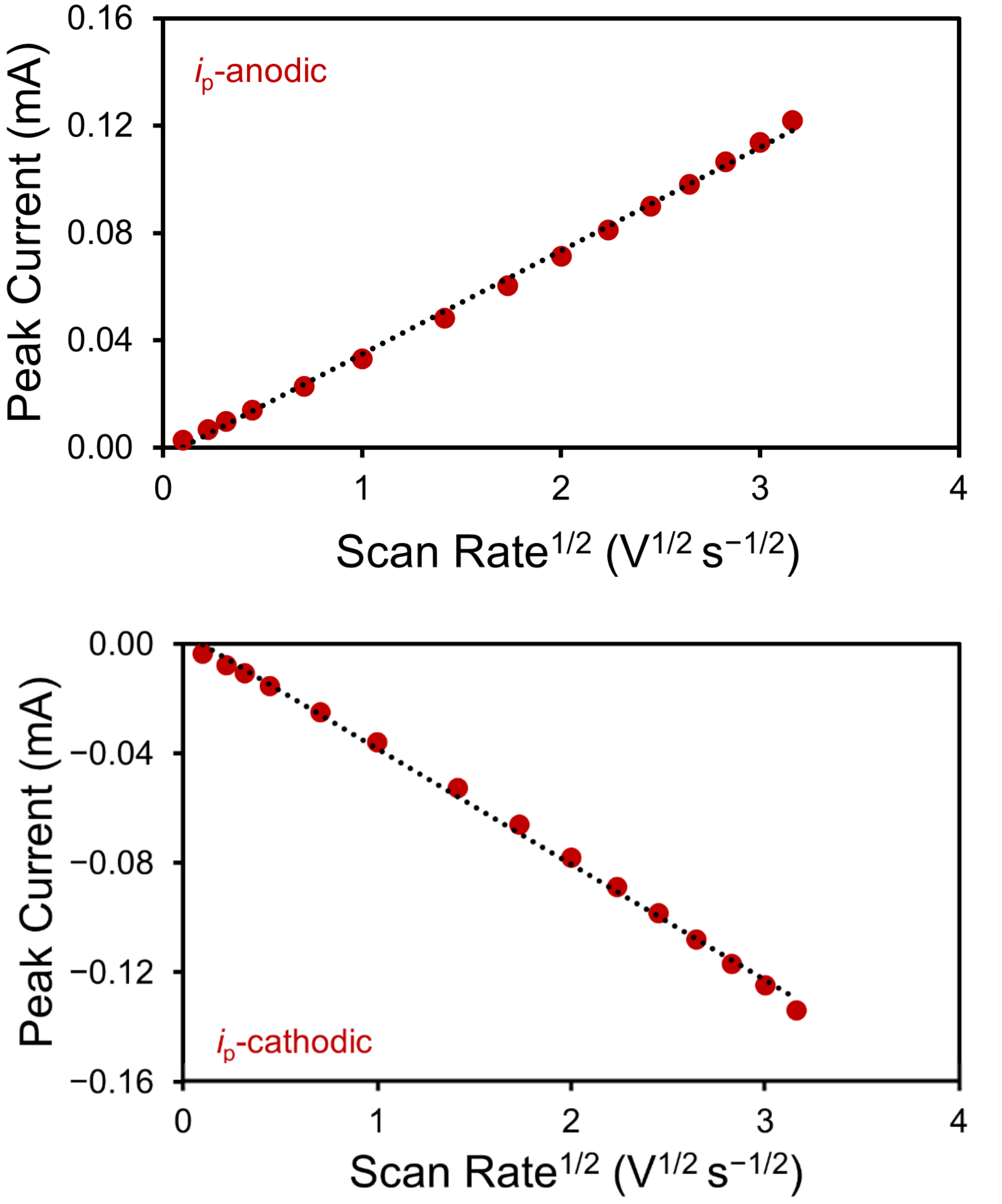

Figure S44. Randles–Ševčík analysis of the R1 redox couple of $(TBA)_3(PW_{12})$ in DMF containing 100 mM of KPF_6 supporting electrolyte at room temperature (~19–21 °C), obtained from variable-scan-rate CV data (Figure S42). i_p-anodic and i_p-cathodic denote the anodic and cathodic peak currents, respectively. Red circles denote experimental data, and the black dotted lines represent linear fits to the data. A temperature of 20 °C was used for analysis.

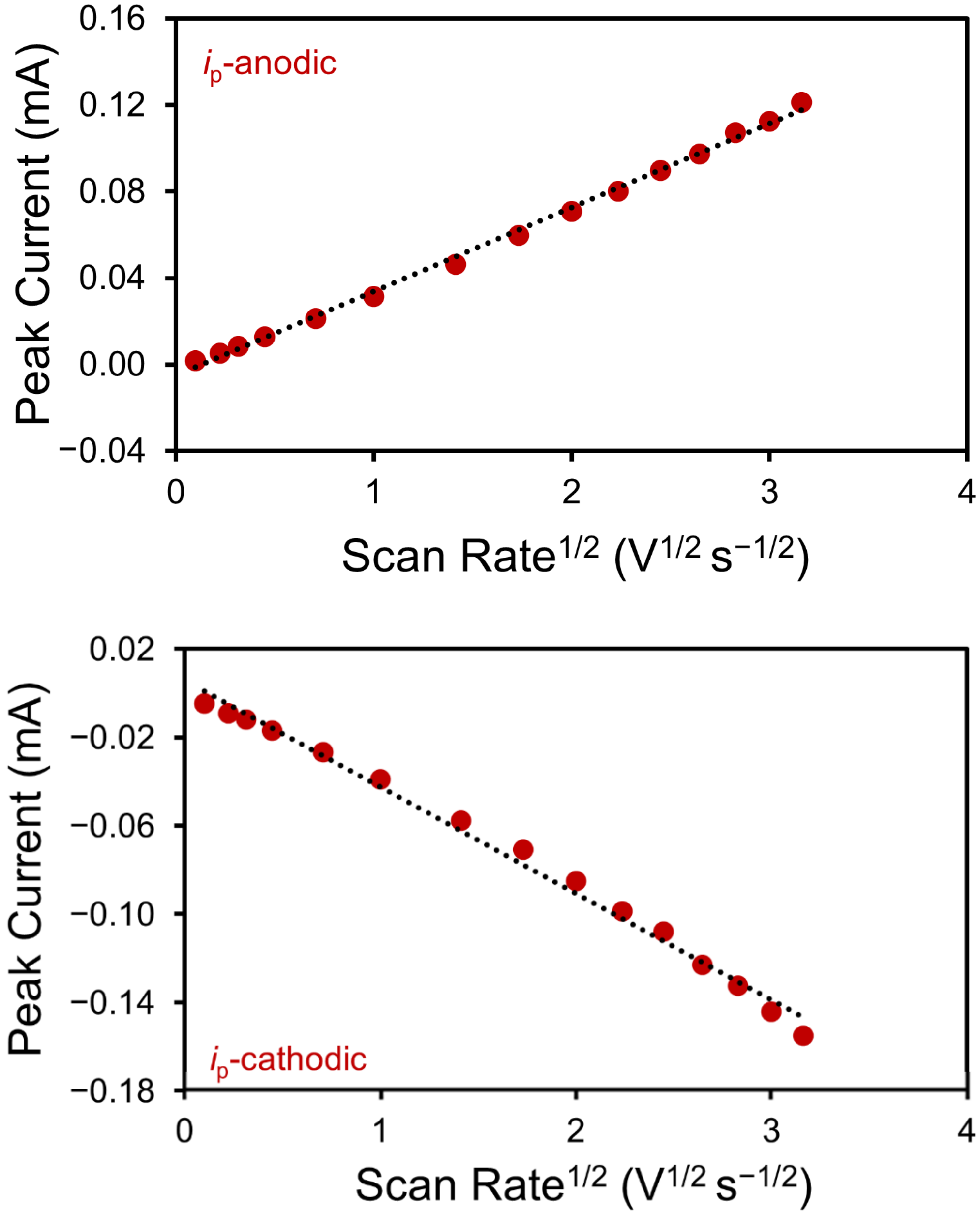

Figure S45. Randles–Ševčík analysis of the R2 redox couple of $(TBA)_3(PW_{12})$ in DMF containing 100 mM of KPF_6 supporting electrolyte at room temperature (~19–21 °C), obtained from variable-scan-rate CV data (Figure S43). i_p-anodic and i_p-cathodic denote the anodic and cathodic peak currents, respectively. Red circles denote experimental data, and the black dotted lines represent linear fits to the data. A temperature of 20 °C was used for analysis.

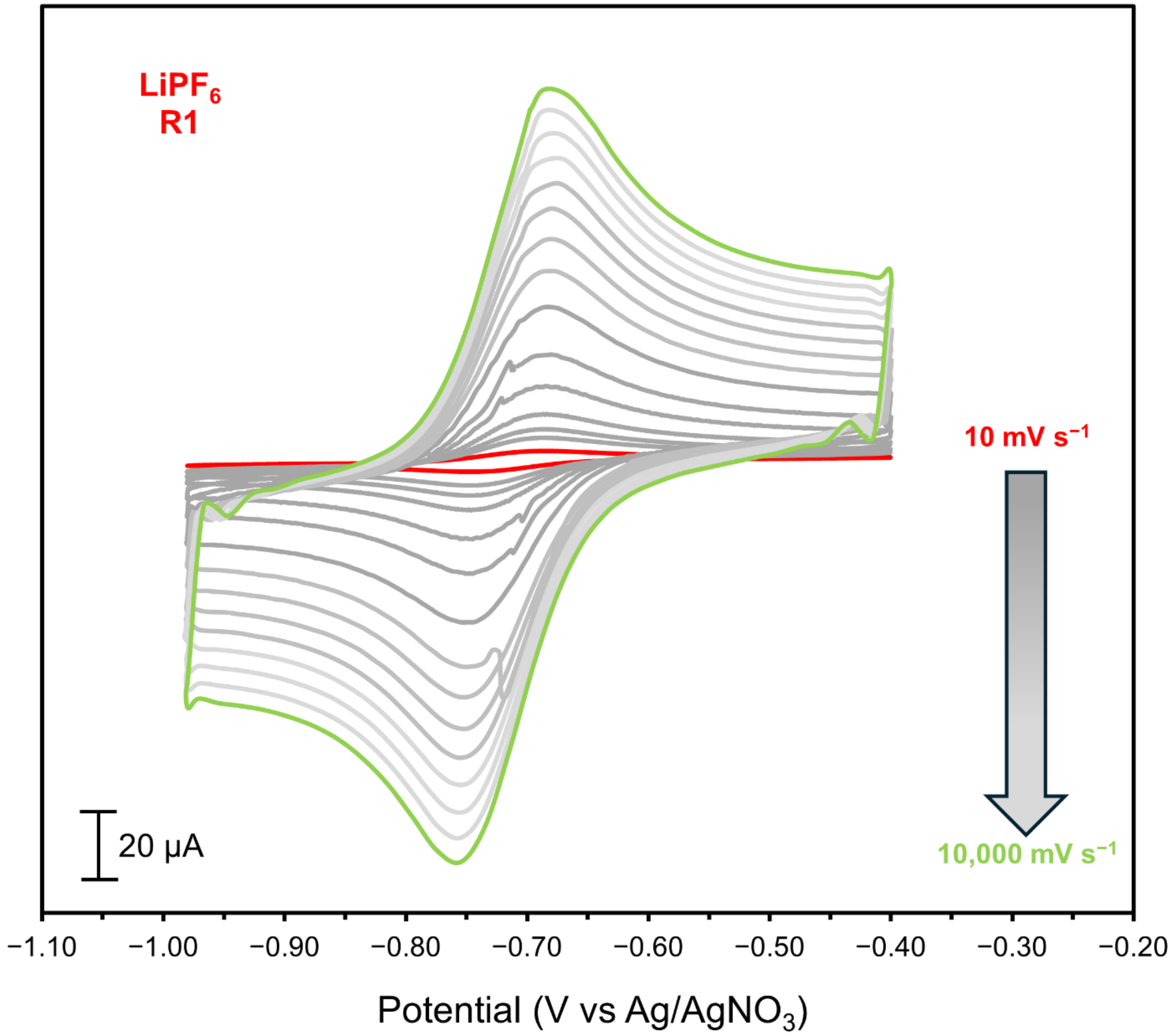

Figure S46. CVs of 1 mM of $(TBA)_3(PW_{12})$ in DMF containing 100 mM of $LiPF_6$ supporting electrolyte collected at room temperature (~19–21 °C) at variable scan rates (10–10,000 mV s^{-1}). The R1 redox couple is displayed. Observed spikes in the data are the results of instrumental artifacts at fast scan rates.

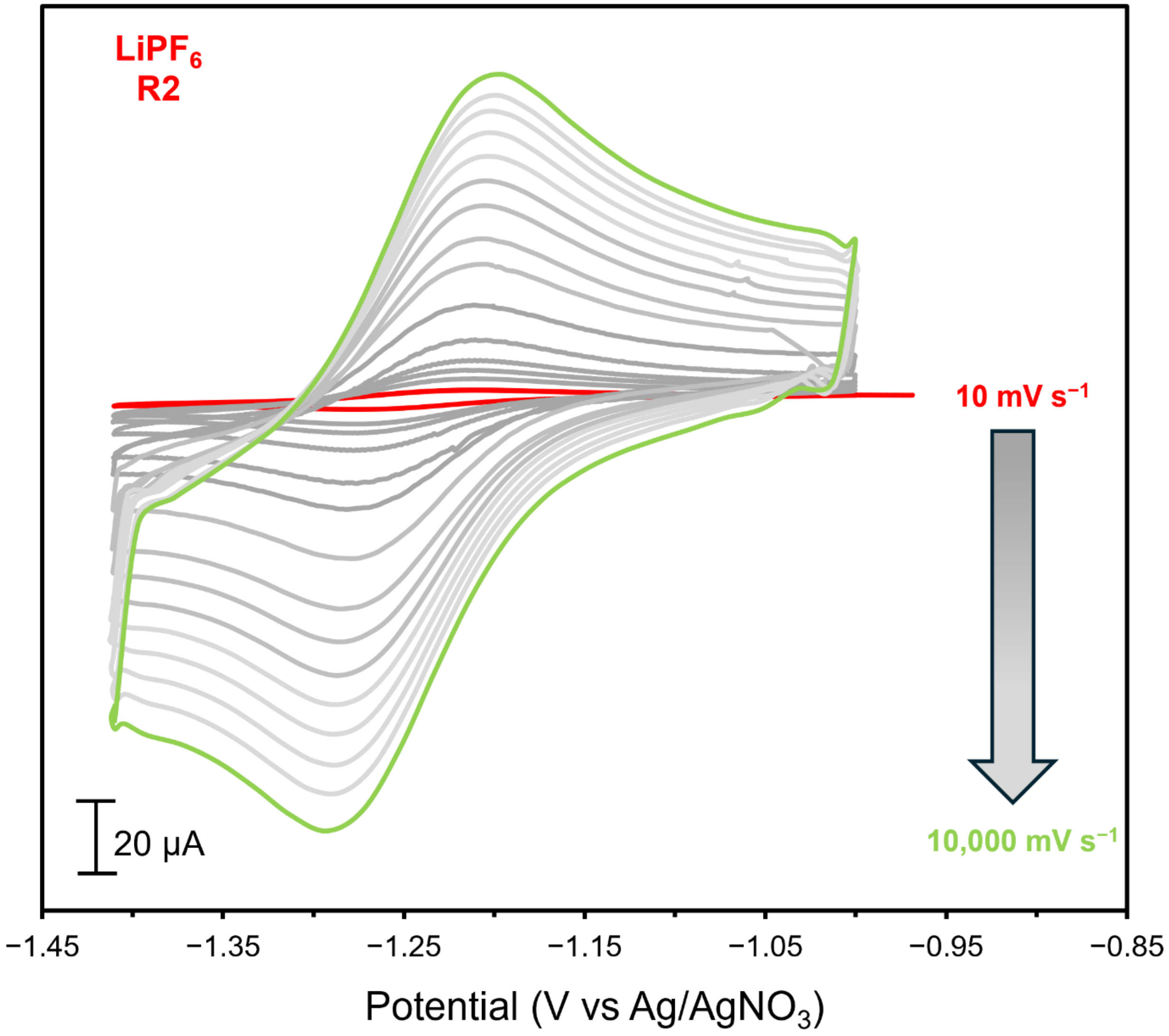

Figure S47. CVs of 1 mM of $(TBA)_3(PW_{12})$ in DMF containing 100 mM of $LiPF_6$ supporting electrolyte collected at room temperature (~19–21 °C) at variable scan rates (10–10,000 mV s^{-1}). The R2 redox couple is displayed. Observed spikes in the data are the results of instrumental artifacts at fast scan rates.

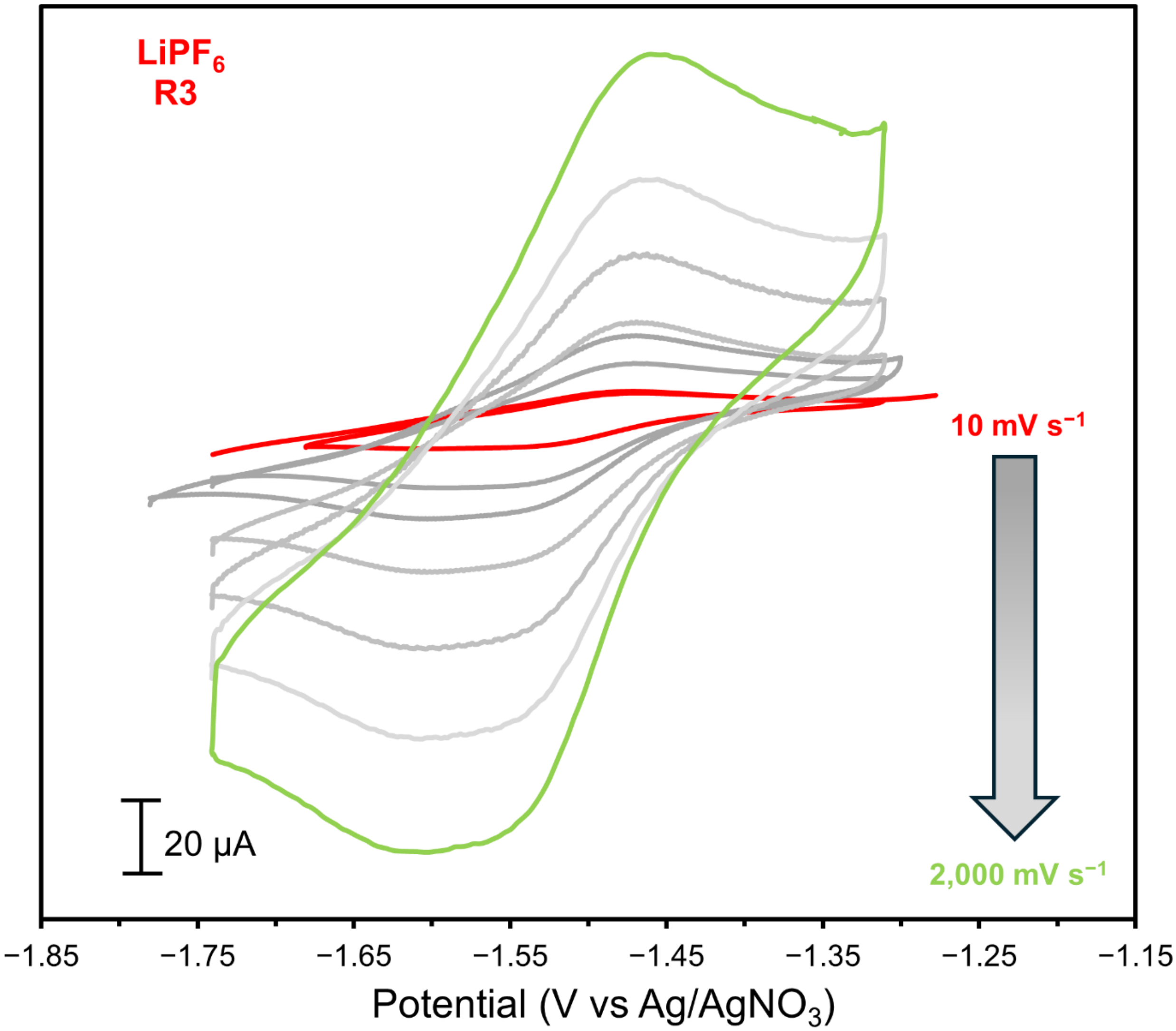

Figure S48. CVs of 1 mM of $(TBA)_3(PW_{12})$ in DMF containing 100 mM of $LiPF_6$ supporting electrolyte collected at room temperature (~19–21 °C) at variable scan rates (10–10,000 mV s^{-1}). The R3 redox couple is displayed. Observed spikes in the data are the results of instrumental artifacts at fast scan rates.

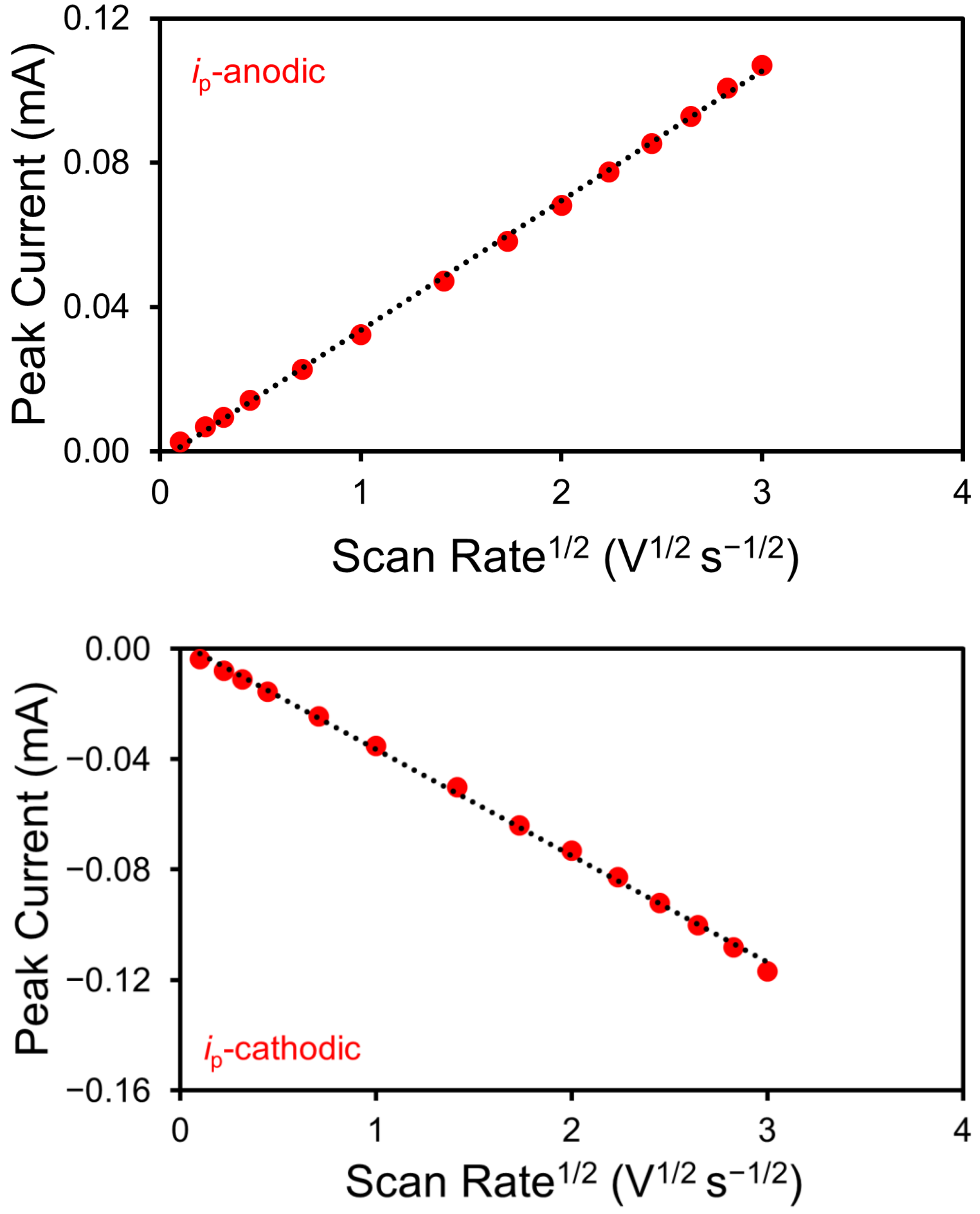

Figure S49. Randles–Ševčík analysis of the R1 redox couple of $(TBA)_3(PW_{12})$ in DMF containing 100 mM of $LiPF_6$ supporting electrolyte at room temperature (~19–21 °C), obtained from variable-scan-rate CV data (Figure S46). i_p-anodic and i_p-cathodic denote the anodic and cathodic peak currents, respectively. Red circles denote experimental data, and the black dotted lines represent linear fits to the data. A temperature of 20 °C was used for analysis.

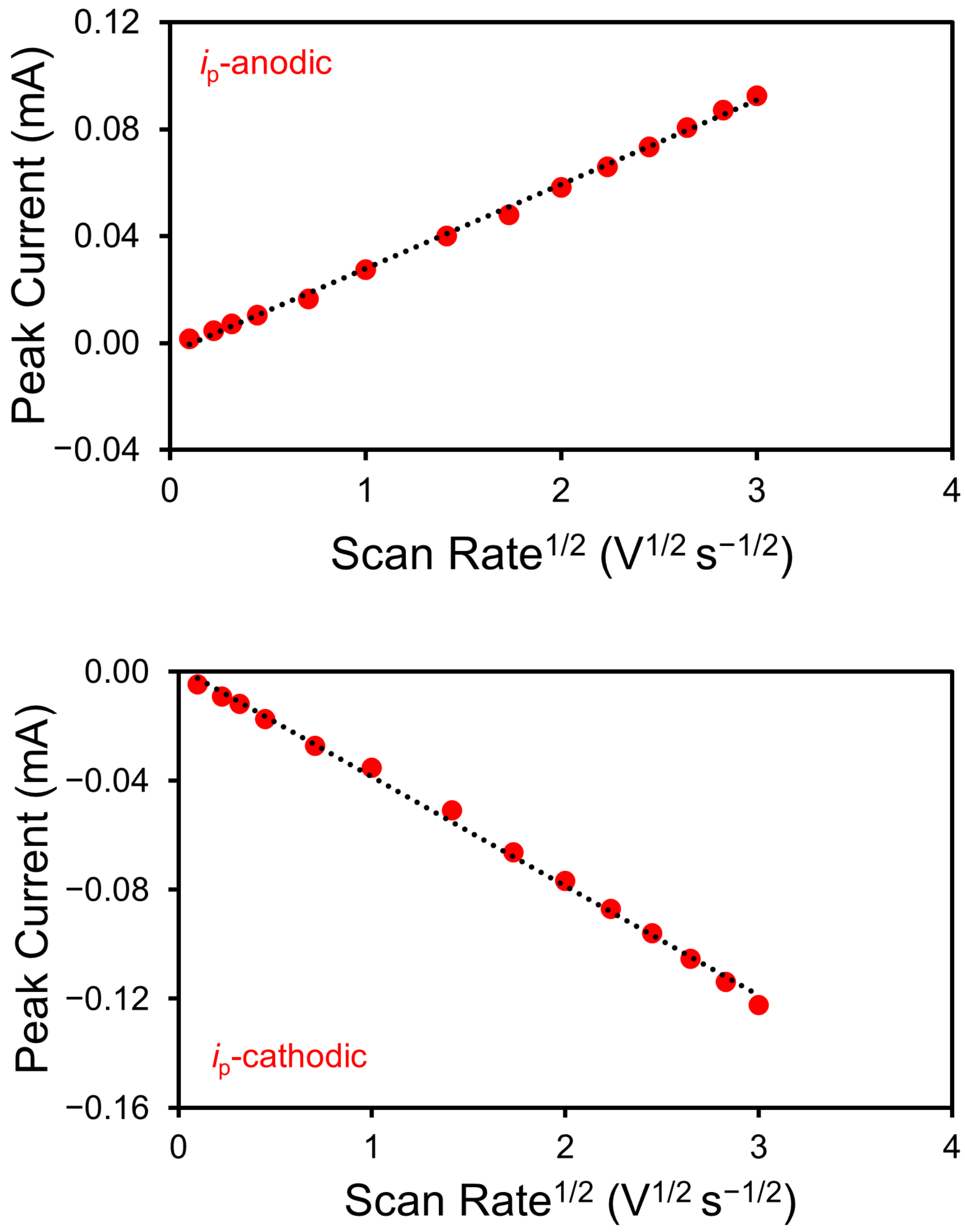

Figure S50. Randles–Ševčík analysis of the R2 redox couple of $(TBA)_3(PW_{12})$ in DMF containing 100 mM of $LiPF_6$ supporting electrolyte at room temperature (~19–21 °C), obtained from variable-scan-rate CV data (Figure S47). i_p-anodic and i_p-cathodic denote the anodic and cathodic peak currents, respectively. Red circles denote experimental data, and the black dotted lines represent linear fits to the data. A temperature of 20 °C was used for analysis.

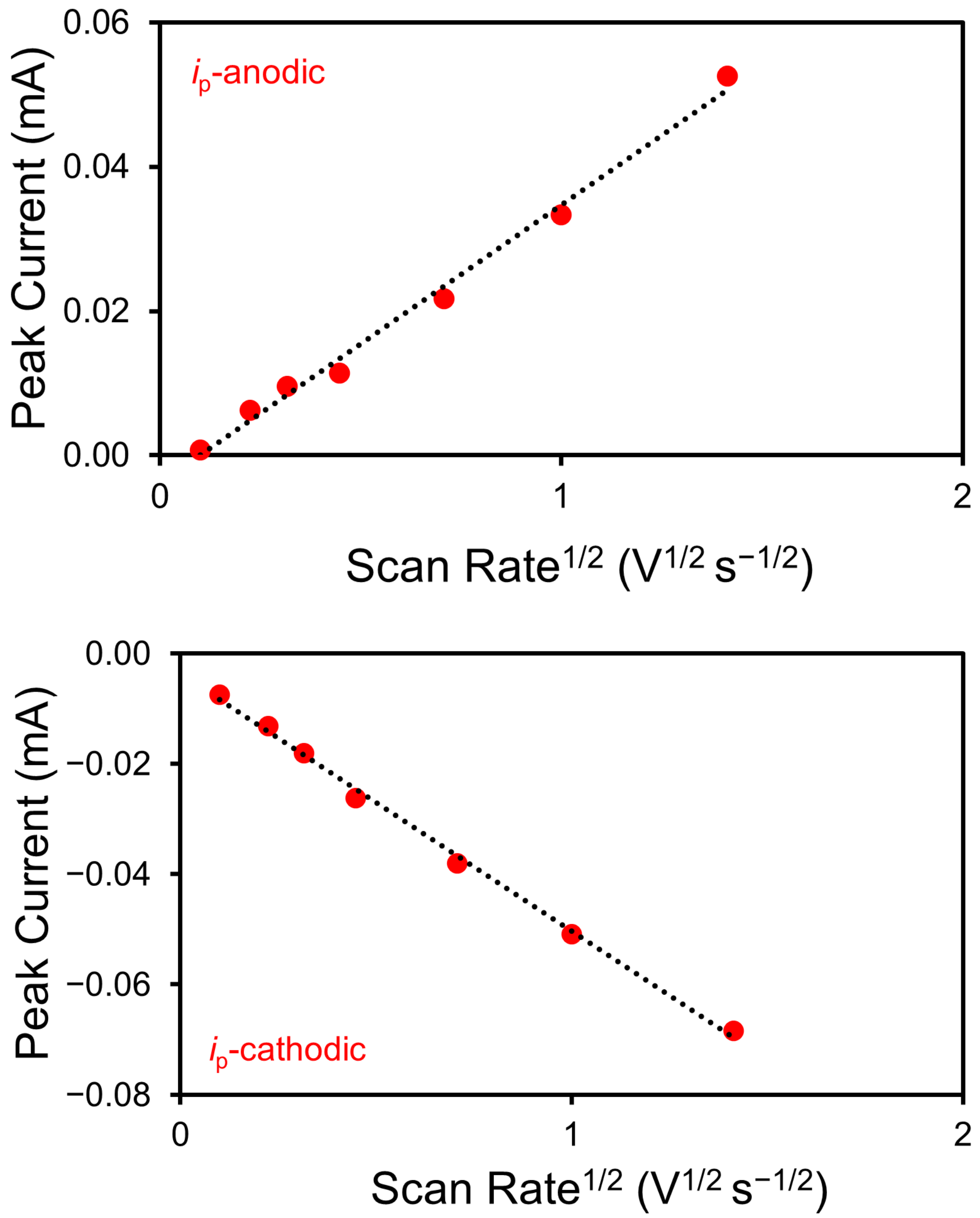

Figure S51. Randles–Ševčík analysis of the R3 redox couple of $(TBA)_3(PW_{12})$ in DMF containing 100 mM of $LiPF_6$ supporting electrolyte at room temperature (~19–21 °C), obtained from variable-scan-rate CV data (Figure S48). i_p-anodic and i_p-cathodic denote the anodic and cathodic peak currents, respectively. Red circles denote experimental data, and the black dotted lines represent linear fits to the data. A temperature of 20 °C was used for analysis.

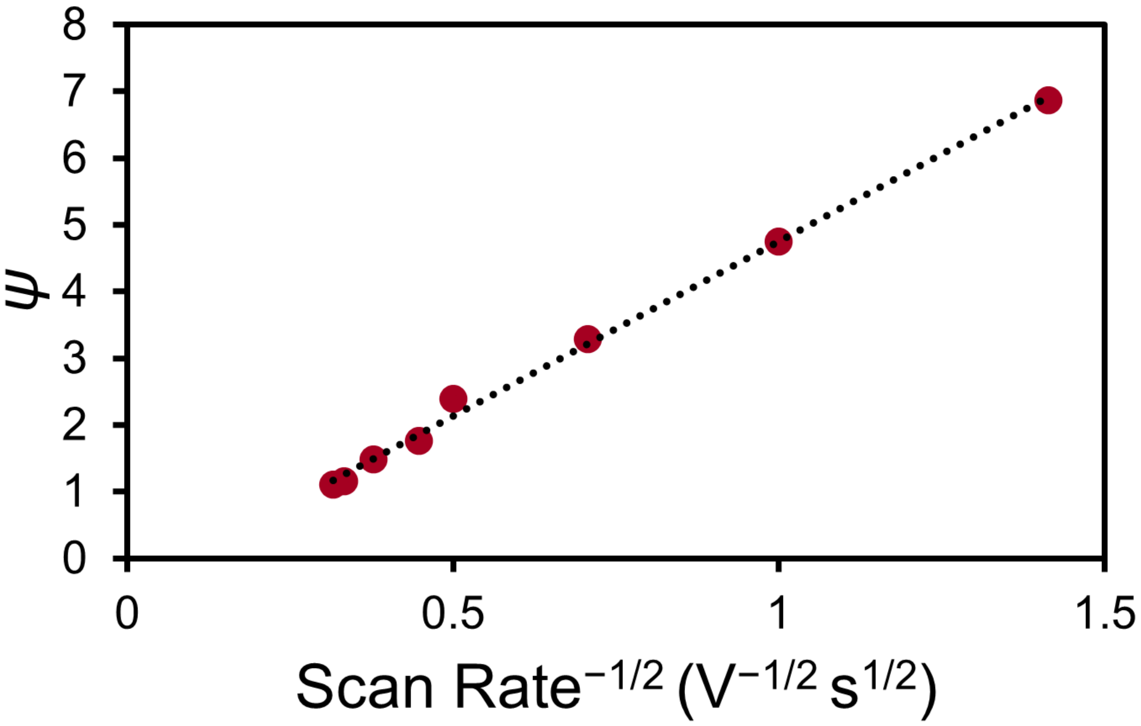

Figure S52. Nicholson plot of ψ vs inverse square root of scan rate for the R1 redox couple of $(TBA)_3(PW_{12})$ in DMF containing 100 mM of $TBAPF_6$ supporting electrolyte at room temperature (~19–21 °C), obtained from variable-scan-rate CV data (Figure S38). Red circles denote experimental data, and the black dotted line represents a linear fit to the data. A temperature of 20 °C was used for analysis.

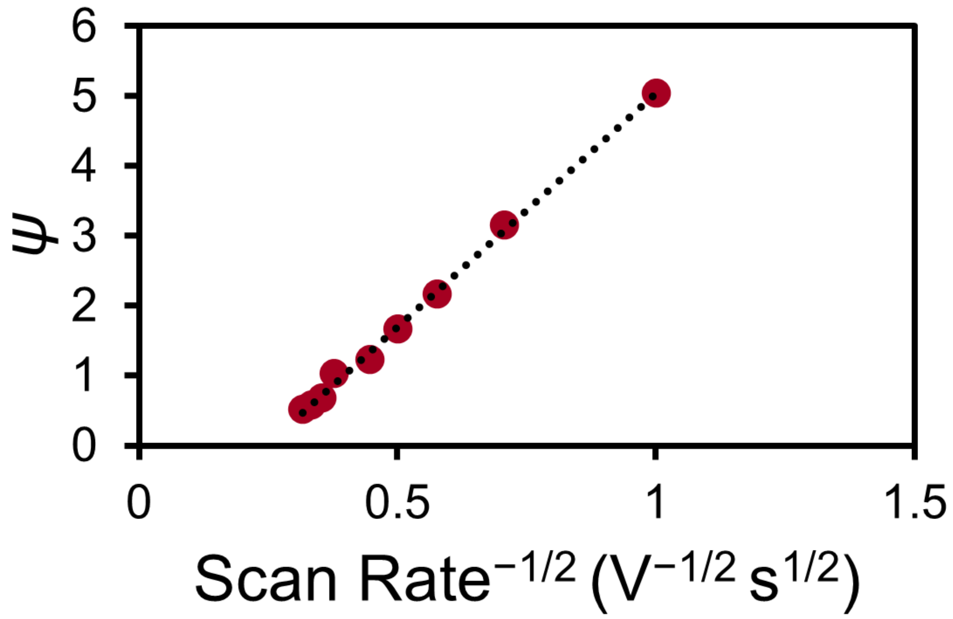

Figure S53. Nicholson plot of ψ vs inverse square root of scan rate for the R2 redox couple of $(TBA)_3(PW_{12})$ in DMF containing 100 mM of $TBAPF_6$ supporting electrolyte at room temperature (~19–21 °C), obtained from variable-scan-rate CV data (Figure S39). Red circles denote experimental data, and the black dotted line represents a linear fit to the data. A temperature of 20 °C was used for analysis.

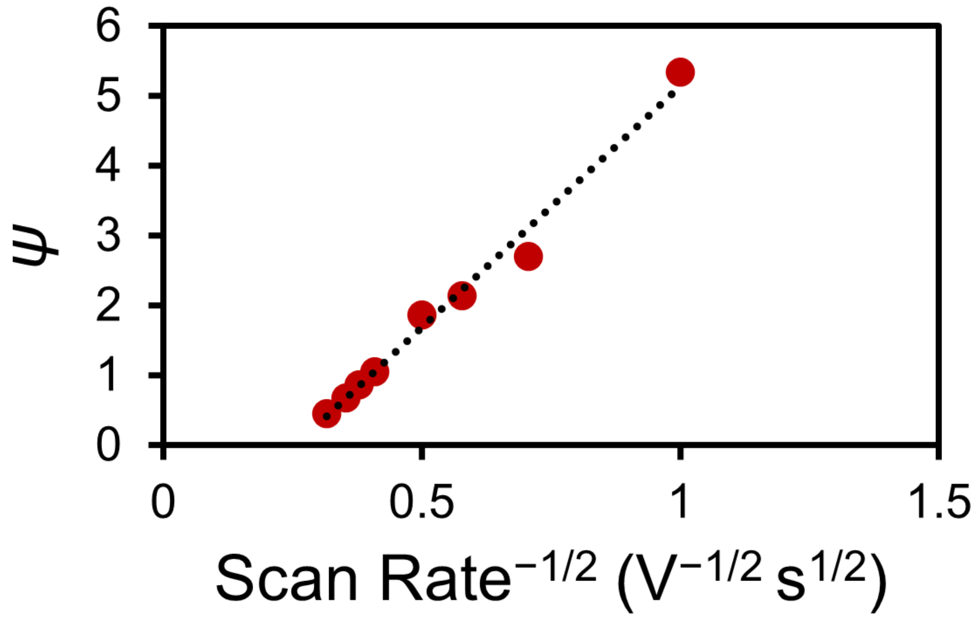

Figure S54. Nicholson plot of ψ vs inverse square root of scan rate for the R1 redox couple of $(TBA)_3(PW_{12})$ in DMF containing 100 mM of KPF_6 supporting electrolyte at room temperature (~19–21 °C), obtained from variable-scan-rate CV data (Figure S42). Red circles denote experimental data, and the black dotted line represents a linear fit to the data. A temperature of 20 °C was used for analysis.

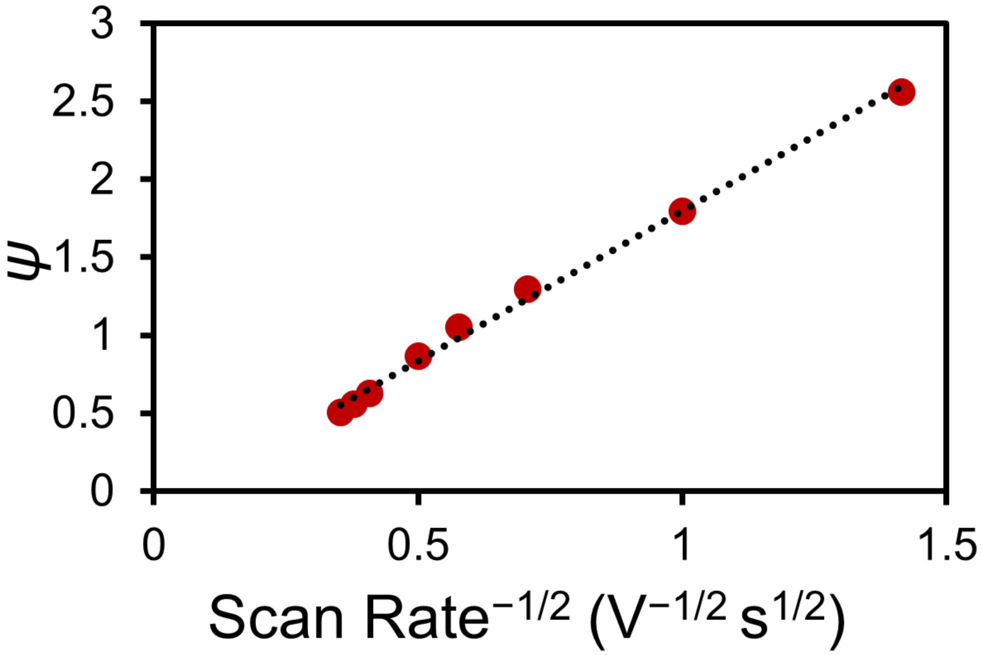

Figure S55. Nicholson plot of *ψ* vs inverse square root of scan rate for the R2 redox couple of $(TBA)_3(PW_{12})$ in DMF containing 100 mM of KPF_6 supporting electrolyte at room temperature (~19–21 °C), obtained from variable-scan-rate CV data (Figure S43). Red circles denote experimental data, and the black dotted line represents a linear fit to the data. A temperature of 20 °C was used for analysis.

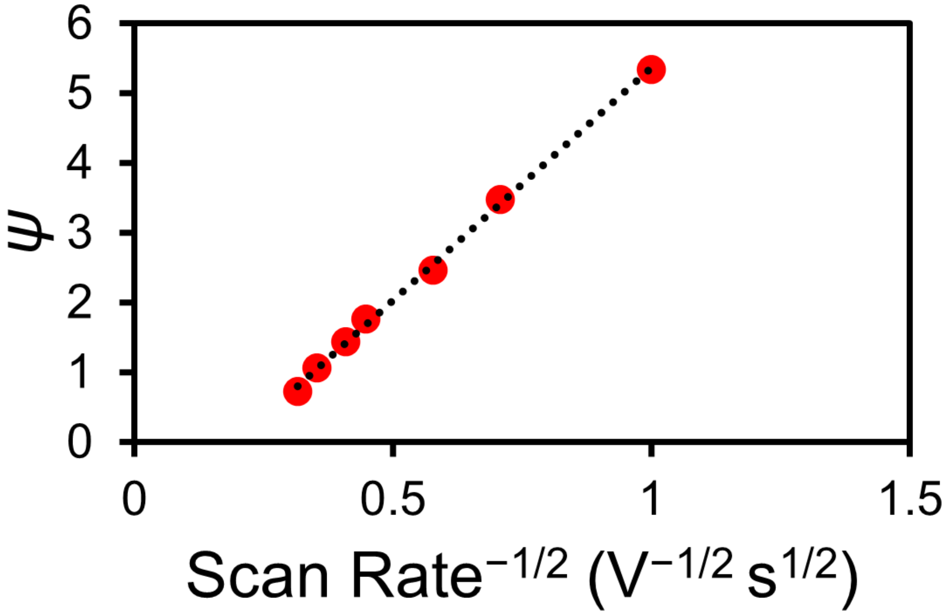

Figure S56. Nicholson plot of ψ vs inverse square root of scan rate for the R1 redox couple of $(TBA)_3(PW_{12})$ in DMF containing 100 mM of $LiPF_6$ supporting electrolyte at room temperature (~19–21 °C), obtained from variable-scan-rate CV data (Figure S46). Red circles denote experimental data, and the black dotted line represents a linear fit to the data. A temperature of 20 °C was used for analysis.

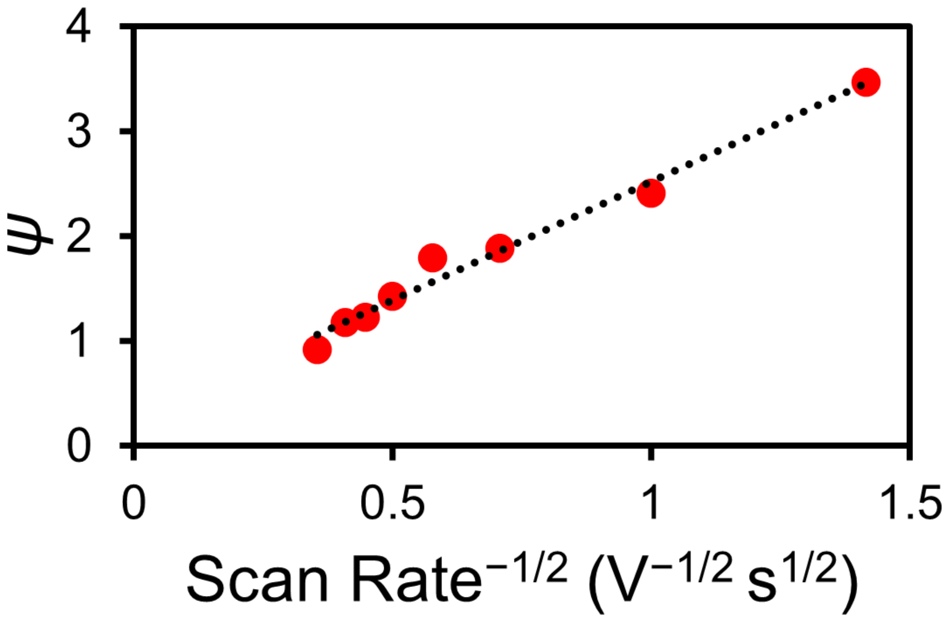

Figure S57. Nicholson plot of *ψ* vs inverse square root of scan rate for the R2 redox couple of $(TBA)_3(PW_{12})$ in DMF containing 100 mM of $LiPF_6$ supporting electrolyte at room temperature (~19–21 °C), obtained from variable-scan-rate CV data (Figure S47). Red circles denote experimental data, and the black dotted line represents a linear fit to the data. A temperature of 20 °C was used for analysis.

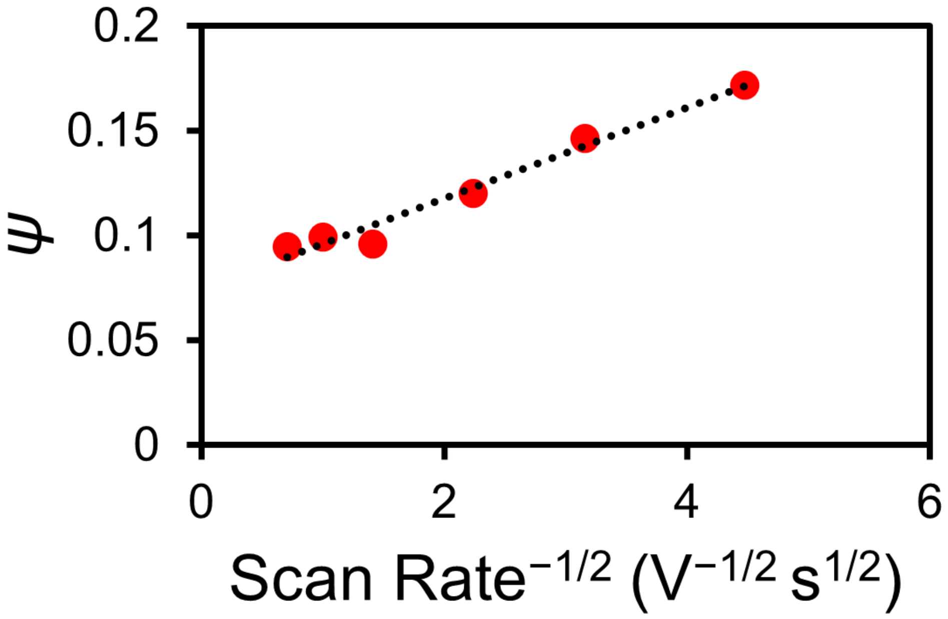

Figure S58. Nicholson plot of ψ vs inverse square root of scan rate for the R3 redox couple of $(TBA)_3(PW_{12})$ in DMF containing 100 mM of $LiPF_6$ supporting electrolyte at room temperature (~19–21 °C), obtained from variable-scan-rate CV data (Figure S48). Red circles denote experimental data, and the black dotted line represents a linear fit to the data. A temperature of 20 °C was used for analysis.

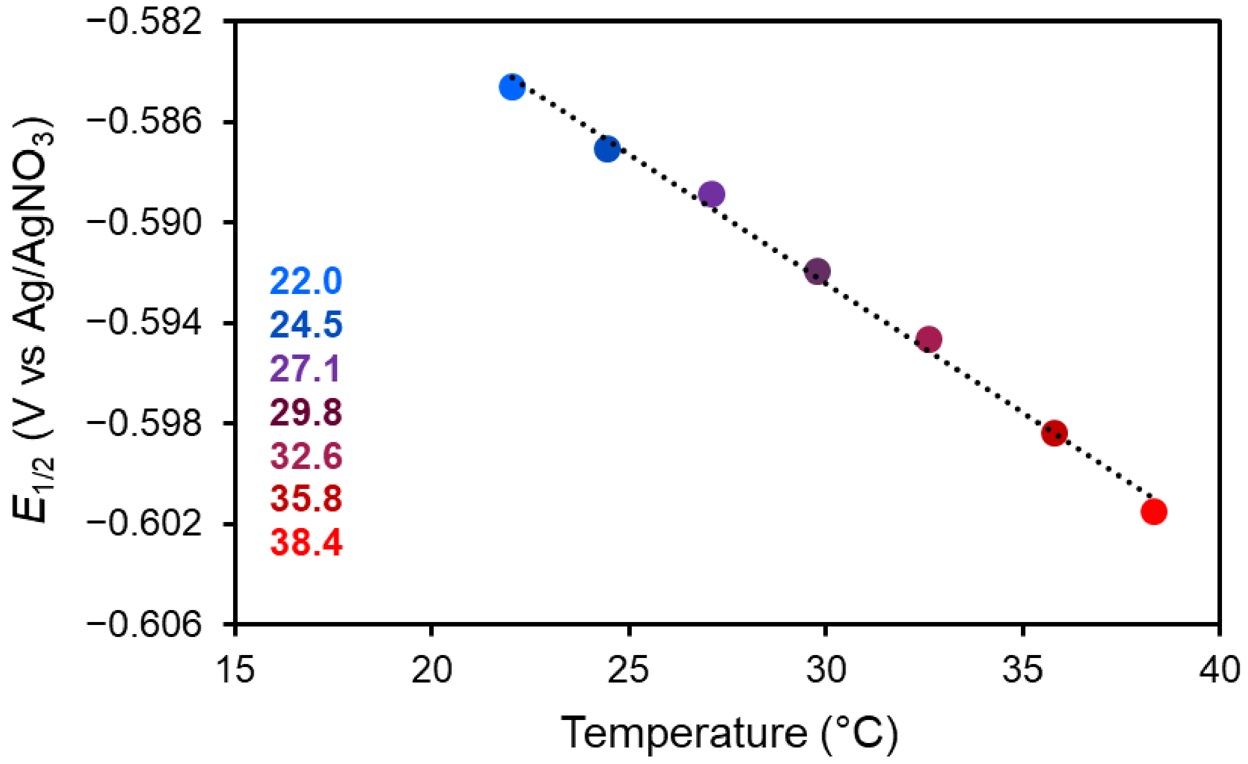

Figure S59. Plot of the temperature dependence of $E_{1/2}$ for the R1 couple of $(TBA)_3(PW_{12})$ extracted from variable-temperature CV measurements in MeCN containing 100 mM of $TBAPF_6$ supporting electrolyte (Figure 4a). Colored circles and numbers denote experimental data at their corresponding measured temperature (in °C), and the dotted black line corresponds to a linear fit to the data. The average slope from four independent measurements after correcting for the temperature coefficient of the reference electrode potential (eqs 6 and 7), which represents the average temperature coefficient, is α = −0.59(7) mV °C^{-1} (Table 1). The error in the average value corresponds to the standard deviation of independent measurements.

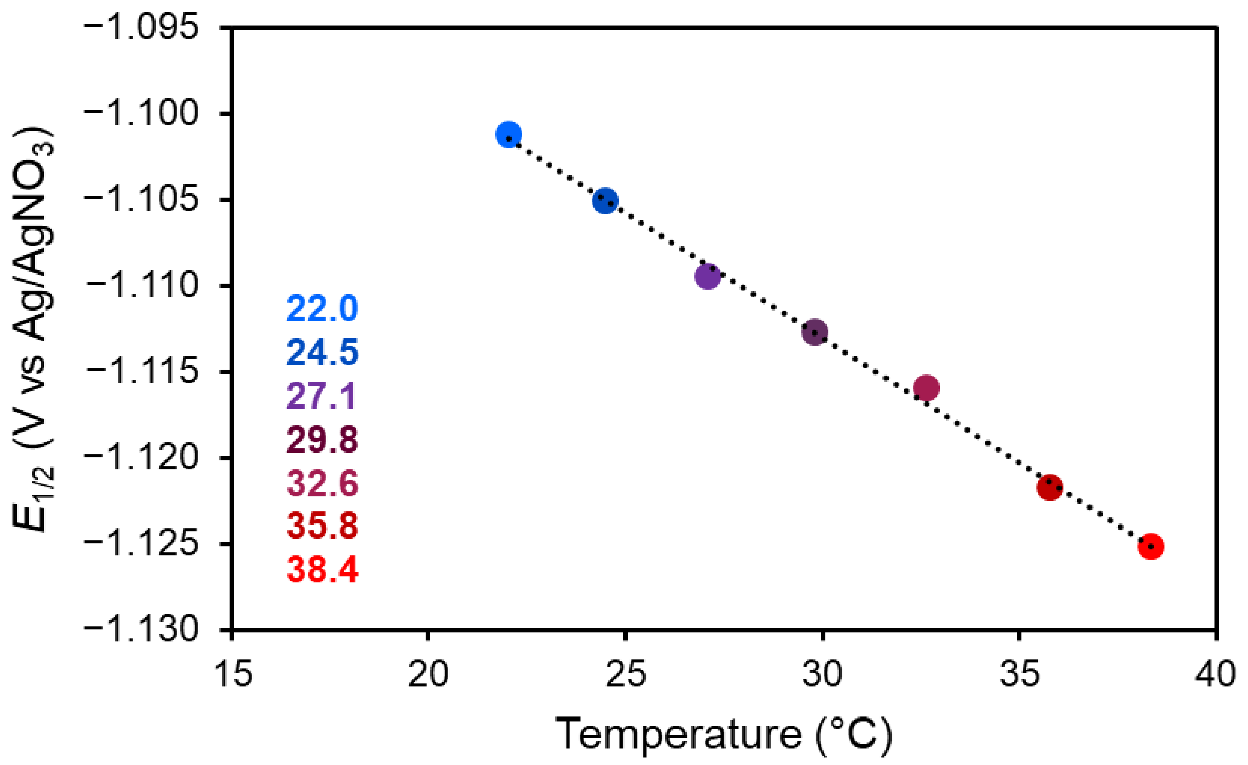

Figure S60. Plot of the temperature dependence of $E_{1/2}$ for the R2 couple of $(TBA)_3(PW_{12})$ extracted from variable-temperature CV measurements in MeCN containing 100 mM of $TBAPF_6$ supporting electrolyte (Figure 4a). Colored circles and numbers denote experimental data at their corresponding measured temperature (in °C), and the dotted black line corresponds to a linear fit to the data. The average slope from four independent measurements after correcting for the temperature coefficient of the reference electrode potential (eqs 6 and 7), which represents the average temperature coefficient, is α = −1.03(9) mV $°C^{-1}$ (Table 1). The error in the average value corresponds to the standard deviation of independent measurements.

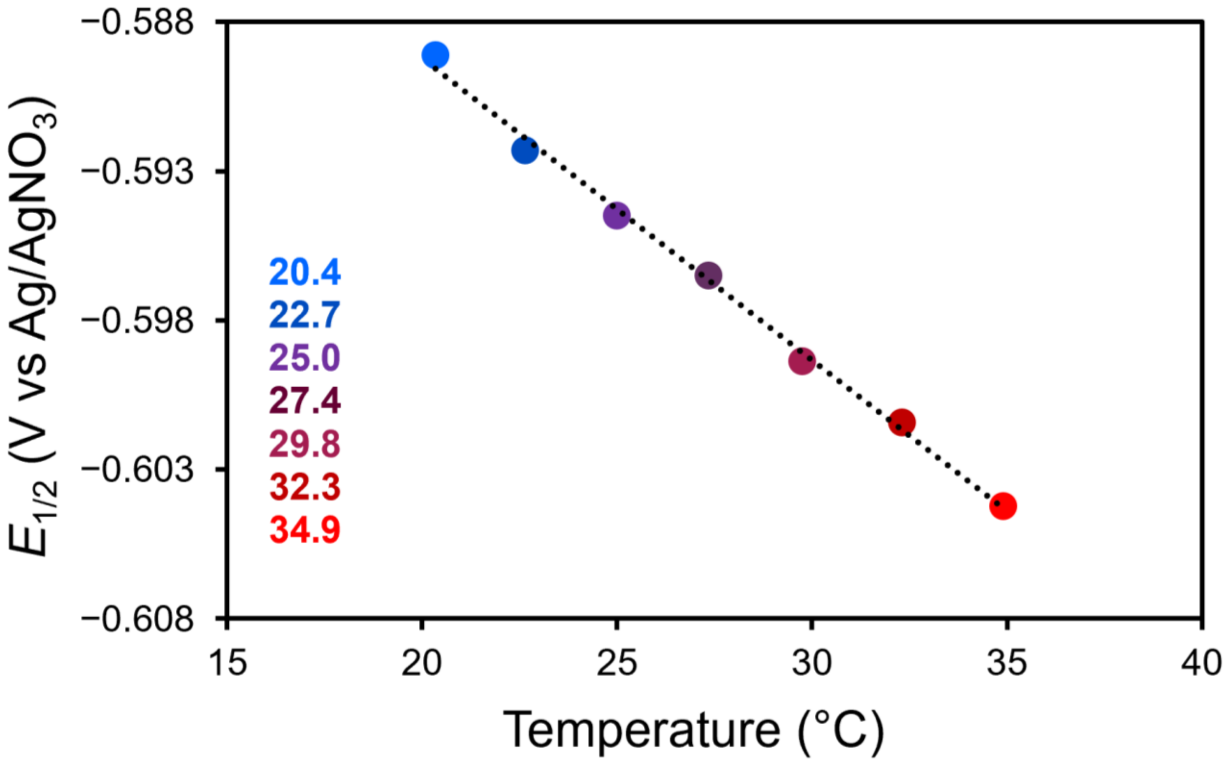

Figure S61. Plot of the temperature dependence of $E_{1/2}$ for the R1 couple of $(TBA)_3(PW_{12})$ extracted from variable-temperature CV measurements in MeCN containing 100 mM of KPF_6 supporting electrolyte (Figure 4b). Colored circles and numbers denote experimental data at their corresponding measured temperature (in °C), and the dotted black line corresponds to a linear fit to the data. The average slope from three independent measurements after correcting for the temperature coefficient of the reference electrode potential (eqs 6 and 7), which represents the average temperature coefficient, is $\alpha = -0.56(1)$ mV °C^{-1} (Table 1). The error in the average value corresponds to the standard deviation of independent measurements.

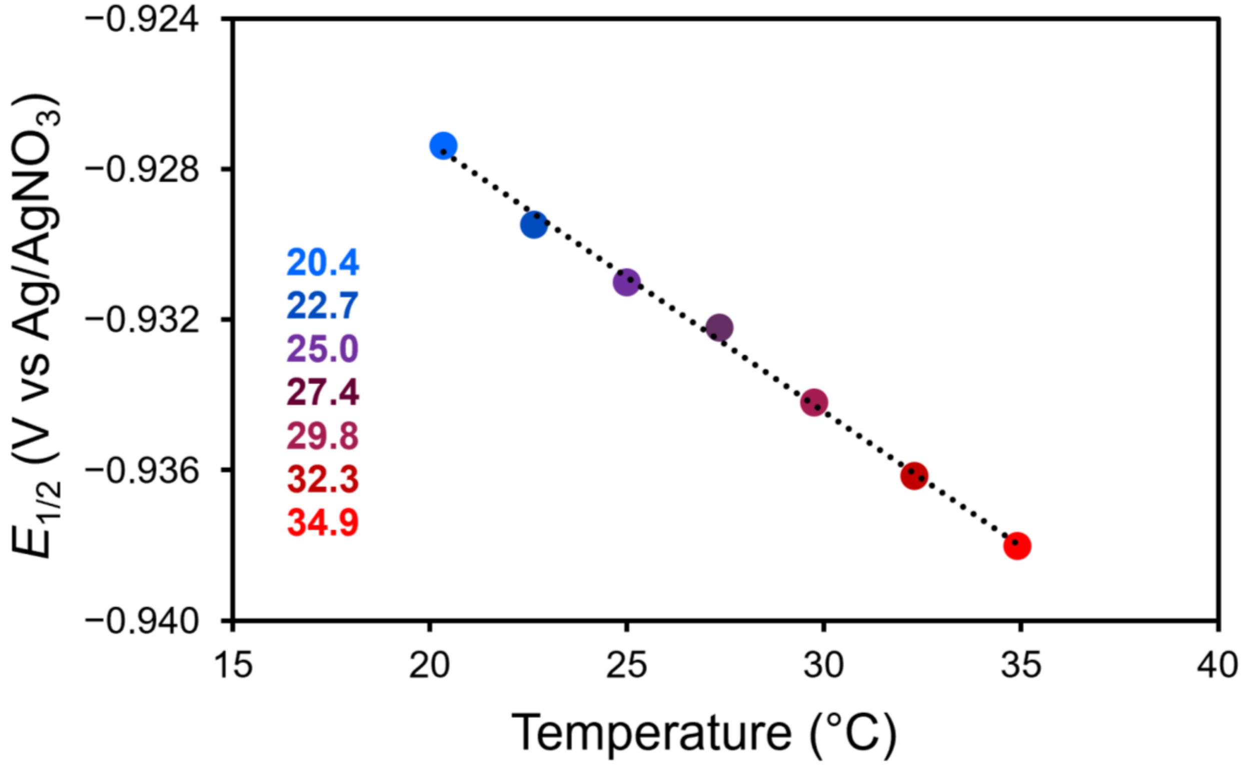

Figure S62. Plot of the temperature dependence of $E_{1/2}$ for the R2 couple of $(TBA)_3(PW_{12})$ extracted from variable-temperature CV measurements in MeCN containing 100 mM of KPF_6 supporting electrolyte (Figure 4b). Colored circles and numbers denote experimental data at their corresponding measured temperature (in °C), and the dotted black line corresponds to a linear fit to the data. The average slope from three independent measurements after correcting for the temperature coefficient of the reference electrode potential (eqs 6 and 7), which represents the average temperature coefficient, is $\alpha = -0.26(1)$ mV °C^{-1} (Table 1). The error in the average value corresponds to the standard deviation of independent measurements.

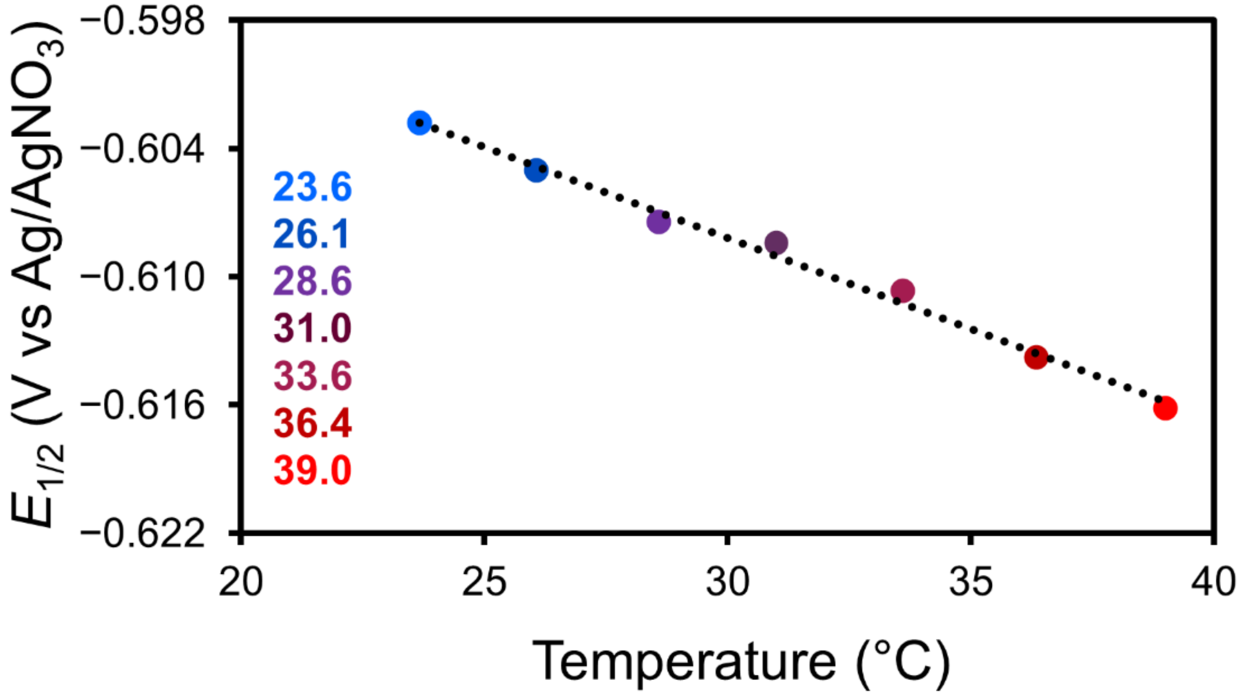

Figure S63. Plot of the temperature dependence of $E_{1/2}$ for the R1 couple of $(TBA)_3(PW_{12})$ extracted from variable-temperature CV measurements in MeCN containing 100 mM of $LiPF_6$ supporting electrolyte (Figure 4c). Colored circles and numbers denote experimental data at their corresponding measured temperature (in °C), and the dotted black line corresponds to a linear fit to the data. The average slope from three independent measurements after correcting for the temperature coefficient of the reference electrode potential (eqs 6 and 7), which represents the average temperature coefficient, is $\alpha = -0.28(1)$ mV °C^{-1} (Table 1). The error in the average value corresponds to the standard deviation of independent measurements.

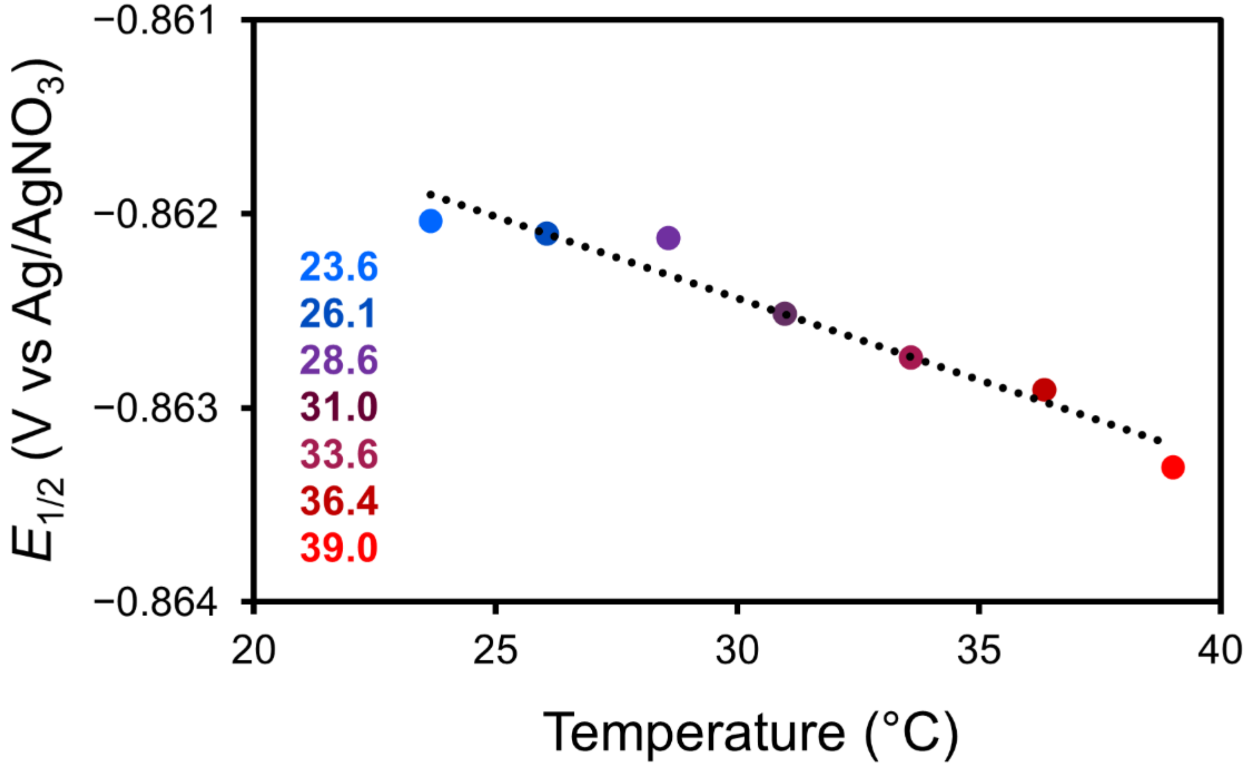

Figure S64. Plot of the temperature dependence of $E_{1/2}$ for the R2 couple of $(TBA)_3(PW_{12})$ extracted from variable-temperature CV measurements in MeCN containing 100 mM of $LiPF_6$ supporting electrolyte (Figure 4c). Colored circles and numbers denote experimental data at their corresponding measured temperature (in °C), and the dotted black line corresponds to a linear fit to the data. The average slope from three independent measurements after correcting for the temperature coefficient of the reference electrode potential (eqs 6 and 7), which represents the average temperature coefficient, is α = 0.57(1) mV °C^{-1} (Table 1). The error in the average value corresponds to the standard deviation of independent measurements.

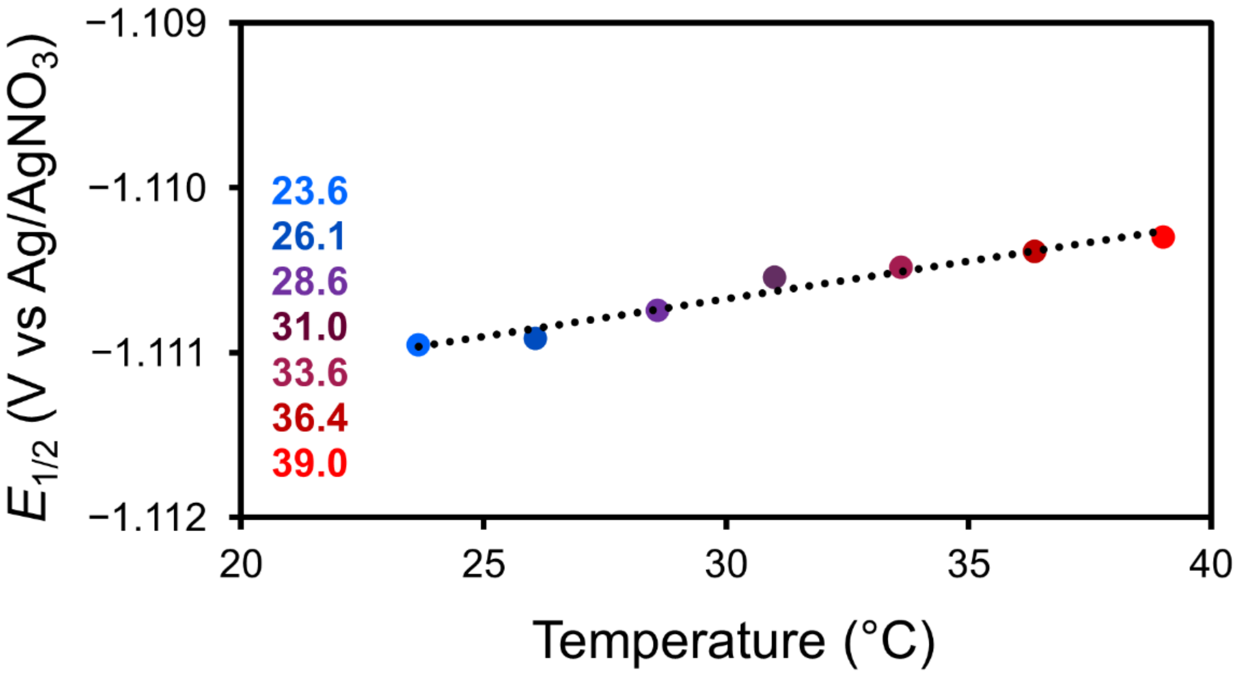

Figure S65. Plot of the temperature dependence of $E_{1/2}$ for the R3 couple of $(TBA)_3(PW_{12})$ extracted from variable-temperature CV measurements in MeCN containing 100 mM of $LiPF_6$ supporting electrolyte (Figure 4c). Colored circles and numbers denote experimental data at their corresponding measured temperature (in °C), and the dotted black line corresponds to a linear fit to the data. The average slope from three independent measurements after correcting for the temperature coefficient of the reference electrode potential (eqs 6 and 7), which represents the average temperature coefficient, is α = 0.62(6) mV °C^{-1} (Table 1). The error in the average value corresponds to the standard deviation of independent measurements.

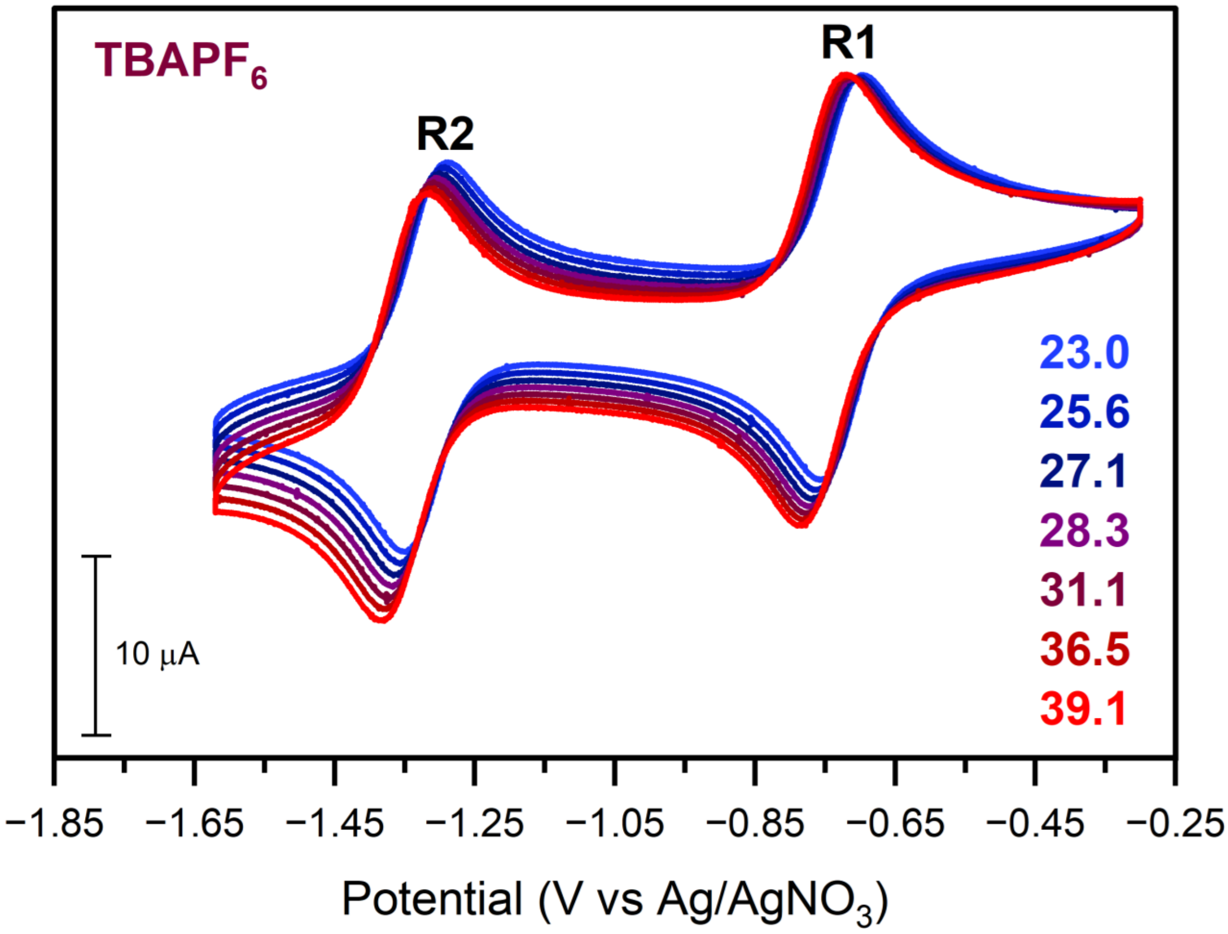

Figure S66. Variable-temperature CV data of 1 mM of $(TBA)_3(PW_{12})$ in DMF containing 100 mM of $TBAPF_6$ supporting electrolyte, collected using a scan rate of 100 mV s^{-1} in an isothermal single-compartment cell.

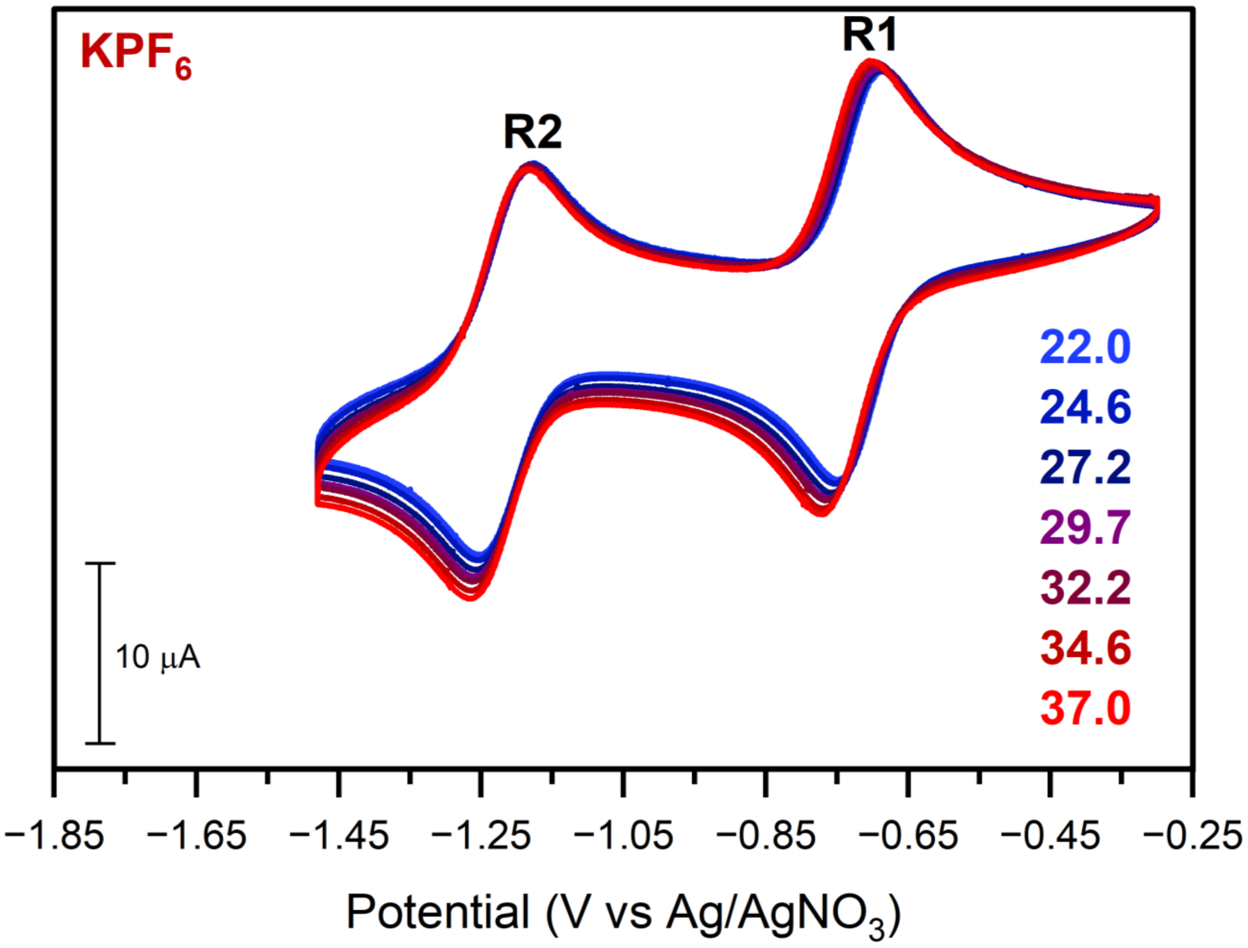

Figure S67. Variable-temperature CV data of 1 mM of $(TBA)_3(PW_{12})$ in DMF containing 100 mM of KPF_6 supporting electrolyte, collected using a scan rate of 100 mV s^{-1} in an isothermal single-compartment cell.

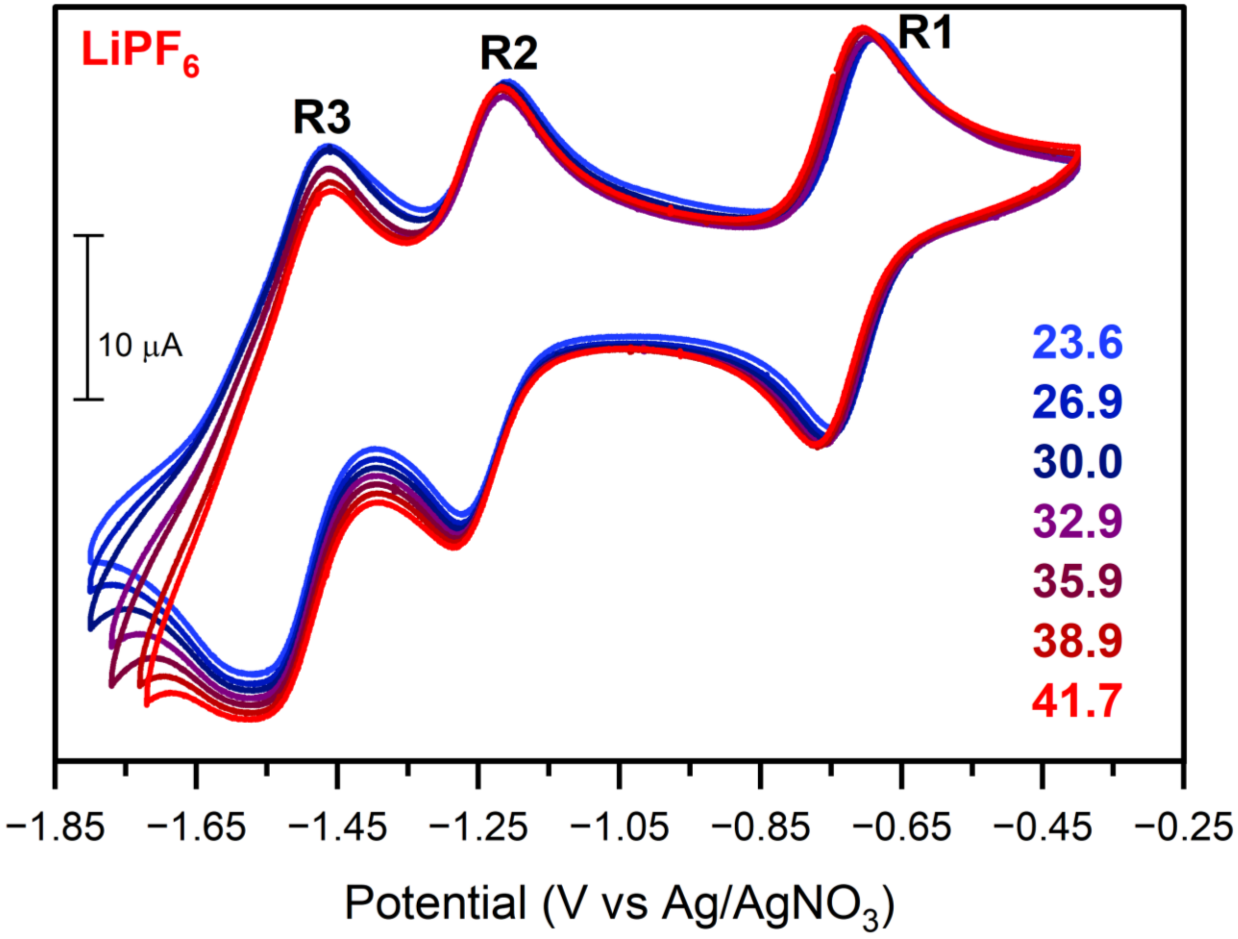

Figure S68. Variable-temperature CV data of 1 mM of $(TBA)_3(PW_{12})$ in DMF containing 100 mM of $LiPF_6$ supporting electrolyte, collected using a scan rate of 100 mV s^{-1} in an isothermal single-compartment cell.

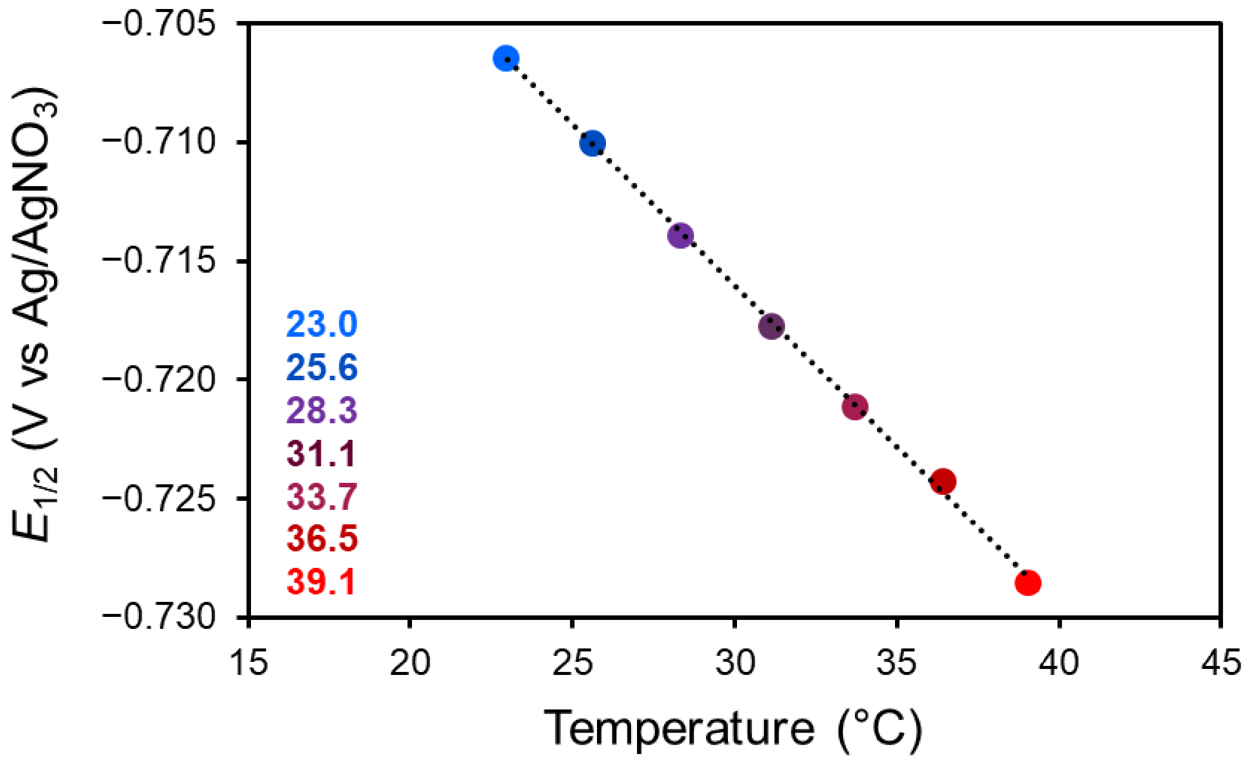

Figure S69. Plot of the temperature dependence of $E_{1/2}$ for the R1 couple of $(TBA)_3(PW_{12})$ extracted from variable-temperature CV measurements in DMF containing 100 mM of $TBAPF_6$ supporting electrolyte (Figure S66). Colored circles and numbers denote experimental data at their corresponding measured temperature (in °C), and the dotted black line corresponds to a linear fit to the data. The average slope from three independent measurements after correcting for the temperature coefficient of the reference electrode potential (eqs 6 and 7), which represents the average temperature coefficient, is $\alpha = -0.71(2)$ mV °C^{-1} (Table 2). The error in the average value corresponds to the standard deviation of independent measurements.

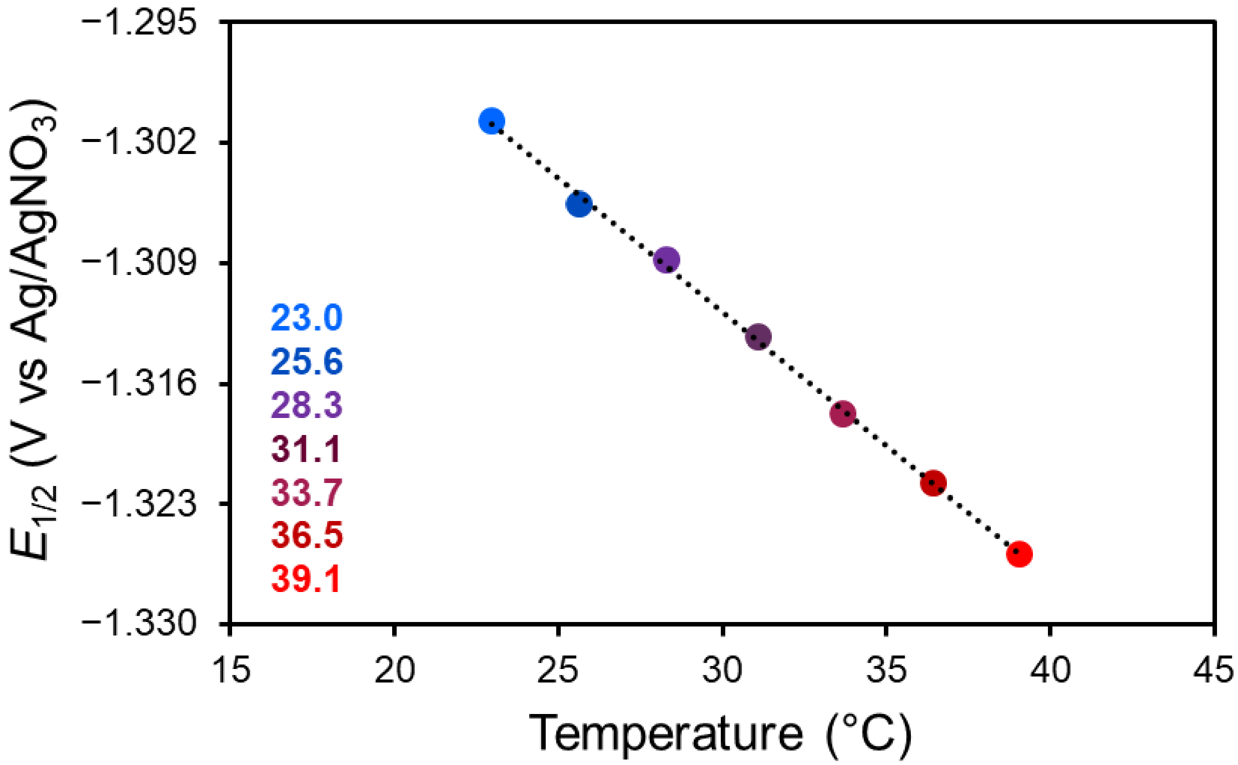

Figure S70. Plot of the temperature dependence of $E_{1/2}$ for the R2 couple of $(TBA)_3(PW_{12})$ extracted from variable-temperature CV measurements in DMF containing 100 mM of $TBAPF_6$ supporting electrolyte (Figure S66). Colored circles and numbers denote experimental data at their corresponding measured temperature (in °C), and the dotted black line corresponds to a linear fit to the data. The average slope from three independent measurements after correcting for the temperature coefficient of the reference electrode potential (eqs 6 and 7), which represents the average temperature coefficient, is $\alpha = -1.00(11)$ mV °C^{-1} (Table 2). The error in the average value corresponds to the standard deviation of independent measurements.

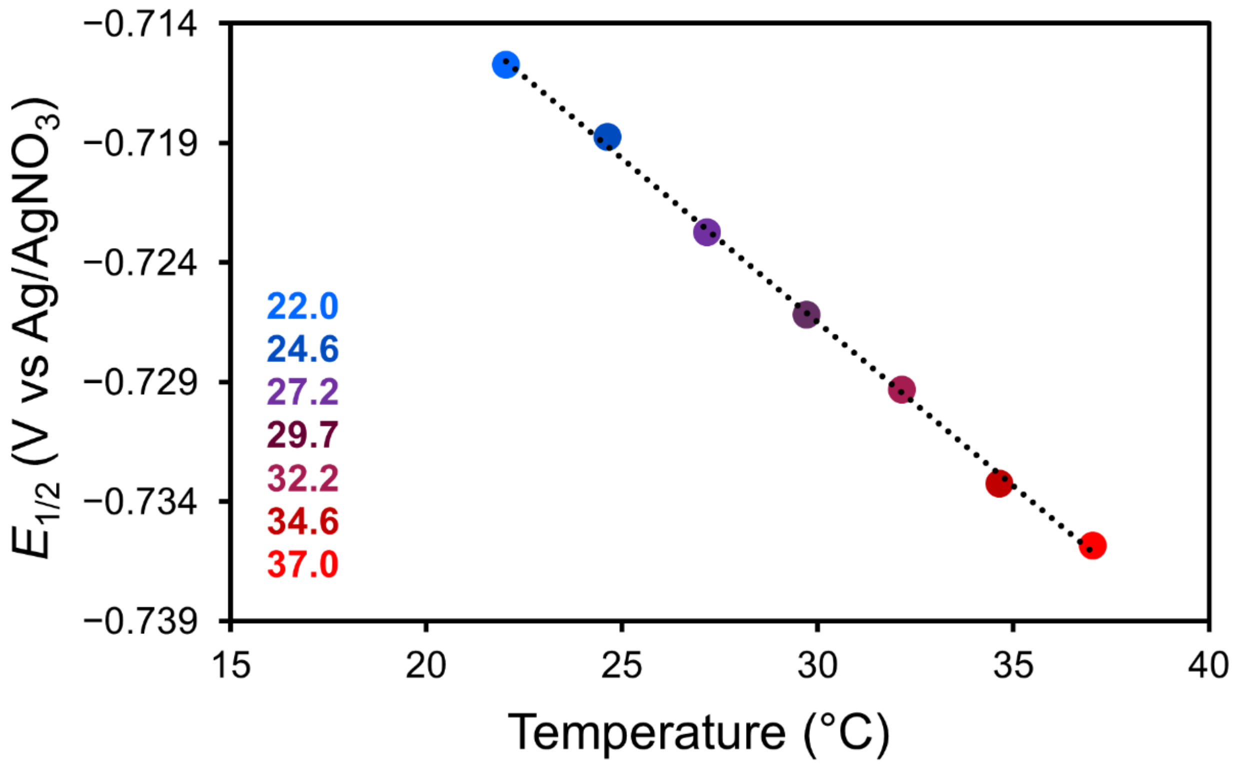

Figure S71. Plot of the temperature dependence of $E_{1/2}$ for the R1 couple of $(TBA)_3(PW_{12})$ extracted from variable-temperature CV measurements in DMF containing 100 mM of KPF_6 supporting electrolyte (Figure S67). Colored circles and numbers denote experimental data at their corresponding measured temperature (in °C), and the dotted black line corresponds to a linear fit to the data. The average slope from three independent measurements after correcting for the temperature coefficient of the reference electrode potential (eqs 6 and 7), which represents the average temperature coefficient, is $\alpha = -0.80(3)$ mV °C^{-1} (Table 2). The error in the average value corresponds to the standard deviation of independent measurements.

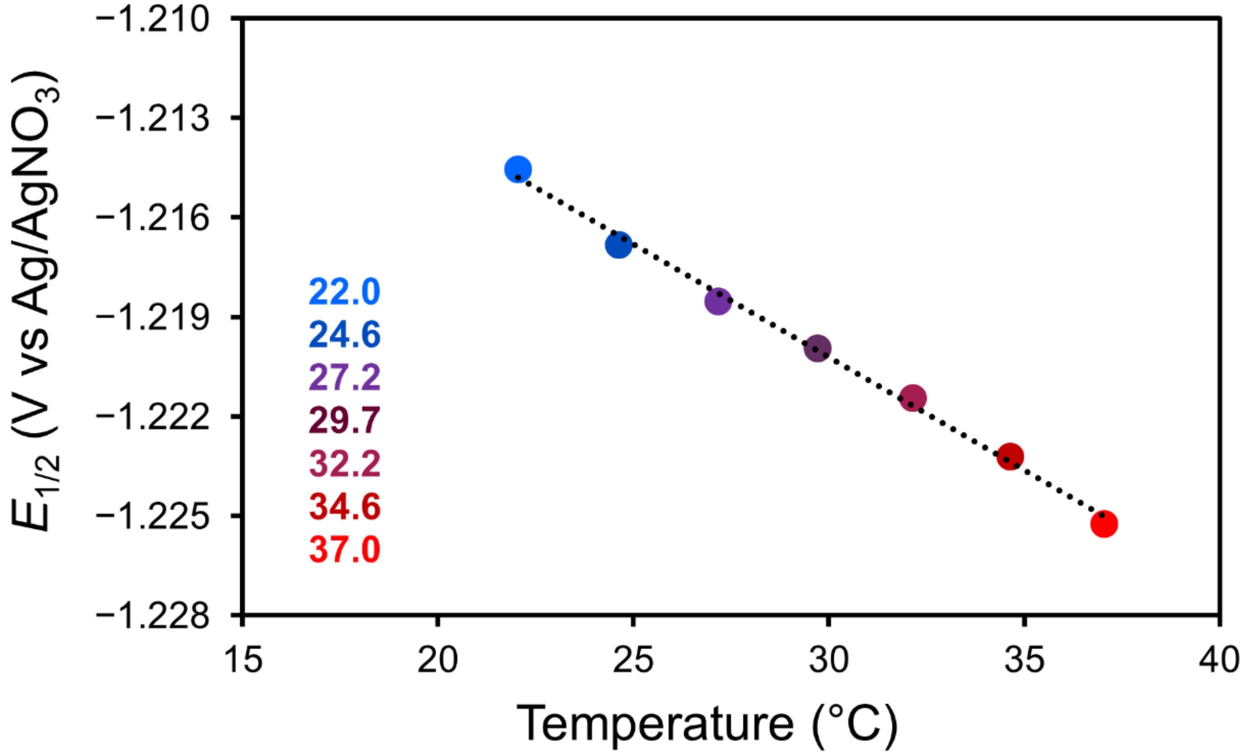

Figure S72. Plot of the temperature dependence of $E_{1/2}$ for the R2 couple of $(TBA)_3(PW_{12})$ extracted from variable-temperature CV measurements in DMF containing 100 mM of KPF_6 supporting electrolyte (Figure S67). Colored circles and numbers denote experimental data at their corresponding measured temperature (in °C), and the dotted black line corresponds to a linear fit to the data. The average slope from three independent measurements after correcting for the temperature coefficient of the reference electrode potential (eqs 6 and 7), which represents the average temperature coefficient, is $\alpha = -0.11(1)$ mV °C^{-1} (Table 2). The error in the average value corresponds to the standard deviation of independent measurements.

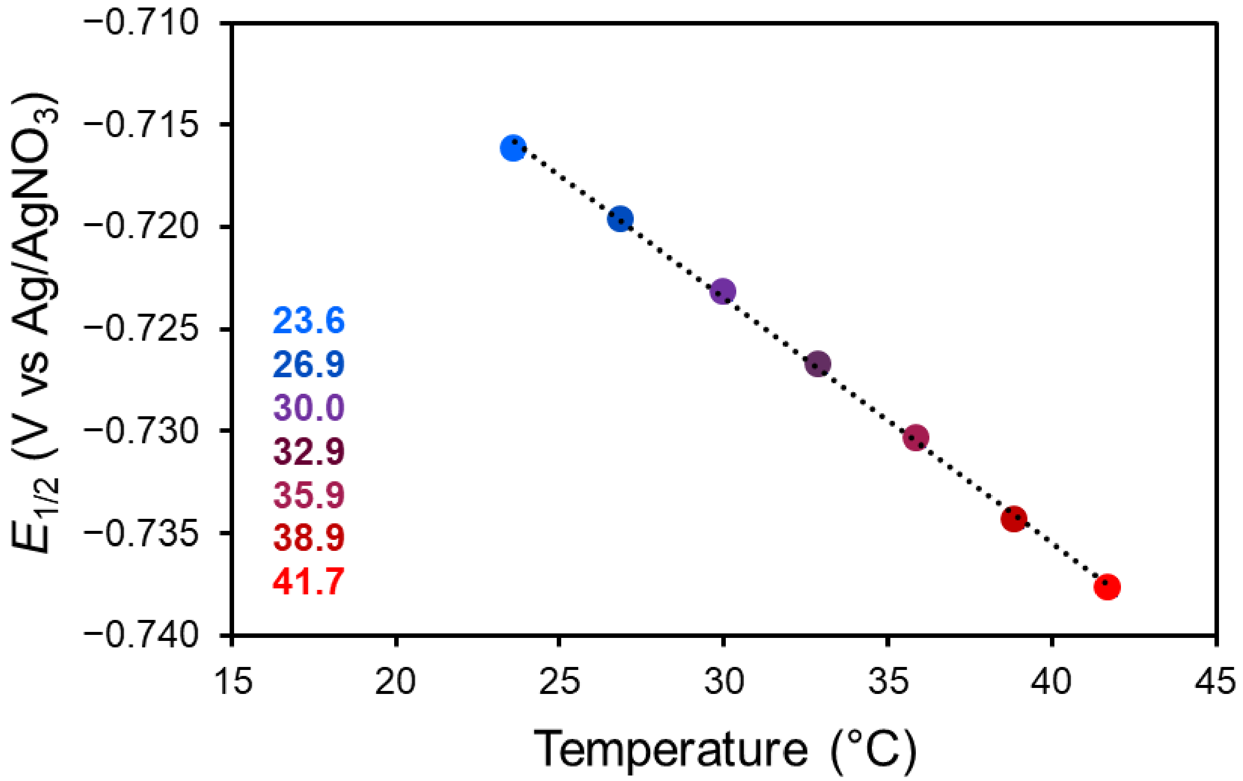

Figure S73. Plot of the temperature dependence of $E_{1/2}$ for the R1 couple of $(TBA)_3(PW_{12})$ extracted from variable-temperature CV measurements in DMF containing 100 mM of $LiPF_6$ supporting electrolyte (Figure S68). Colored circles and numbers denote experimental data at their corresponding measured temperature (in °C), and the dotted black line corresponds to a linear fit to the data. The average slope from three independent measurements after correcting for the temperature coefficient of the reference electrode potential (eqs 6 and 7), which represents the average temperature coefficient, is $\alpha = -0.47(5)$ mV °C^{-1} (Table 2). The error in the average value corresponds to the standard deviation of independent measurements.

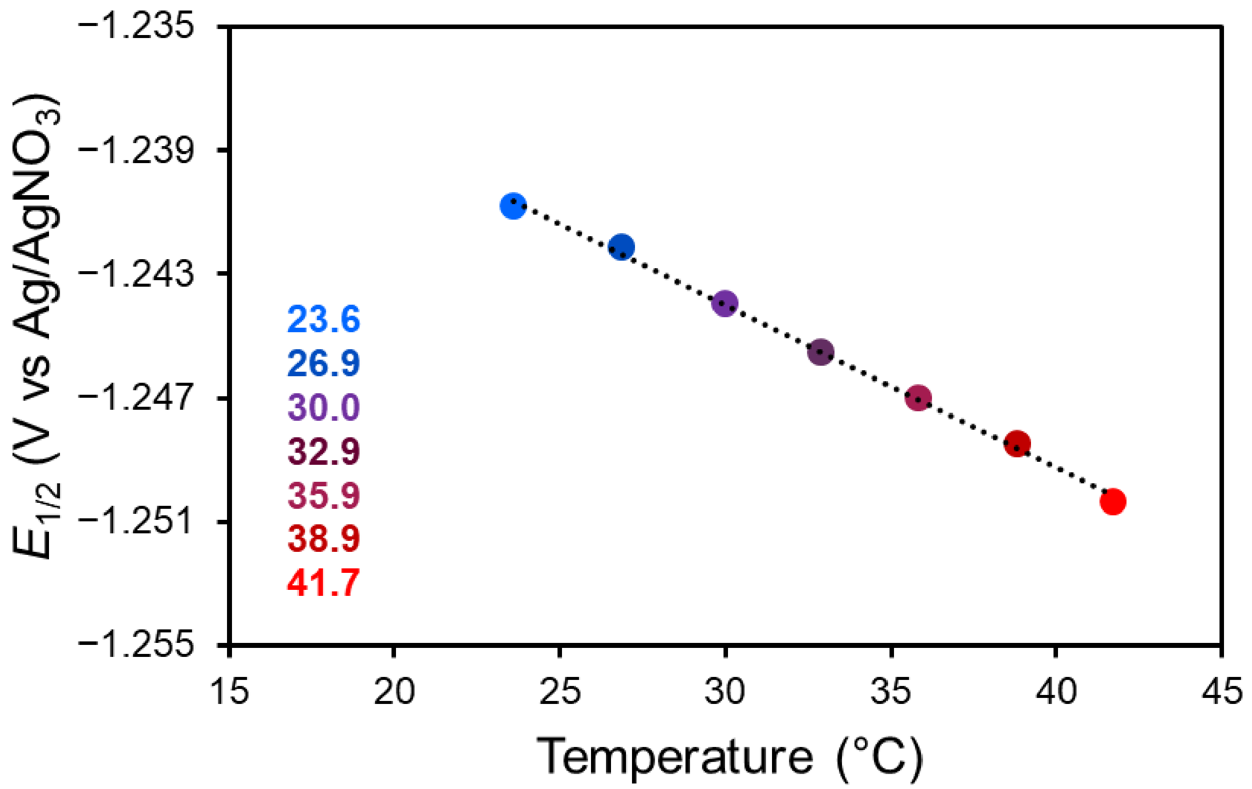

Figure S74. Plot of the temperature dependence of $E_{1/2}$ for the R2 couple of $(TBA)_3(PW_{12})$ extracted from variable-temperature CV measurements in DMF containing 100 mM of $LiPF_6$ supporting electrolyte (Figure S68). Colored circles and numbers denote experimental data at their corresponding measured temperature (in °C), and the dotted black line corresponds to a linear fit to the data. The average slope from three independent measurements after correcting for the temperature coefficient of the reference electrode potential (eqs 6 and 7), which represents the average temperature coefficient, is α = 0.21(4) mV °C^{-1} (Table 2). The error in the average value corresponds to the standard deviation of independent measurements.

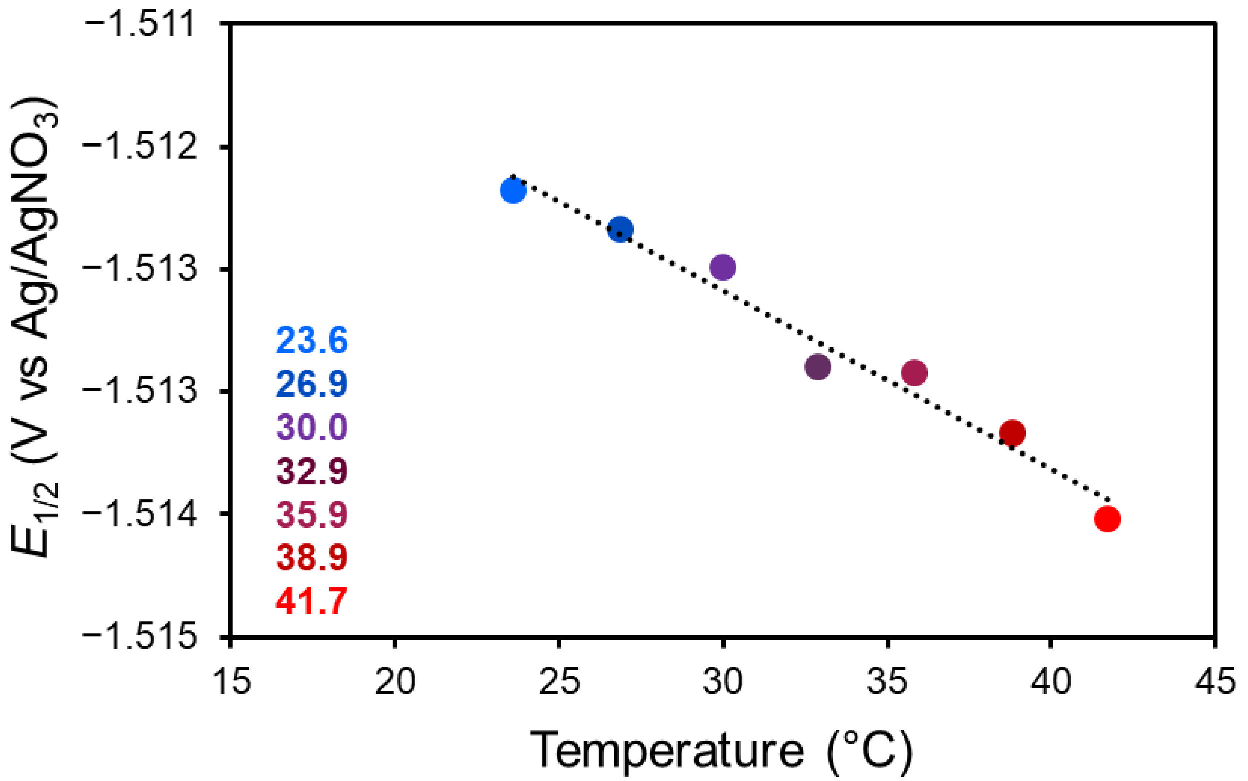

Figure S75. Plot of the temperature dependence of $E_{1/2}$ for the R3 couple of $(TBA)_3(PW_{12})$ extracted from variable-temperature CV measurements in DMF containing 100 mM of $LiPF_6$ supporting electrolyte (Figure S68). Colored circles and numbers denote experimental data at their corresponding measured temperature (in °C), and the dotted black line corresponds to a linear fit to the data. The average slope from three independent measurements after correcting for the temperature coefficient of the reference electrode potential (eqs 6 and 7), which represents the average temperature coefficient, is $\alpha = 0.58(9)$ mV $°C^{-1}$ (Table 2). The error in the average value corresponds to the standard deviation of independent measurements.

C. References

(1) Randles, J. E. B. A Cathode Ray Polarograph. Part II.—The Current-Voltage Curves. *Trans. Faraday Soc.* **1948**, *44*, 327–338. DOI: 10.1039/TF9484400327

(2) Ševčík, A. Oscillographic Polarography with Periodical Triangular Voltage. *Collect. Czech. Chem. Commun.* **1948**, *13*, 349–377. DOI: 10.1135/CCCC19480349

(3) Nicholson, R. S. Theory and Application of Cyclic Voltammetry for Measurement of Electrode Reaction Kinetics. *Anal. Chem.* **1965**, *37* (11), 1351–1355. DOI: 10.1021/ac60230a016

(4) Sawant, T. V.; McKone, J. R. Flow Battery Electroanalysis. 2. Influence of Surface Pretreatment on Fe(III/II) Redox Chemistry at Carbon Electrodes. *J. Phys. Chem. C* **2019**, *123* (1), 144–152. DOI: 10.1021/acs.jpcc.8b09607

(5) Dagar, M.; De, A.; Lu, Z.; Matson, E. M.; Thorarinsdottir, A. E. Implications of Charge and Heteroatom Dopants on the Thermodynamics and Kinetics of Redox Reactions in Keggin-Type Polyoxometalates. *ACS Mater. Au* **2025**, *5* (1), 200–210. DOI: 10.1021/acsmaterialsau.4c00136

(6) De, A.; Dagar, M.; Kneer, B.; Kim, J.; Thorarinsdottir, A. E. Best Practices for Variable-Temperature Electrochemistry Experiments and Data Reporting. *ACS Energy Lett.* **2025**, *10* (4), 1542–1549. DOI: 10.1021/acsenergylett.5c00308
